# Sea‐Ice Retreat From the Northeast Greenland Continental Shelf Triggers a Marine Trophic Cascade

**DOI:** 10.1111/gcb.70189

**Published:** 2025-04-24

**Authors:** Jack H. Laverick, Douglas C. Speirs, Michael R. Heath

**Affiliations:** ^1^ University of Strathclyde Glasgow UK

**Keywords:** Arctic, climate change, food web, Greenland, marine, mathematical modelling, production, StrathE2E, trophic Cascade

## Abstract

Climate change is causing sea‐ice to retreat from Arctic ecosystems. Loss of ice impacts the ecosystem in many ways, reducing habitat area for specialist species like polar bears, releasing freshwater and nutrients, and increasing light penetration into the water column. To explore the interaction of these effects, we implemented a Northeast Greenland continental shelf parameterisation of the end‐to‐end ecosystem model StrathE2E. We used model output from the NEMO‐MEDUSA ocean‐biogeochemistry model under Representative Concentration Pathway 8.5 as driving data, which suggests the northeast Greenland continental shelf will become seasonally ice‐free by 2050. We simulated half a century of climate change by running the model system to a set of steady states for each decade from the 2010s to the 2050s. Our simulations show sea‐ice retreat from the northeast Greenland continental shelf boosts the productivity of the marine food web. Total living mass increases by over 25%, with proportionally larger increases for higher trophic levels. The exception to this is a 66% reduction in maritime mammal mass. Additional network indices reveal that the ecosystem becomes more mature, with future diets more specialized and a lengthening of the food web. Our model provides long‐term strategic insight for the management of the northeast Greenland continental shelf, allowing for the quantitative evaluation of conservation goals and the scale of prospective fisheries. Our results present a mixed picture for the future of the Arctic, with growing populations for fish and charismatic megafauna like cetaceans accompanied by the loss of endemic biodiversity such as polar bears.

## Graphical abstract

We built a computer model of the marine food web for the continental shelf off northeast Greenland. As climate change melts sea‐ice, more light will penetrate the water column. This triggers a cascade of increasing biomass in the system from plankton to fish to whales, but a loss of habitat means fewer polar bears.

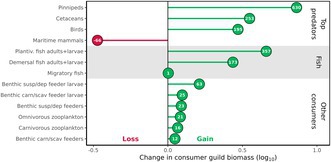

## 1 Introduction

Climate change is progressing more rapidly at the poles than anywhere else on Earth (Clem et al. 2020; Holland and Bitz 2003; Koenigk et al. 2020). As well as the planetary‐scale consequences, such as rising sea levels (Hofer et al. 2020) and the potential disruption to the Atlantic meridional overturning current (Sévellec et al. 2017), there will be localized changes worthy of consideration. The proportion of thick, multi‐year sea ice has more than halved since 2002 (Kwok 2018), with the Arctic potentially experiencing ice‐free conditions by 2050 (Thackeray and Hall 2019). This points to a radically different marine environment in the future, freshening the ocean (Shu et al. 2018) as sea surface temperatures rise (Carvalho and Wang 2020; Yang et al. 2023).

The loss of sea ice will be disruptive, affecting important processes relevant to ecology such as the subsurface penetration of sunlight (Castellani et al. 2022) and delivery of nutrients (Tovar‐Sánchez et al. 2010). These environmental changes will likely have knock‐on consequences for the marine ecosystem. We may expect retreating sea ice to increase the productivity of the system (Arrigo and van Dijken 2015; Brandt et al. 2023; Castagno et al. 2023; Hansen et al. 2003), as sunlight and nutrients are both required for primary production. Classical food web theory would suggest this could drive a bottom‐up trophic cascade, supporting greater biomass at higher trophic levels (Heath et al. 2014).

Due to the complexities of Arctic food webs, with migration and hibernation being key strategies to survive the polar night, it is unclear how newly mobilised biomass will be distributed within the Arctic or to what scales. Some taxa are dependent on sea‐ice for their survival (Johnson et al. 2020), and indirect effects within the food web, such as competition, can create winners and losers under different environmental conditions (Kortsch et al. 2019; McMeans et al. 2013; Miller et al. 2015).

The northeast Greenland continental shelf is a particular case in point. The East Greenland Coastal Current is a major export route for sea ice from the Arctic (Bacon et al. 2014) maintaining almost permanent ice cover over the northeast Greenland continental shelf. However, climate models indicate that this is set to change and that the continental shelf is likely to become seasonally ice‐free by the 2050s, regardless of the trajectory of future CO_2_ emissions (Yool et al. 2015). As sea ice retreats in the region, a novel ecosystem will emerge, one which will become accessible to exploitation comparable to adjacent areas of the northeast Atlantic (Eguíluz et al. 2016; Ng et al. 2018; Troell et al. 2017). It would be prudent to understand how the system may evolve to allow for the effective management of the region (Perissi et al. 2017; Troell et al. 2017), given it is in one of the least studied ICES (International Council for the Exploration of the Sea) eco‐regions (ICES 2020).

In this paper we describe an implementation of the end‐to‐end ecosystem model StrathE2EPolar (Heath et al. 2022) for the Northeast Greenland continental shelf. The new implementation is primarily driven by output from the NEMO‐MEDUSA ocean‐biogeochemistry model (Yool et al. 2013, 2015) which provides climate projections for the region. We use this model setup to investigate the possible impacts of half a century of climate change on the marine environment and to characterise the future state of the food web.

## 2 Materials and Methods

### 2.1 Model Background

StrathE2EPolar (v2.1.0) is an extension of the temperate end‐to‐end ecosystem model StrathE2E2 (Heath et al. 2021). Both models are available as R packages (https://www.marineresourcemodelling.maths.strath.ac.uk/strathe2e/index.html; https://www.marineresourcemodelling.maths.strath.ac.uk/strathe2epolar/index.html). The model tracks the flows and stocks of nitrogen through physical, chemical, and biological processes in marine continental shelf ecosystems. In contrast to other typical existing models, such as Ecopath with Ecosim (Heymans et al. 2016; Keramidas et al. 2023; Plagányi and Butterworth 2004), StrathE2EPolar outputs data at daily intervals, which is necessary to represent seasonal changes in sea ice and primary production. StrathE2E models also allow for feedback effects from the broader ecosystem onto rates of primary production; primary production is an emergent property rather than a boundary condition.

**FIGURE 1:**
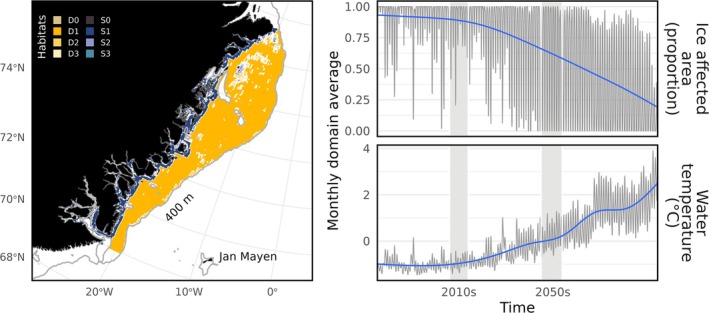
Summary of the Northeast Greenland Continental Shelf implementation of StrathE2EPolar. The model represents eight seabed habitat classes (left panel) as the cross between two depth layers (shallow and deep) and four seabed sediment classes (0 = Rock, 1 = Mud, 2 = Sand, 3 = Gravel). This map was used to parameterise the physical parameters of the seabed habitats in our box model, as well as various driving data. Right panels show time series of NEMO‐MEDUSA model output from 1980 to 2099 under climate scenario RCP8.5. The first and last decades used for StrathE2EPolar driving data are marked by grey rectangles. The first decade is characterised by sub‐zero temperatures and persistent sea‐ice. By the 2050s, temperatures are reliably seasonally above 0, with the area seasonally ice‐free in summer.

Full details of the spatial arrangement, biological and chemical components of the network, and underlying equations can be accessed in previous publications (Heath et al. 2021, 2022) and the R package documentation. StrathE2E models are box models comprised of a set of coupled ordinary differential equations which track the exchange of nitrogen mass through a network of food web guilds spanning dissolved material, detritus, and microbes, through to top predators such as birds and mammals (Figure S1). Space is represented by a coarse box structure of three ocean volumes (inshore, offshore shallow, offshore deep) with associated seabed habitats (Figure 1).

StrathE2EPolar adds guilds to the temperate StrathE2E2 model. These include nitrate and ammonia in snow and ice, ice‐bound detritus, ice algae, and maritime mammals (polar bears and arctic foxes). Other arctic species such as Narwhals and Walruses are represented in existing cetacean and pinniped guilds. Additional environmental drivers including sea ice concentration and thickness limit the habitat area accessible to maritime mammals, cetaceans, and pinnipeds, while also attenuating the light reaching the water column.

The simplicity in taxonomic and spatial structure of StrathE2E models allows for extensive experimentation and for the system to be run to steady states. This process removes the influence of initial conditions when comparing model results. To support this, we drive StathE2E models using datasets averaged across multiple years, typically for different decades. In these cases, the steady state of the modelled system represents the attractor for each future decade.

### 2.2 The Northeast Greenland Continental Shelf Implementation

All data processing to create the Northeast Greenland continental shelf implementation, and all subsequent analyses, were performed in the R programming environment (R‐Core‐Team 2019). An existing, calibrated Barents Sea implementation of StrathE2EPolar for the period 2011–2019 (Heath et al. 2022) provided the basis for the Greenland implementation (Laverick et al. 2025a). The two model domains are geographically close but contain differing fishing and sea ice regimes. We therefore updated the environmental driving data, physical parameters, and set the activity rates for all fishing gears to zero since fisheries monitoring data showed negligible catches and effort during the 2010s. In the absence of fishing activity, this implementation for the Northeast Greenland continental shelf allows for investigation of the “natural” ecosystem state.

A detailed explanation of the data processing to build the Greenland implementation is available in the Data S1. These implementation documents are also available through the StrathE2E2 and StrathE2EPolar websites, where other implementation files can be downloaded. The code for the data processing is available through PURE (Laverick 2025) and Github (https://github.com/Jack‐H‐Laverick/MiMeMo.EastGreenlandShelf).

In brief, driving data were extracted from the sources in Table 1 and averaged across a baseline time period of 2011 to 2019 (the 2010s) into monthly climatological cycles for each variable. Model variants representing future time periods were created by extracting data from sources providing future projections under Representative Concentration Pathway (RCP) 8.5. RCP8.5 represents an extreme emissions scenario, suggesting a global temperature increase of about 4.3°C by 2100 (Pörtner et al. 2019). Consequently, RCP8.5 predicts seasonally ice‐free conditions earlier this century than other emissions scenarios. Driving data from sources without projections were held constant across all time variants. We created four decadal future time variants from 2020–2029 up to 2050–2059. All time variants were run to a steady state, verified by visual inspection of an ensemble of outputted time series. The physical set up of the model domain was parametrised according to synthetic sediment maps of the region (Laverick et al. 2023).

**TABLE 1:** Sources of environmental driving data for the Northeast Greenland continental shelf implementation.

Source	Variables	Projections
0.25° NEMO‐MEDUSA (Yool et al. 2013, 2015) under RCP8.5	Water temperature Vertical diffusivity at the interface between vertical layers in the offshore zone Ice (and snow) extent, cover, and thickness Daily integrated incident irradiance Daily integrated ocean and river water inflow volumes across the external boundaries Boundary concentrations of dissolved inorganic nitrogen (DIN), detritus and phytoplankton	Yes
CERA‐20C ‘Ocean Wave Synoptic Monthly Means’ product accessed through ECMWF	Significant wave height in the inshore zone	No
EMEP data centre (https://www.emep.int/mscw/mscw_moddata.html)	Monthly averaged annual cycles of wet and dry atmospheric nutrient deposition rates	No
Extracted from Figure 2 and Figure S5 of Wadham et al. (2016) using webplot digitizer	Meltwater nutrient concentrations	No
Remote sensing data (Globcolour L3b; ftp://ftp.hermes.acri.fr/GLOB/merged/month/)	Suspended particulate matter (SPM) in the inshore zone and upper layer of the offshore zone	No

### 2.3 Model Metrics

As a high‐level summary of the changing ecosystem, we explored the shifting influence of modes of nutrition within the marine food web. We used the flow matrix, which excludes demographic processes (fish spawning and larval stages) from StrathE2EPolar. Primary production was calculated as the sum of flows into the macrophyte, phytoplankton, and ice algae guilds. Recycling, regarded here as detritivory, was calculated as the sum of flows from detrital compartments into living model compartments, excluding primary production. Consumption was calculated as the sum of flows from living compartments into other living compartments. These three processes account for all live activity in the model, and so values were scaled as percentages to aid assessment of the relative shares of live activity. We also calculated the standing nitrogen mass of guilds responsible for primary production, recycling, and consumption. When a guild engaged in both recycling and consumption (scavengers/omnivores) the mass was shared according to the ratio of the two types of flow through the guild.

To capture half a century of change on the northeast Greenland continental shelf, we compared the average annual mass of consumer guilds in the 2010s and 2050s. The 2010s model provides the baseline for change. To account for the uneven distribution of mass through the food web, we report both the proportional change on a log_10_ scale and the percentage change by the 2050s.

StrathE2EPolar uses the R package NetIndices (Kones et al. 2009) to output a collection of food web indices. We only report on a subset in this manuscript relevant to assessing food web maturity (Margalef 1968; Odum 1969; Pérez‐Espaa and Arreguín‐Sánchez 2001; Ulanowicz 2000): trophic level, omnivory index, internal capacity, internal ascendancy capacity ratio, dominance of indirect effects. The equations for these indices can be found in the NetIndices vignette, and further details are provided in it. We calculated the mass‐weighted mean trophic level of top predators (maritime mammals, pinnipeds, cetaceans, seabirds) and the mass‐weighted mean omnivory index across all consumer guilds. These metrics allow us to assess the distribution of mass and biological activity across the food web Table 2.

**TABLE 2:** Description of presented network indices.

Metric	Units	Description
Trophic level	Trophic level	The weighted average of food source trophic levels, where weights are the proportion of a consumer's diet satisfied by a particular food source
Omnivory index	Trophic level	Sum of the squared differences in trophic level between consumer and food sources, scaled by the proportion of the consumer diet satisfied by a food source. Smaller numbers indicate more specialised diets
Internal capacity	mmolN‐bits/m^2^/d	The diversity of connections in the food web scaled by the total system throughput. Internal as flows outside of the food web are ignored. The most rigidly organised food web possible. Larger numbers indicate a more organised system
Internal ascendancy capacity ratio	Ratio (dimensionless)	The food web's realised size and organisation expressed as a proportion of the theoretical maximum (capacity) achievable for the food web. Internal as flows outside of the food web are ignored. Larger numbers indicate a system approaching the limits of development
Dominance of indirect effects	Ratio (dimensionless)	The total of indirect mass contributions to food web components (through pathways of length greater than one) divided by the total food web direct flow intensity (paths of length 1). Larger numbers indicate a more complex system

*Note:* These indices are described in the NetIndices vignette on CRAN, complete with equations and supporting references.

### 2.4 Causal Inference

The climate projections for each decade derived from NEMO‐MEDUSA model output contain multiple co‐varying drivers (Table 1). To identify the causative agents of ecosystem‐level changes, we conducted a series of five knock‐out experiments (Table 3). For these experiments, the set of model drivers in Table 3 were held at the values forcing the 2010s period; other drivers were allowed to change according to climate projections. The resulting sets of environmental driving data are themselves improbable, that is, unchanged sea ice as the ocean and air warms; however, they allow us to identify specific aspects of environmental change which are causative of different ecosystem states.

**TABLE 3:** Definitions of experimental conditions for climate‐related causal inference.

Experiment	Drivers held constant
Boundary	Concentrations of nitrogen mass sources at the model boundary SO_nitrate SO_ammonia SO_phyt SO_detritus D_nitrate D_ammonia D_phyt D_detritus SI_nitrate SI_ammonia SI_phyt SI_detritus
Flows	Water volume exchanges for all model compartments SO_OceanIN D_OceanIN SI_OceanIN SI_OceanOUT SO_SI_flow
Ice	Variables related to the StrathE2EPolar cryosphere module SO_IceFree SI_IceFree SO_IceCover SI_IceCover SO_IceThickness SI_IceThickness SO_SnowThickness SI_SnowThickness
Light	Surface irradiance Slight
Temperature	Air and ocean temperatures for all compartments SO_temp D_temp SI_temp SO_AirTemp SI_AirTemp

*Note:* SO, SI, and D indicate the surface offshore, surface inshore, and deep model compartments respectively. For each listed experiment in the table, the drivers mentioned were held at the values for the 2010 decadal period; all other drivers were updated to the relevant time period extracted from the sources in Table 1.

Each experimental condition was set to run for 200 years to achieve a steady state. The whole domain annualized masses were extracted from each model run, including those subject to full climate forcing. The masses were then scaled and passed to principal component analysis using the vegan package (Oksanen et al. 2022).

### 2.5 Sensitivity Analyses

We conducted full sensitivity analyses to all model parameters (Morris et al. 2014; Morris 1991; Wu et al. 2013) on the phytoplankton F‐ratio (the proportion of total DIN uptake in the form of nitrate, a measure of non‐recycled production, Eppley and Peterson 1979) and phytoplankton net primary production in the 2010s Greenland model. This is to illustrate the robustness of the model's findings in the face of borrowed parameter values from the Barents Sea implementation (Heath et al. 2022). We selected these two metrics as preceding analyses indicate a bottom‐up trophic cascade, so parameters that primary production is sensitive to will affect the whole ecosystem. The sensitivity analyses were performed on the 2010s model variant only, as all parameters were shared across models. The environmental forcings do change between decades under climate projections; their influence is further investigated using knock‐out experiments.

## 3 Results

According to the NEMO‐MEDUSA model output, the northeast Greenland continental shelf is projected to be seasonally ice free by the 2050s (Figure 1). Melting sea ice caused the mass of nutrients locked in snow and ice to decrease by 68.25% from the 2010s to the 2050s. Melting snow and ice also caused the proportion of light reaching the ocean surface over the course of a year to change from 16.7% in the 2010s to 53.7% in the 2050s. The net export of nutrients across the model boundary increased by 21.45%.

### 3.1 Modes of Nutrition

**FIGURE 2:**
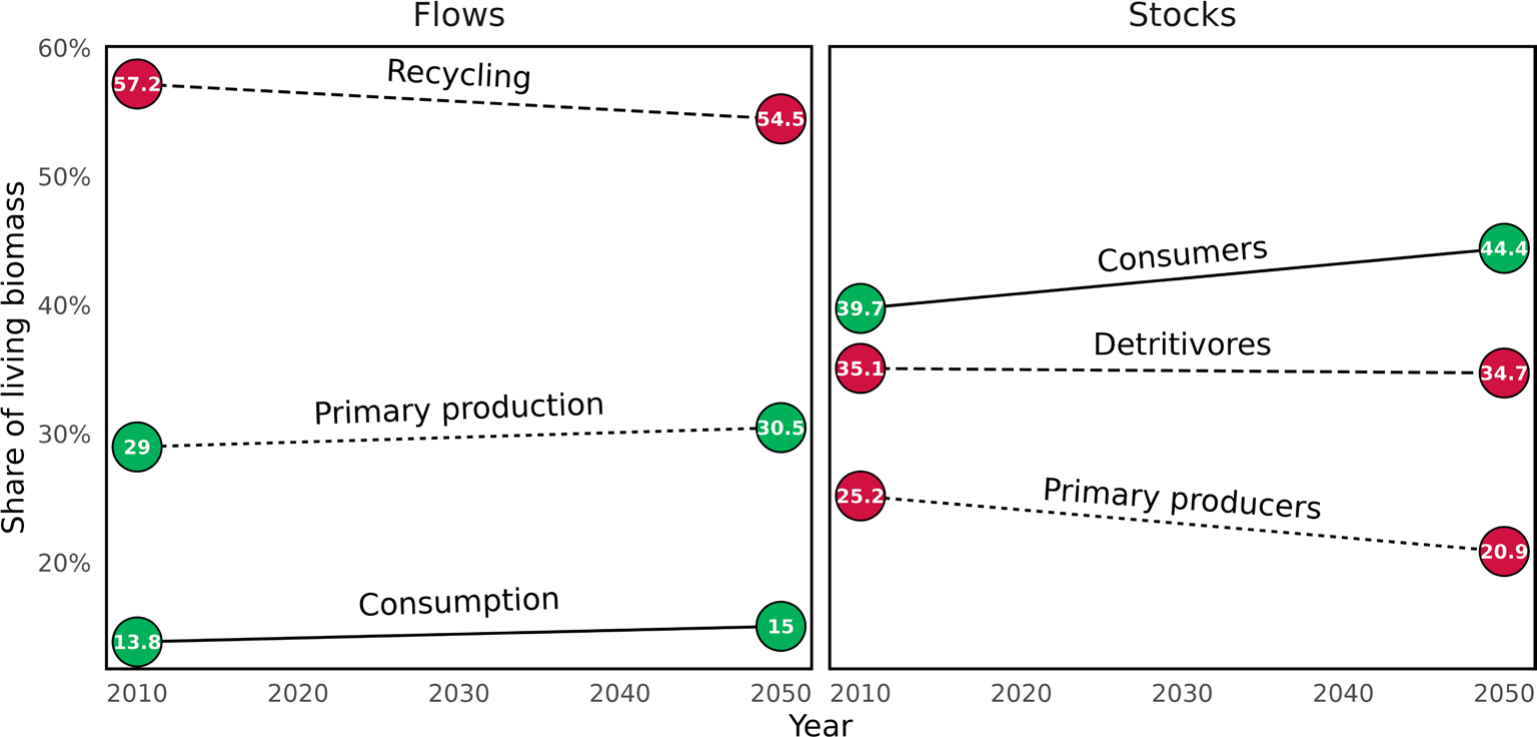
Change in share of major groups of StrathE2EPolar's living subnetwork. Values over points are the share (%) at each time point. Decreases over time are shown in red, increases are in green.

Biological activity, the total living flows in the food web, increased by 39.37%. Over time, the relative importance of recycling processes decreased from 57.2% to 54.5% of all biological activity (Figure 2), with increases in the importance of primary production (despite a loss of ice algae) and consumption.

Increased biological activity was accompanied by increases in total living mass of 25.58%. The relative importance of detritivores, in terms of share of mass, exhibited little change. Consumer mass increased from 39.7% to 44.4%, at the expense of primary producers, as increases in primary production were funneled up the food web.

### 3.2 Consumer Mass

**FIGURE 3:**
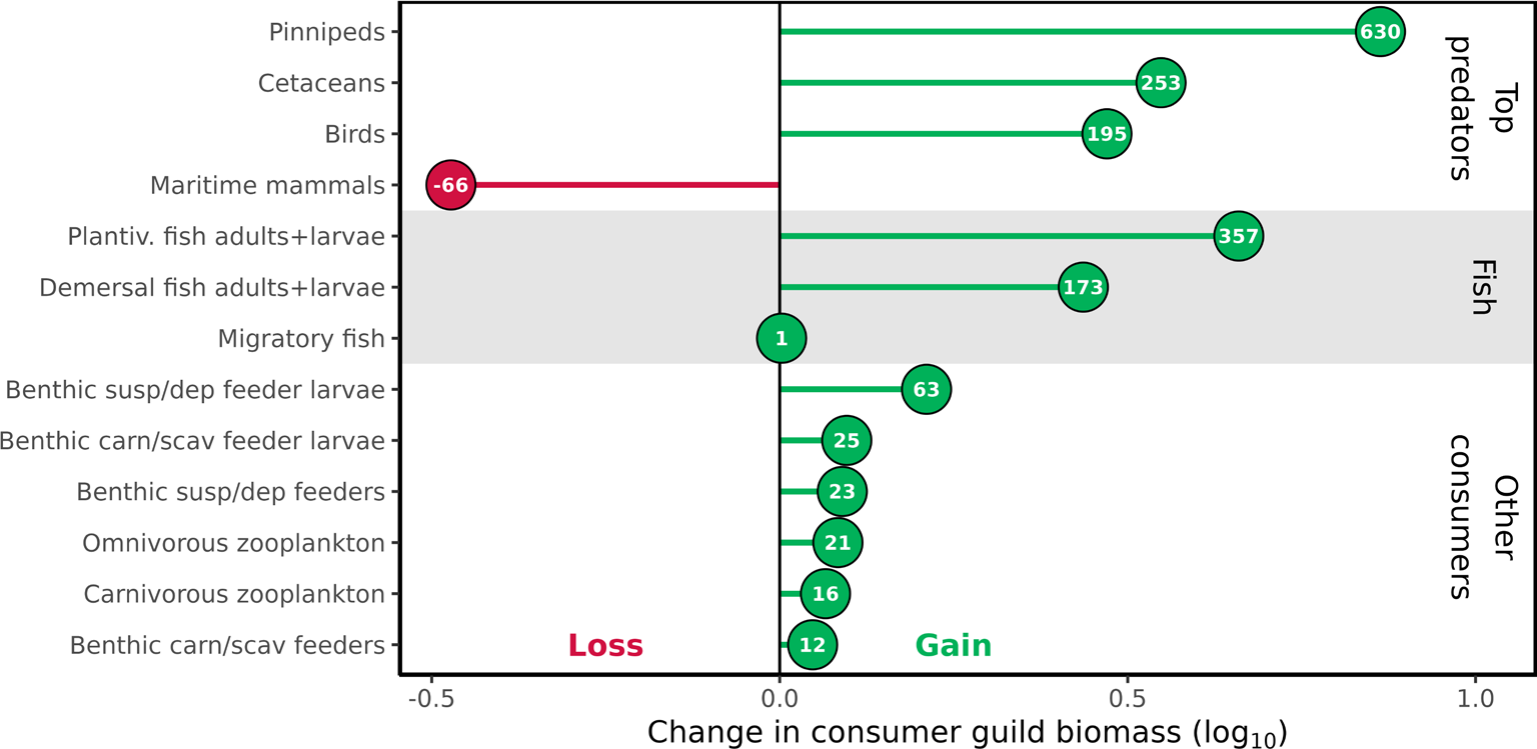
Change in mass of consumer guilds from the 2010s to the 2050s. Values over points are the change in % from guild mass in the 2010s. The *x*‐axis shows the proportional change on a log_10_ scale (scenario/baseline). Guilds at higher trophic levels gain more mass, except for Maritime mammals (polar bears).

To investigate how increased primary production is transferred up the food web, we calculated the change in mass of consumer guilds from the 2010s to the 2050s. Absolute annual mean masses for guilds in the 2010s and 2050s are presented in Table 4, including estimates from the Barents Sea in the 2010s as a sense‐check. Guilds at higher trophic levels, such as top predators and fish, gained proportionally more mass than other consumers (Figure 3).

**TABLE 4:** Absolute guild nitrogen masses in the 2010s and 2050s.

Guild	GL 2010s	GL 2050s	BS 2010s
Birds	5.048656e‐03	1.490990e‐02	1.237110e‐02
Pinnipeds	1.062421e‐02	7.754153e‐02	7.148973e‐02
Maritime mammals	1.417246e‐02	4.774963e‐03	2.516999e‐03
Migratory fish	6.195191e‐02	6.233540e‐02	6.320373e‐02
Ice algae	6.258407e‐02	5.316104e‐02	6.388779e‐02
Cetaceans	8.885116e‐02	3.137842e‐01	4.532786e‐01
Demersal fish larvae	9.627418e‐02	2.675364e‐01	7.227460e‐01
Planktivorous fish larvae	9.637280e‐02	4.328851e‐01	2.331033e‐01
Snow ammonia	2.248572e‐01	8.568849e‐02	8.928734e‐02
Snow nitrate	3.265977e‐01	1.245446e‐01	7.936141e‐02
Ice ammonia	4.754215e‐01	5.340487e‐02	9.325340e‐02
Benthos carn/scav feeders larvae	5.484466e‐01	6.846217e‐01	7.100788e‐01
Benthos susp/dep feeders larvae	5.790206e‐01	9.411860e‐01	1.043274e+00
Ice nitrate	7.150457e‐01	2.891845e‐01	1.922444e‐02
Ice detritus	7.873267e‐01	8.152309e‐01	1.119108e+00
Sediment porewater nitrate	9.893880e‐01	7.307051e‐01	9.118393e‐01
Deep layer phytoplankton	1.092862e+00	1.398580e+00	9.070223e‐01
Demersal fish	1.338207e+00	3.648652e+00	7.296833e+00
Planktivorous fish	1.387123e+00	6.343029e+00	3.368735e+00
Sediment porewater ammonia	1.584903e+00	2.063427e+00	2.510188e+00
Corpses	1.955949e+00	2.898967e+00	3.027624e+00
Carnivorous zooplankton	4.978142e+00	5.792072e+00	9.320204e+00
Surface layer phytoplankton	6.170512e+00	7.422738e+00	7.392445e+00
Deep layer detritus	6.348369e+00	6.665317e+00	8.912465e+00
Benthos carn/scav feeders	9.294691e+00	1.036397e+01	8.751406e+00
Surface layer detritus	1.061713e+01	1.126787e+01	1.137937e+01
Benthos susp/dep feeders	4.253288e+01	5.231259e+01	5.142915e+01
Omnivorous zooplankton	4.822393e+01	5.846732e+01	9.471605e+01
Macrophyte nitrogen	4.873217e+01	4.722330e+01	5.041410e+01
Surface layer ammonia	5.800559e+01	5.609282e+01	1.930920e+02
Deep layer ammonia	7.257979e+01	7.652691e+01	3.893234e+02
Surface layer nitrate	6.658904e+02	4.441402e+02	2.946609e+02
Deep layer nitrate	1.436946e+03	1.216641e+03	1.151911e+03
Sediment refractory detritus	1.592244e+04	1.592244e+04	1.766065e+04
Sediment labile plus refractory detritus	1.599796e+04	1.602212e+04	1.776644e+04

*Note:* Masses are presented for the whole model domain as annual averages in nitrogen, expressed per unit area (mmols N.y^−1^.m^−2^). The table is ordered with the lowest concentrations in the first rows (Heath et al. 2022).

Abbreviations: BS, Barents Sea model; GL = Northeast Greenland continental shelf model.

The maritime mammal guild is exceptional as the only consumer guild losing mass in the future. Maritime mammals depend on sea‐ice area for habitat in the model. Over our studied time period, sea ice cover transitions from perennial to seasonal. In the 2010s, the minimum ice affected area for the inshore and offshore zones was 59% and 55%, respectively. By the 2050s, these values fall to 0.002% and 0.006%.

Migratory fish mass is essentially unchanged into the future. This is because the stock of migratory fish is a fixed boundary condition for the model from which a proportion undertakes a seasonal migration into the model domain. Hence, migratory fish are only modelled for a portion of the year in the model domain and are included for their predatory and recycling effects on other parts of the food web.

The final masses achieved in the northeast Greenland model in the 2050s are comparable to the Barents Sea in the 2010s (Table 4). This appears sensible, as the Greenland model is transitioning into a seasonally ice‐free state more akin to the Barents Sea. One noticeable difference is a greater concentration of cetacean mass in the Barents Sea in the 2010s than in Greenland in the 2050s (0.4532786 mmolN.y^−1^.m^−2^ cf. 0.3137842 mmolN.y^−1^.m^−2^) and a greater concentration of pinnipeds (7.754153e‐2 mmolN.y^−1^.m^−2^ cf. 7.148973e‐2 mmolN.y^−1^.m^−2^) and maritime mammals (4.774963e‐3 mmolN.y^−1^.m^−2^ cf. 2.516999e‐3 mmolN.y^−1^.m^−2^) in Greenland. This is likely due to their still being more sea ice in the Greenlandic model, which provides habitat to maritime mammals and pinnipeds, at the expense of cetaceans.

### 3.3 Food Web Maturity

As the productivity of the system increases, the network's structure becomes more mature, as evidenced by the lengthening of the food web. The mean trophic level of top predators increases from 3.9 to 4.05 (Figure S2). Consumer diets become more specialised, with the mean omnivory index decreasing from 0.14 to 0.13. For seabirds, the omnivory index drops from 0.89 to 0.5 (Figure S3). Changes in mean trophic level will arise from changes in the balance of consumed guilds. For low trophic guilds, there is little variation in the trophic level of target guilds. Lower trophic guilds also have a lower number of prey guilds; for example, the two benthos guilds have 3 and 4 prey guilds, including dead matter, kelp, phytoplankton, and benthos. The top predators (excluding maritime mammals) meanwhile have between 8 and 10 prey guilds ranging from dead matter and zooplankton to planktivorous fish and pinnipeds. Changes to diet can therefore exert a larger effect on mean trophic level for guilds higher in the food web. In particular, the 357% increase in planktivorous fish mass satisfies a larger portion of the diet of cetaceans, pinnipeds, and seabirds, as this is the preferred food source for seals and birds, and a close second preference for cetaceans.

Considering the whole system, the dominance of indirect effects rises from 1.47 to 1.57, indicating a more connected food web. Internal capacity increases by 128.1% in line with higher levels of biological activity. However, the internal ascendancy to capacity ratio decreases slightly from 0.51 to 0.49.

### 3.4 Causal Inference

All model runs successfully reached steady state, with results available on PURE (Laverick et al. 2025b).

**FIGURE 4:**
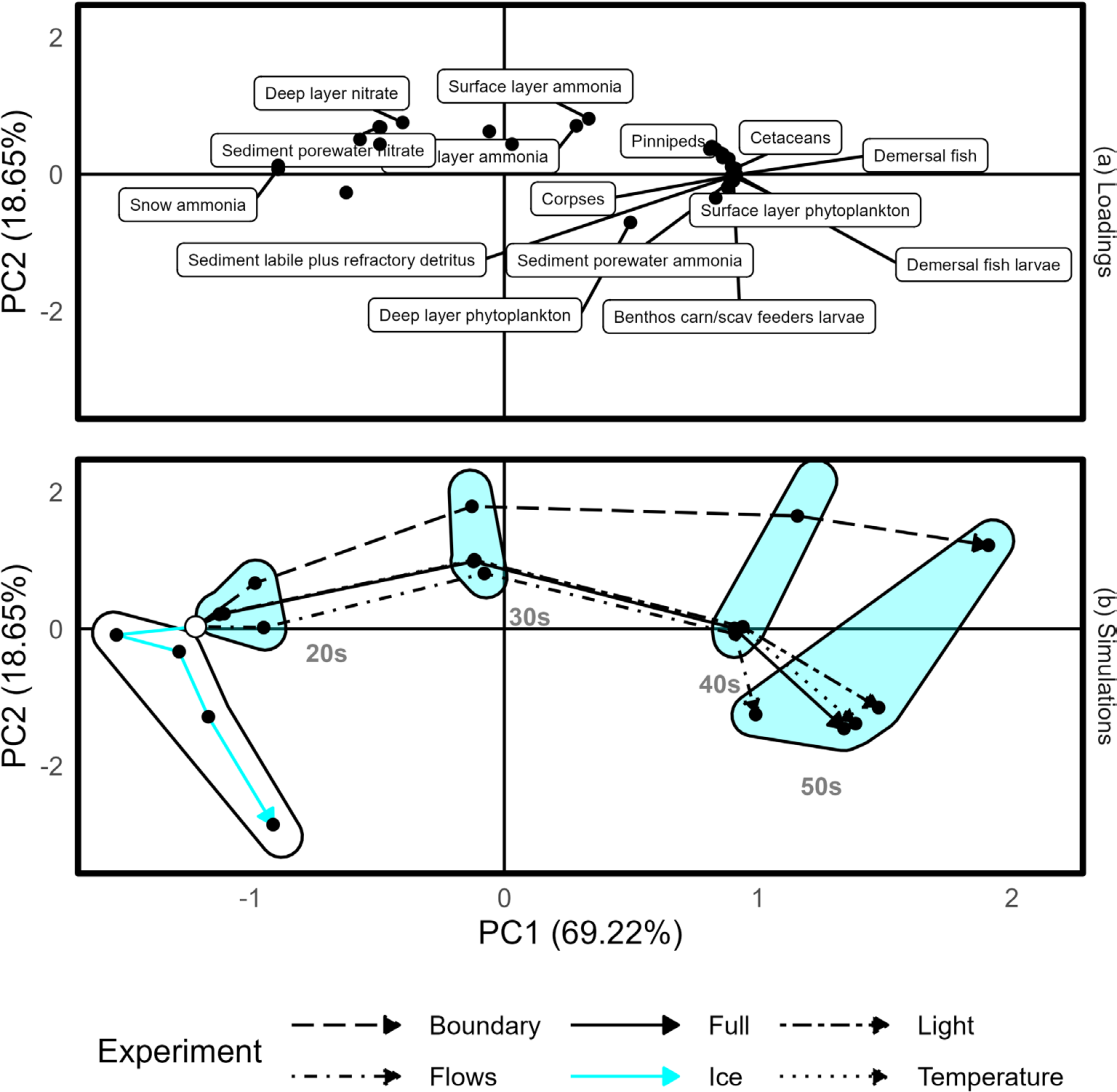
Principal Component Analysis of ecosystem state under a climate experiment. Axes are scaled in size to reflect the proportion of variation explained by the principal components. (a) shows the covariance between mass of a guild and the first two principal components of variation. The points should be read as vectors from the origin. All guilds are marked as points, but only the 10 and 5 guilds with the largest absolute covariance with PC1 and PC2 respectively are labelled with text. (b) shows model run steady states for each parameterisation described in Table 3 for all future decades, with the 2010s start conditions represented by a white dot and trajectories ending in an arrow. Blue hulls surround the decade used for climate forcing. A single white hull highlights all runs from the ice experiment. Lines connect the temporal trajectory between steady states.

Principal component one (PC1) in Figure 4 largely captures the effects of climate change over time. As time progresses, the total nutrient locked in the cryosphere reduces, and the mass in higher trophic levels increases (Figure S4), mirroring Figure 3. Principal component two (PC2) appears to capture water column nutrient concentrations (Figure S4), with a humped temporal pattern in Figure 4b suggesting that nutrient leaves the ice, enters the water column, and is later channelled to higher trophic levels.

The flows, temperature, and light experimental conditions largely track the full climate change scenario (Figure 4), implying these factors do little to drive the observed ecosystem trajectory over time. The boundary experiment is also broadly similar in trajectory, especially remembering that PC1 accounts for 69.22% of the variability in masses across all model runs. The differences for this experiment are captured on PC2 indicating elevated water column nutrient at all time steps, which is unsurprising as this experiment comprises changes to boundary nutrient concentrations. The ice experiment follows its own trajectory with little change in PC1 and increasingly negative values in PC2. This indicates changes in cryosphere forcing variables are necessary for the migration of ecosystem state towards increased productivity.

**FIGURE 5:**
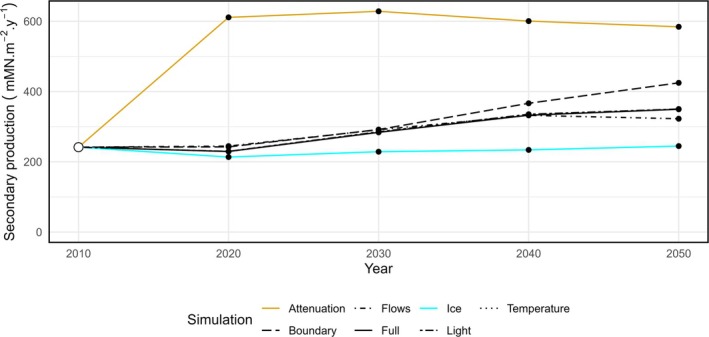
Changes in net production of all secondary and higher trophic levels under a climate experiment. Points are model run steady states for each parameterisation described in Table 3 for all future decades, with the 2010s start conditions represented by a white dot. An additional attenuation experiment is illustrated in yellow, where all conditions are the same as the Ice experiment, but light attenuation and reflection due to snow and ice are set to 0.

To determine whether the arresting influence of sea ice was due to limiting light or nutrient, we conducted a further daughter experiment. The ice experiment detailed in Table 3 was modified to set the light attenuation coefficients of light and snow and the reflection of sunlight to 0. This allowed light to pass into the water column but prevented the release of nutrient entrained in snow and ice. Note that this is equivalent to losing 100% of sea ice all year round. This additional experiment was not included in Figure 4 as the presence of sea ice with increased productivity led to higher mass in polar bears and fish, without the same scale of increase in cetacean mass or loss of ice‐bound nutrient. These patterns significantly altered the principal components in Figure 4 to accommodate the third ecosystem state, revealing only a single outlier trajectory. Instead, we plot total secondary production (Figure 5) for each of the climate experiments over time. The attenuation experiment indicates that secondary production is light, rather than nutrient, limited.

### 3.5 Sensitivity Analyses

**FIGURE 6:**
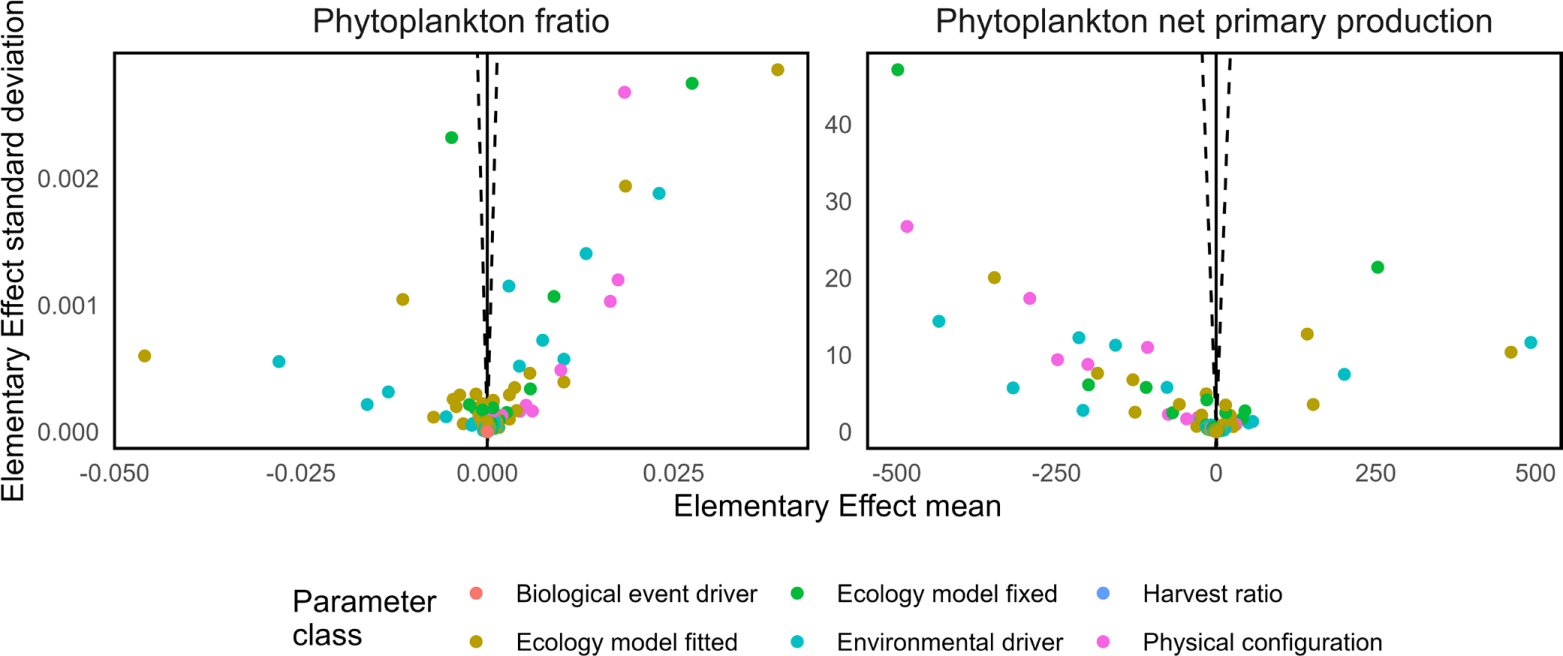
The sensitivity of the phytoplankton f‐ratio and net primary production. Parameters falling outside the dashed lines have a statistically significant effect. Values are available as a table in the Supporting Information (Figure S5). The colour coding indicates broad families of parameters. Biological event drivers include migration and reproductive dates. Fitted parameters in the ecology model include uptake and mortality parameters. Fixed parameters in the ecology model include Q10 reference temperatures. Environmental drivers are listed in Table 1. Harvest ratios relate to the fishing sub‐model and so have no effect after setting fishing activity to 0. The Physical configuration parameters include the thicknesses and areas of model compartments.

55.8% of parameters had a statistically significant effect on 2010s phytoplankton net primary production while this was true of 56.2% of parameters on the phytoplankton F‐ratio (Figure 6). Of these sensitive parameters, 185/246 (75.2%) and 188/248 (75.8%) were borrowed from the Barents Sea instead of being specifically sourced for the Northeast Greenland continental shelf implementation for phytoplankton net primary production and the F‐ratio respectively. The list of sensitive parameters for both metrics and their effects is available in the Supporting Information (Figure S5), along with a figure illustrating the distribution of all elementary effect sizes (Figure S6).

The 2010s phytoplankton F‐ratio is 0.95. The largest mean elementary effect for a borrowed parameter was −0.046 for the maximum uptake rate of ammonia by phytoplankton. The median effect across all parameters was 1.18e‐7.

The 2010s phytoplankton net primary production was −96.5 mmolN.m^−2^.year^−1^. The largest mean elementary effect for a borrowed parameter was −498.60 mmolN.m^−2^.year^−1^ for the saturation light intensity for the uptake of nutrient by phytoplankton. The median effect across all parameters was −0.0036 mmolN.m^−2^.year^−1^.

## 4 Discussion

### 4.1 The Modelled System Becomes More Productive

In our simulations, biological activity increased by nearly 40% under future climate conditions, as the attenuation of light by sea‐ice in the model domain reduced by a factor of 1.8. Field observations corroborate that melting sea‐ice increases the availability of sunlight in the water column (Castellani et al. 2022), supporting increased primary production in the Fram Strait (Castagno et al. 2023), but also across the Arctic Ocean (Arrigo and van Dijken 2015; Brandt et al. 2023). This drives a classical bottom‐up trophic cascade, with increases in consumer mass (Figure 3) and a reduction in the relative importance of recycling pathways in the food web (Figure 2). We have shown that it is the indirect effect of climate change melting sea‐ice that is responsible for this boost in productivity, rather than changes in temperatures or currents per se, through our climate experiments (Figure 4). Holding parameters such as sea‐ice thickness and concentration (Table 3) at the levels in the 2010s, while allowing climate projections to force increasing temperatures to the 2050s, did not result in an increase in productivity. However, allowing 100% of incoming irradiance to pass through sea‐ice to the water column resulted in increased consumer production (Figure 5).

As well as increasing biological activity, melting sea‐ice increases the accessibility of Arctic seas (Eguíluz et al. 2016; Ng et al. 2018). The adjacent North Atlantic contains heavily exploited fisheries (Merino et al. 2014) and it should be expected that parties will be interested in exploiting this new resource on the northeast Greenland continental shelf (Hoag 2017). Our simulations are therefore important to inform on the likely limits of production and how these may evolve over time. This information is necessary to avoid overexploitation of nascent fisheries, where fishing power may readily outstrip production, to a sub‐optimal state (Perissi et al. 2017).

### 4.2 The Food Web Becomes More Mature

Food webs are described as increasing in maturity as structural complexity and biological activity increase (Margalef 1968; Odum 1969; Pérez‐Espaa and Arreguín‐Sánchez 2001). In our simulations, mass is redistributed through the food web as productivity increases. Specifically, phytoplankton mass is channelled both directly into the omnivorous zooplankton guild as food, but also indirectly as detritus. The omnivorous zooplankton is then consumed by the planktivorous fish guild, which in turn directly feeds pinnipeds, sea birds, cetaceans, and demersal fish. Consumer guilds increase in mass by the most (Figure 2), with greater proportional increases as trophic level increases (Figure 3). Once again this is in keeping with broader theory (Heath et al. 2014; Libralato et al. 2014) as well as observations on the distribution of mass (Bar‐On et al. 2018; Burgess and Gaines 2018) and bottom‐up trophic cascades in other systems (Kagata and Ohgushi 2006).

Changes in the distribution of mass leads to changes in dietary patterns. We predict that future climate conditions will lengthen the food web as an increase in mean trophic level for top predators (3.9 to 4.05). This is as guilds at upper trophic levels find an increased availability of prey items from higher trophic levels. Top predators can satisfy a larger proportion of their diet from preferred food sources, leading to a reduction in the consumer omnivory index. This is particularly true of seabirds (reduction of 0.39), which are used as an indicator species for ecosystem health (Durant et al. 2009; Mallory et al. 2006).

In addition to the above indicators of ecosystem state, food web indices imply increased ecosystem resilience. The dominance of indirect effects on the system increases as the food web becomes more connected. Increased food web connectedness has been shown to positively impact robustness (Dunne et al. 2002; Yen et al. 2016). Meanwhile, food web ascendancy and capacity are measures of simultaneous growth and development, and the limit of growth and development (Ulanowicz 2000). Under climate change the modelled system shows an increase in capacity (128.1%) with relatively little change in the ascendancy to capacity ratio (−0.02). This is because changes are driven by increased biological activity while there is no change in the number of model compartments. These results suggest increases in biological activity are not at the expense of food web functional resilience in the face of climate change (Equihua et al. 2020). This is unsurprising as our model is comprised of functional groups, and the number of groups remained constant.

### 4.3 Polar Bear Mass Decreases Over Time

It is already well established that polar bears, maritime mammals in our model, depend on sea‐ice for their survival (Johnson et al. 2020). Though hibernation occurs on land, polar bears use the sea‐ice to hunt. Comparing the sea‐ice conditions between the 2010s and 2050s shows that in the future polar bears may have to meet their energetic requirements from hunting grounds which are seasonally about five orders of magnitude smaller (Figure 1). A reduction in hunting opportunities is not the only consequence of receding habitat. Melting sea‐ice has been implicated in increasing instances of human‐wildlife conflict (Smith et al. 2023) as well as intra‐specific competition. Though our model does not include the direct effects of polar bears encroaching on humans, it does include an interference factor reflecting competition. Our model implies, short of reversing sea‐ice loss, conservation interventions for polar bears could focus on securing sources of food (Palmer 2021). Whether adjustments to food availability in the absence of sea‐ice would benefit polar bears depends on their competitive ability on land (Miller et al. 2015).

### 4.4 Study Limitations

StrathE2EPolar is designed to be fast enough to run to steady states. This allows us to investigate the attractors of a system and describe the consequences of change independent of starting conditions. This is a strength of the model, as we do not require accurate surveys of initial values of state variables to generate meaningful results. However, will the ecosystem ever reach steady state in reality? The real world is changeable, allowing communities to persist despite being inherently unstable (Dial and Roughgarden 1998). Our results should therefore not be interpreted as accurate forecasts of population sizes in each decadal period, but rather for strategic insight (Evans et al. 2013) into how changes in key processes may cascade through the modelled system.

A second limitation of StrathE2EPolar is the reliance on guilds rather than species. Again, this affords speed and constraints on parameter numbers, but would we expect the taxonomic makeup of a guild to remain the same in the future? Studies are suggesting that species may track thermal envelopes under climate change and migrate towards the poles (Brandt et al. 2023). This will result in the loss of endemic biodiversity, which may be replaced by temperate species, for example, a loss of Narwhals but a gain in Humpback whales (Heide‐Jørgensen et al. 2023). What remains unclear without a species‐level analysis is the rate at which this process may occur, and the consequences for ecosystem function (Kortsch et al. 2015). In the absence of a theory of change, we have left the physiological parameters for guilds, which reflect their species makeup, unchanged in our climate simulations. These parameters include prey half‐saturation coefficients and maximum uptake rates, prey preferences, and density‐dependent mortality coefficients; the rates of change in our model are an emergent property of the system.

As well as a potential turnover in guild composition, increased primary production may attract increased levels of migratory fish to the northeast Greenland continental shelf. The impact of changes in migratory fish will depend on their prey preferences, which may also change in the future. Assuming the prey preferences remain unchanged in our model, increased migratory fish biomass would result in more of a highly preferred food source for cetaceans and a low preference food source for seabirds and demersal fish. Meanwhile, the migratory fish would exert increased predation pressure on omnivorous zooplankton and benthos.

We conducted full sensitivity analyses (Morris et al. 2014; Morris 1991) of the Northeast Greenland continental shelf model for phytoplankton net primary production and F‐ratio (Eppley and Peterson 1979). These metrics were chosen as they are key to our finding of an increasingly productive system under climate change. These analyses allowed us to quantify the significance of borrowing parameters from the Barents Sea, as well as the robustness of our results more generally.

Just over half of all parameters exert a statistically significant effect on net primary production and the phytoplankton F‐ratio. Of these, about three quarters were borrowed from the Barents Sea, representing a minority of the total set of parameters. Inspecting the table of mean elementary effects (Figure S5) reveals that the parameters with the largest effects were, unsurprisingly, those related to phytoplankton physiology and the uptake of nutrients (maximum uptakes rates of ammonia and nitrate as well as the saturation light intensity for the uptake of nutrient). We suggest that physiological characteristics such as these are unlikely to differ markedly between two geographically proximate and ecologically similar shelf seas. We therefore have confidence in the results of our simulation for the northeast Greenland continental shelf.

Though we lack field observations to compare to the results of our model for confidence, we can compare the per unit area concentrations of nitrogen mass to our Barents Sea model (Table 4). Firstly, this is advantageous as we can make direct comparison to results derived from the same model framework. An issue with comparing to fragments of field observations is that they may be incomplete in space, time, and taxa. Meanwhile, the two different implementations of StrathE2E*polar* are bound by the same requirement to conserve mass. Secondly, the Barents Sea is a more intensively studied system. This means we were able to fit the Barents Sea model to a myriad of field data. The comparison in Table 4 is therefore an indirect comparison of our new model to observations from another arctic ecosystem. We are reassured that the concentrations reported here for the Northeast Greenland Continental Shelf model in a seasonally ice‐free future are in keeping with observations from an analogous system in the present day.

Our model results provide a baseline against which we can assess the coming changes to the ecosystem of the Greenlandic northeast continental shelf. Models are powerful when used as part of an iterative process of simulation and data collection to further improve our simulations. As this region is data poor, we have three recommendations for future data collection activities. Firstly, our sensitivity analyses indicate that a characterization of the nutrient uptake rates by phytoplankton communities of the Northeast Greenland shelf under different conditions would help reduce uncertainties in primary production. Secondly, estimates of top predator and fish guild biomass on a per unit area basis would allow us to constrain density‐dependent parameters within our model. Finally, data on the diet composition of consumer guilds would allow us to constrain feeding preferences and thus the effective connectance of guilds in the food web.

It is important to remember our findings are limited to the northeast Greenland continental shelf. Any suggestions of increased productivity and more mass at higher trophic levels under climate change should not be used to characterise the global impacts of climate change. The borealisation of the Arctic (Polyakov et al. 2020) occurs alongside the emergence of novel thermal envelopes forcing taxa to migrate poleward. As well as losing typical Arctic habitats (Chambault et al. 2022), novel equatorial conditions may reduce primary production (Lu et al. 2024).

## Author Contributions

**Jack H. Laverick:** conceptualization, data curation, formal analysis, investigation, methodology, visualization, writing – original draft. **Douglas C. Speirs:** conceptualization, funding acquisition, project administration, resources, supervision, writing – review and editing. **Michael R. Heath:** conceptualization, data curation, funding acquisition, project administration, resources, software, supervision, validation, writing – review and editing.

## Conflicts of Interest

The authors declare no conflicts of interest.

## Supporting information


Figures S1–S6.

Data S1.

## Data Availability

The data, code, and models that support the findings of this study are openly available in PURE at https://pureportal.strath.ac.uk/en/datasets/codebase‐for‐the‐strathe2epolar‐parameterisation‐of‐the‐northeast, https://pureportal.strath.ac.uk/en/datasets/an‐implementation‐of‐strathe2epolar‐for‐the‐northeast‐greenland‐c, https://pureportal.strath.ac.uk/en/datasets/simulated‐results‐for‐sea‐ice‐retreat‐from‐the‐northeast‐greenla.

## References

[gcb70189-bib-0001] Arrigo, K. R. , and G. L. van Dijken . 2015. “Continued Increases in Arctic Ocean Primary Production.” Progress in Oceanography 136: 60–70. 10.1016/J.POCEAN.2015.05.002.

[gcb70189-bib-0002] Bacon, S. , A. Marshall , N. P. Holliday , Y. Aksenov , and S. R. Dye . 2014. “Seasonal Variability of the East Greenland Coastal Current.” Journal of Geophysical Research: Oceans 119, no. 6: 3967–3987. 10.1002/2013JC009279.

[gcb70189-bib-0003] Bar‐On, Y. M. , R. Phillips , and R. Milo . 2018. “The Biomass Distribution on Earth.” Proceedings of the National Academy of Sciences of the United States of America 115, no. 25: 6506–6511. 10.1073/PNAS.1711842115. 29784790 PMC6016768

[gcb70189-bib-0004] Brandt, S. , P. Wassmann , and D. Piepenburg . 2023. “Revisiting the Footprints of Climate Change in Arctic Marine Food Webs: An Assessment of Knowledge Gained Since 2010.” Frontiers in Marine Science 10: 1096222. 10.3389/FMARS.2023.1096222.

[gcb70189-bib-0005] Burgess, M. G. , and S. D. Gaines . 2018. “The Scale of Life and Its Lessons for Humanity.” Proceedings of the National Academy of Sciences of the United States of America 115: 6328–6330. 10.1073/pnas.1807019115. 29793935 PMC6016823

[gcb70189-bib-0006] Carvalho, K. S. , and S. Wang . 2020. “Sea Surface Temperature Variability in the Arctic Ocean and Its Marginal Seas in a Changing Climate: Patterns and Mechanisms.” Global and Planetary Change 193: 103265. 10.1016/J.GLOPLACHA.2020.103265.

[gcb70189-bib-0007] Castagno, A. P. , T. J. W. Wagner , M. R. Cape , et al. 2023. “Increased Sea Ice Melt as a Driver of Enhanced Arctic Phytoplankton Blooming.” Global Change Biology 29, no. 17: 5087–5098. 10.1111/GCB.16815. 37332145

[gcb70189-bib-0008] Castellani, G. , G. Veyssiè , M. Karcher , et al. 2022. “Shine a Light: Under‐Ice Light and Its Ecological Implications in a Changing Arctic Ocean.” Ambio 51: 307–317. 10.1007/s13280-021-01662-3. 34822117 PMC8692516

[gcb70189-bib-0009] Chambault, P. , K. M. Kovacs , C. Lydersen , et al. 2022. “Future Seasonal Changes in Habitat for Arctic Whales During Predicted Ocean Warming.” Science Advances 8, no. 29: 2422. 10.1126/SCIADV.ABN2422. PMC9307241 35867786

[gcb70189-bib-0010] Clem, K. R. , R. L. Fogt , J. Turner , et al. 2020. “Record Warming at the South Pole During the Past Three Decades.” Nature Climate Change 10, no. 8: 762–770. 10.1038/s41558-020-0815-z.

[gcb70189-bib-0011] Dial, R. , and J. Roughgarden . 1998. “Theory of Marine Communities: The Intermediate Disturbance Hypothesis.” Ecology 79, no. 4: 1412–1424. 10.1890/0012-9658(1998)079[1412:TOMCTI]2.0.CO;2.

[gcb70189-bib-0012] Dunne, J. A. , R. J. Williams , and N. D. Martinez . 2002. “Network Structure and Biodiversity Loss in Food Webs: Robustness Increases With Connectance.” Ecology Letters 5: 558–567. 10.1046/j.1461-0248.2002.00354.x.

[gcb70189-bib-0013] Durant, J. M. , D. Ø. Hjermann , M. Frederiksen , et al. 2009. “Pros and Cons of Using Seabirds as Ecological Indicators.” Climate Research 39, no. 2: 115–129. 10.2307/24870429.

[gcb70189-bib-0014] Eguíluz, V. M. , J. Fernández‐Gracia , X. Irigoien , and C. M. Duarte . 2016. “A Quantitative Assessment of Arctic Shipping in 2010‐2014.” Scientific Reports 6, no. 1: 1–6. 10.1038/srep30682. 27477878 PMC4967913

[gcb70189-bib-0015] Eppley, R. W. , and B. J. Peterson . 1979. “Particulate Organic Matter Flux and Planktonic New Production in the Deep Ocean.” Nature 282, no. 5740: 677–680. 10.1038/282677a0.

[gcb70189-bib-0016] Equihua, M. , M. E. Aldama , C. Gershenson , et al. 2020. “Ecosystem Antifragility: Beyond Integrity and Resilience.” PeerJ 8: e8533. 10.7717/PEERJ.8533. 32095358 PMC7020813

[gcb70189-bib-0017] Evans, M. R. , V. Grimm , K. Johst , et al. 2013. “Do Simple Models Lead to Generality in Ecology?” Trends in Ecology & Evolution 28, no. 10: 578–583. 10.1016/j.tree.2013.05.022. 23827437

[gcb70189-bib-0018] Hansen, A. S. , T. G. Nielsen , H. Levinsen , S. D. Madsen , T. F. Thingstad , and B. W. Hansen . 2003. “Impact of Changing Ice Cover on Pelagic Productivity and Food Web Structure in Disko Bay, West Greenland: A Dynamic Model Approach.” Deep Sea Research Part I: Oceanographic Research Papers 50, no. 1: 171–187. 10.1016/S0967-0637(02)00133-4.

[gcb70189-bib-0019] Heath, M. R. , D. Benkort , A. S. Brierley , et al. 2022. “Ecosystem Approach to Harvesting in the Arctic: Walking the Tightrope Between Exploitation and Conservation in the Barents Sea.” Ambio 51: 456–470. 10.1007/s13280. 34478036 PMC8692644

[gcb70189-bib-0020] Heath, M. R. , D. C. Speirs , and J. H. Steele . 2014. “Understanding Patterns and Processes in Models of Trophic Cascades.” Ecology Letters 17, no. 1: 101–114. 10.1111/ele.12200. 24165353 PMC4237542

[gcb70189-bib-0021] Heath, M. R. , D. C. Speirs , I. Thurlbeck , and R. J. Wilson . 2021. “StrathE2E2: An R Package for Modelling the Dynamics of Marine Food Webs and Fisheries.” Methods in Ecology and Evolution 12, no. 2: 280–287. 10.1111/2041-210X.13510.

[gcb70189-bib-0022] Heide‐Jørgensen, M. P. , P. Chambault , T. Jansen , et al. 2023. “A Regime Shift in the Southeast Greenland Marine Ecosystem.” Global Change Biology 29, no. 3: 668–685. 10.1111/GCB.16494. 36408667 PMC10099497

[gcb70189-bib-0023] Heymans, J. J. , M. Coll , J. S. Link , et al. 2016. “Best Practice in Ecopath With Ecosim Food‐Web Models for Ecosystem‐Based Management.” Ecological Modelling 331: 173–184. 10.1016/J.ECOLMODEL.2015.12.007.

[gcb70189-bib-0024] Hoag, H. 2017. “Nations Agree to Ban Fishing in Arctic Ocean for at Least 16 Years.” Science. 10.1126/science.aar6437.

[gcb70189-bib-0025] Hofer, S. , C. Lang , C. Amory , et al. 2020. “Greater Greenland Ice Sheet Contribution to Global Sea Level Rise in CMIP6.” Nature Communications 11, no. 1: 6289. 10.1038/s41467-020-20011-8. PMC7738669 33323939

[gcb70189-bib-0026] Holland, M. M. , and C. M. Bitz . 2003. “Polar Amplification of Climate Change in Coupled Models.” Climate Dynamics 21, no. 3–4: 221–232. 10.1007/s00382-003-0332-6.

[gcb70189-bib-0027] ICES . 2020. “Working Group on Integrated Ecosystem Assessment of the Greenland Sea (WGIEAGS).” ICES Scientific Reports. 2(75).

[gcb70189-bib-0028] IPCC . 2019. “Summary for Policymakers.” In IPCC Special Report on the Ocean and Cryosphere in a Changing Climate, edited by H.‐O. Pörtner , D. C. Roberts , V. Masson‐Delmotte , et al. IPCC.

[gcb70189-bib-0029] Johnson, A. C. , J. R. Reimer , N. J. Lunn , I. Stirling , D. Mcgeachy , and A. E. Derocher . 2020. “Influence of Sea Ice Dynamics on Population Energetics of Western Hudson Bay Polar Bears.” Conservation Physiology 8, no. 1: coaa132. 10.1093/conphys/coaa132. 33408870 PMC7772618

[gcb70189-bib-0030] Kagata, H. , and T. Ohgushi . 2006. “Bottom‐Up Trophic Cascades and Material Transfer in Terrestrial Food Webs.” Ecological Research 21, no. 1: 26–34. 10.1007/s11284-005-0124-z.

[gcb70189-bib-0031] Keramidas, I. , D. Dimarchopoulou , E. Ofir , M. Scotti , A. C. Tsikliras , and G. Gal . 2023. “Ecotrophic Perspective in Fisheries Management: A Review of Ecopath With Ecosim Models in European Marine Ecosystems.” Frontiers in Marine Science 10: 1182921. 10.3389/FMARS.2023.1182921.

[gcb70189-bib-0032] Koenigk, T. , J. Key , T. Vihma , J. Key , and T. Vihma . 2020. “Climate Change in the Arctic.” In Physics and Chemistry of the Arctic Atmosphere, 673–705. Springer. 10.1007/978-3-030-33566-3_11.

[gcb70189-bib-0033] Kones, J. , K. Soetaert , D. van Oevelen , and J. Owino . 2009. “Are Network Indices Robust Indicators of Food Web Functioning? A Monte Carlo Approach.” Ecological Modelling 220, no. 3: 370–382. 10.1016/j.ecolmodel.2008.10.012.

[gcb70189-bib-0034] Kortsch, S. , R. Primicerio , M. Aschan , S. Lind , A. V. Dolgov , and B. Planque . 2019. “Food‐Web Structure Varies Along Environmental Gradients in a High‐Latitude Marine Ecosystem.” Ecography 42, no. 2: 295–308. 10.1111/ECOG.03443.

[gcb70189-bib-0035] Kortsch, S. , R. Primicerio , M. Fossheim , A. V. Dolgov , and M. Aschan . 2015. “Climate Change Alters the Structure of Arctic Marine Food Webs due to Poleward Shifts of Boreal Generalists.” Proceedings of the Royal Society B: Biological Sciences 282, no. 1814: 20151546. 10.1098/RSPB.2015.1546. PMC4571709 26336179

[gcb70189-bib-0036] Kwok, R. 2018. “Arctic Sea Ice Thickness, Volume, and Multiyear Ice Coverage: Losses and Coupled Variability (1958–2018).” Environmental Research Letters 13, no. 10: 105005. 10.1088/1748-9326/AAE3EC.

[gcb70189-bib-0037] Laverick, J. 2025. “Codebase for the StrathE2Epolar Parameterisation of the Northeast Greenland Continental Shelf.” In PURE. 10.15129/d48d9bfb-336f-4e11-8a0b-b633e665d61a.

[gcb70189-bib-0038] Laverick, J. , D. Speirs , and M. Heath . 2025a. “An Implementation of StrathE2EPolar for the Northeast Greenland Continental Shelf.” In PURE. 10.15129/2196ddee-47fe-4d01-8a87-ee61ec023ac5. PMC12019585 40270291

[gcb70189-bib-0039] Laverick, J. , D. Speirs , and M. Heath . 2025b. “Simulated Results for: “Sea‐Ice Retreat From the Northeast Greenland Continental Shelf Triggers a Marine Trophic Cascade.”” In PURE. 10.15129/12d754a4-714f-4dd2-ae99-d3008cd1e362. PMC12019585 40270291

[gcb70189-bib-0040] Laverick, J. H. , D. C. Speirs , and M. R. Heath . 2023. “Synthetic Shelf Sediment Maps for the Greenland Sea and Barents Sea.” Geoscience Data Journal 10, no. 2: 220–230. 10.1002/GDJ3.154.

[gcb70189-bib-0041] Libralato, S. , F. Pranovi , K. I. Stergiou , and J. S. Link . 2014. “Trophodynamics in Marine Ecology: 70 Years After Lindeman.” Marine Ecology Progress Series 512: 1–7. 10.3354/meps11033.

[gcb70189-bib-0042] Lu, Q. , H. Liu , L. Wei , Y. Zhong , and Z. Zhou . 2024. “Global Prediction of Gross Primary Productivity Under Future Climate Change.” Science of the Total Environment 912: 169239. 10.1016/J.SCITOTENV.2023.169239. 38072275

[gcb70189-bib-0043] Mallory, M. L. , H. Grant Gilchrist , B. M. Braune , and A. J. Gaston . 2006. “Marine Birds as Indicators of Arctic Marine Ecosystem Health: Linking the Northern Ecosystem Initiative to Long‐Term Studies.” Environmental Monitoring and Assessment 113: 31–48. 10.1007/s10661-005-9095-3. 16514485

[gcb70189-bib-0044] Margalef, R. 1968. Perspectives in Ecological Theory. University of Chicago Press.

[gcb70189-bib-0045] McMeans, B. C. , N. Rooney , M. T. Arts , and A. T. Fisk . 2013. “Food Web Structure of a Coastal Arctic Marine Ecosystem and Implications for Stability.” Marine Ecology Progress Series 482: 17–28. 10.3354/MEPS10278.

[gcb70189-bib-0046] Merino, G. , M. Barange , J. A. Fernandes , et al. 2014. “Estimating the Economic Loss of Recent North Atlantic Fisheries Management.” Progress in Oceanography 129: 314–323. 10.1016/j.pocean.2014.04.022.

[gcb70189-bib-0047] Miller, S. , J. Wilder , and R. R. Wilson . 2015. “Polar Bear–Grizzly Bear Interactions During the Autumn Open‐Water Period in Alaska.” Journal of Mammalogy 96, no. 6: 1317–1325. 10.1093/JMAMMAL/GYV140.

[gcb70189-bib-0048] Morris, D. J. , D. C. Speirs , A. I. Cameron , and M. R. Heath . 2014. “Global Sensitivity Analysis of an End‐To‐End Marine Ecosystem Model of the North Sea: Factors Affecting the Biomass of Fish and Benthos.” Ecological Modelling 273: 251–263. 10.1016/j.ecolmodel.2013.11.019.

[gcb70189-bib-0049] Morris, M. D. 1991. “Factorial Sampling Plans for Preliminary Computational Experiments.” Technometrics 33, no. 2: 161. 10.2307/1269043.

[gcb70189-bib-0050] Ng, A. K. Y. , J. Andrews , D. Babb , Y. Lin , and A. Becker . 2018. “Implications of Climate Change for Shipping: Opening the Arctic Seas.” Wiley Interdisciplinary Reviews: Climate Change 9, no. 2: e507. 10.1002/WCC.507.

[gcb70189-bib-0051] Odum, E. P. 1969. “The Strategy of Ecosystem Development.” Science 164, no. 3877: 262–270. 10.1126/SCIENCE.164.3877.262. 5776636

[gcb70189-bib-0052] Oksanen, J. , G. L. Simpson , F. G. Blanchet , et al. 2022. “vegan: Community Ecology Package.” https://CRAN.R‐project.org/package=vegan.

[gcb70189-bib-0053] Palmer, C. 2021. Should We Provide the Bear Necessities? Climate Change, Polar Bears and the Ethics of Supplemental Feeding. Vol. 33, 377–398. International Library of Environmental, Agricultural and Food Ethics. 10.1007/978-3-030-63523-7_21/COVER.

[gcb70189-bib-0054] Pérez‐Espaa, H. , and F. Arreguín‐Sánchez . 2001. “An Inverse Relationship Between Stability and Maturity in Models of Aquatic Ecosystems.” Ecological Modelling 145, no. 2–3: 189–196. 10.1016/S0304-3800(01)00390-8.

[gcb70189-bib-0055] Perissi, I. , U. Bardi , T. El Asmar , and A. Lavacchi . 2017. “Dynamic Patterns of Overexploitation in Fisheries.” Ecological Modelling 359: 285–292. 10.1016/j.ecolmodel.2017.06.009. 28900312 PMC5569600

[gcb70189-bib-0056] Plagányi, É. E. , and D. S. Butterworth . 2004. “A Critical Look at the Potential of Ecopath With Ecosim to Assist in Practical Fisheries Management.” African Journal of Marine Science 26: 261–287. 10.2989/18142320409504061.

[gcb70189-bib-0057] Polyakov, I. V. , M. B. Alkire , B. A. Bluhm , et al. 2020. “Borealization of the Arctic Ocean in Response to Anomalous Advection From Sub‐Arctic Seas.” Frontiers in Marine Science 7: 516272. 10.3389/FMARS.2020.00491.

[gcb70189-bib-0058] R Core Team . 2019. “R: A Language and Environment for Statistical Computing.” R Foundation for Statistical Computing. http://www.r‐project.org/.

[gcb70189-bib-0059] Sévellec, F. , A. V. Fedorov , and W. Liu . 2017. “Arctic Sea‐Ice Decline Weakens the Atlantic Meridional Overturning Circulation.” Nature Climate Change 7, no. 8: 604–610. 10.1038/nclimate3353.

[gcb70189-bib-0060] Shu, Q. , F. Qiao , Z. Song , J. Zhao , and X. Li . 2018. “Projected Freshening of the Arctic Ocean in the 21st Century.” Journal of Geophysical Research: Oceans 123, no. 12: 9232–9244. 10.1029/2018JC014036.

[gcb70189-bib-0061] Smith, T. S. , A. E. Derocher , R. L. Mazur , et al. 2023. “Anthropogenic Food: An Emerging Threat to Polar Bears.” Oryx 57, no. 4: 425–434. 10.1017/S0030605322000278.

[gcb70189-bib-0062] Thackeray, C. W. , and A. Hall . 2019. “An Emergent Constraint on Future Arctic Sea‐Ice Albedo Feedback.” Nature Climate Change 9, no. 12: 972–978. 10.1038/s41558-019-0619-1.

[gcb70189-bib-0063] Tovar‐Sánchez, A. , C. M. Duarte , J. C. Alonso , S. Lacorte , R. Tauler , and C. Galban‐Malagón . 2010. “Impacts of Metals and Nutrients Released From Melting Multiyear Arctic Sea Ice.” Journal of Geophysical Research: Oceans 115, no. C7: 7003. 10.1029/2009JC005685.

[gcb70189-bib-0064] Troell, M. , A. Eide , J. Isaksen , Ø. Hermansen , and A. S. Crépin . 2017. “Seafood From a Changing Arctic.” Ambio 46, no. 3: 368–386. 10.1007/s13280-017-0954-2. 29080009 PMC5673870

[gcb70189-bib-0065] Ulanowicz, R. E. 2000. Growth and Development: Ecosystems Phenomenology. ToExcel Press.

[gcb70189-bib-0066] Wadham, J. L. , J. Hawkings , J. Telling , et al. 2016. “Sources, Cycling and Export of Nitrogen on the Greenland Ice Sheet.” Biogeosciences 13, no. 22: 6339–6352. 10.5194/BG-13-6339-2016.

[gcb70189-bib-0067] Wu, J. , R. Dhingra , M. Gambhir , and J. V. Remais . 2013. “Sensitivity Analysis of Infectious Disease Models: Methods, Advances and Their Application.” Journal of the Royal Society Interface 10, no. 86: 20121018. 10.1098/RSIF.2012.1018. 23864497 PMC3730677

[gcb70189-bib-0068] Yang, M. , Y. Qiu , L. Huang , et al. 2023. “Changes in Sea Surface Temperature and Sea Ice Concentration in the Arctic Ocean Over the Past Two Decades.” Remote Sensing 15: 1095. 10.3390/RS15041095.

[gcb70189-bib-0069] Yen, J. D. L. , R. B. Cabral , M. Cantor , et al. 2016. “Linking Structure and Function in Food Webs: Maximization of Different Ecological Functions Generates Distinct Food Web Structures.” Journal of Animal Ecology 85, no. 2: 537–547. 10.1111/1365-2656.12484. 26749320

[gcb70189-bib-0070] Yool, A. , E. E. Popova , and T. R. Anderson . 2013. “MEDUSA‐2.0: An Intermediate Complexity Biogeochemical Model of the Marine Carbon Cycle for Climate Change and Ocean Acidification Studies.” Geoscientific Model Development 6: 1767–1811.

[gcb70189-bib-0071] Yool, A. , E. E. Popova , and A. C. Coward . 2015. “Future Change in Ocean Productivity: Is the Arctic the New Atlantic.” Journal of Geophysical Research: Oceans 120, no. 12: 7771–7790. 10.1002/2015JC011167.

