# Supplementary material for: Sea‐Ice Retreat From the Northeast Greenland Continental Shelf Triggers a Marine Trophic Cascade
Source: Glob Chang Biol. 2025 Apr 24;31(4):e70189. doi: 10.1111/gcb.70189 (PMC12019585; doi:10.1111/gcb.70189)
Supplement: Supplementary file 1 — Figures S1–S6. [file GCB-31-e70189-s001.docx]

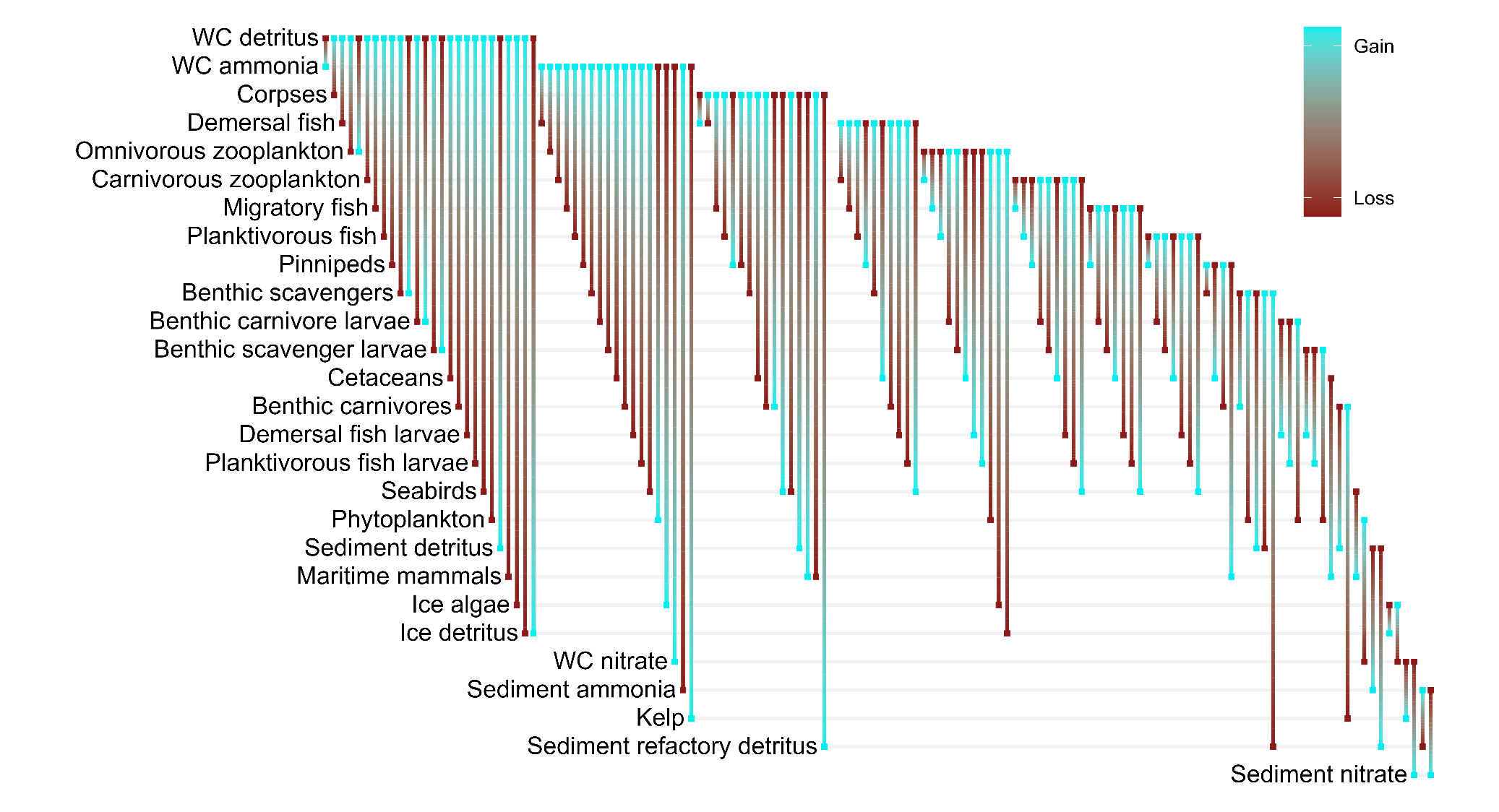


**SIFig 1: The StrathE2EPolar food web.** Guilds are listed down the left-hand side, with the most connected at the top and least connected at the bottom. Lines indicate a linkage between two guilds, read at the termini of the line. Reading across a row allows you to find all the links that guild has to other parts of the food web. Blues indicate the guild which is gaining from the link (a consumer), reds indicate the guild losing nitrogen mass through the link (a source or producer). Links to the model boundary conditions, such as open ocean or terrestrial sources of nitrogen, have been omitted. WC is an abbreviation of Water Column.


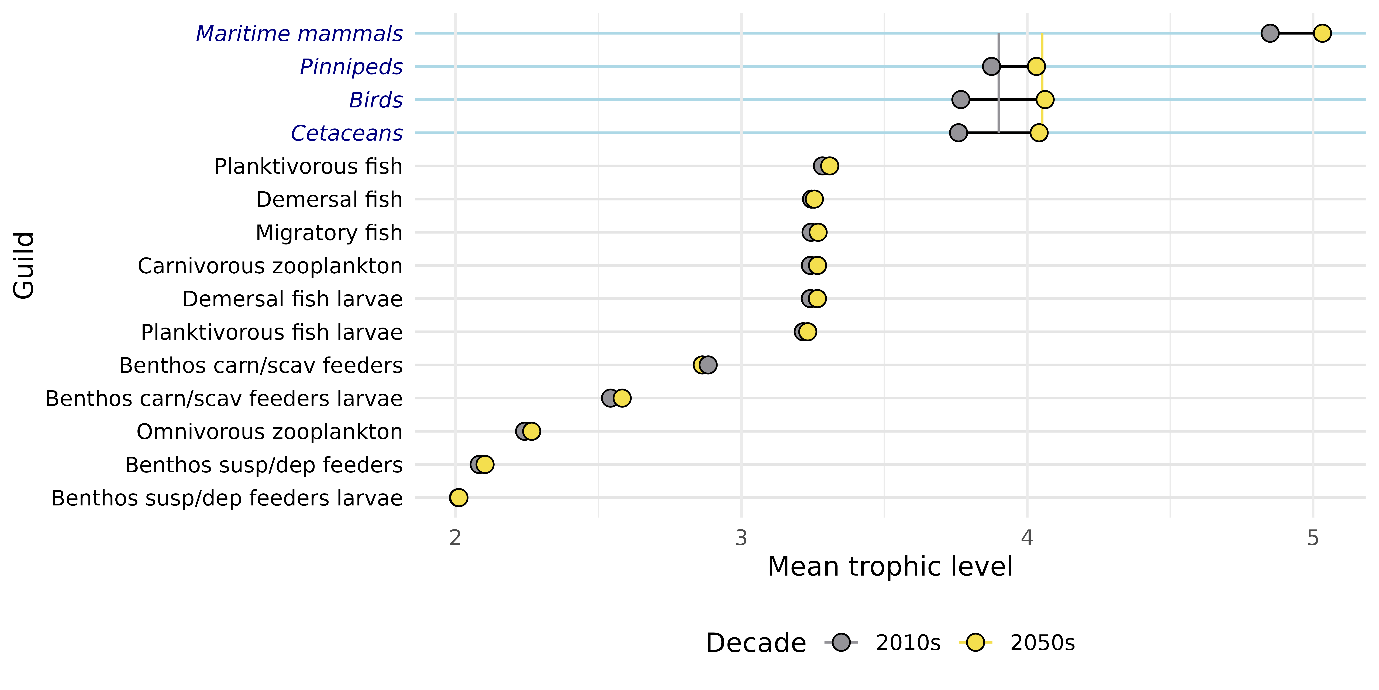


**SIFig 2: Change in mean trophic level under climate change for consumer guilds.** Top predators are marked with blue axis labels and grid lines. The Vertical grey and yellow lines mark the biomass-weighted mean trophic level across top predator guilds. Changes in trophic level are most pronounced at the top of the food web.


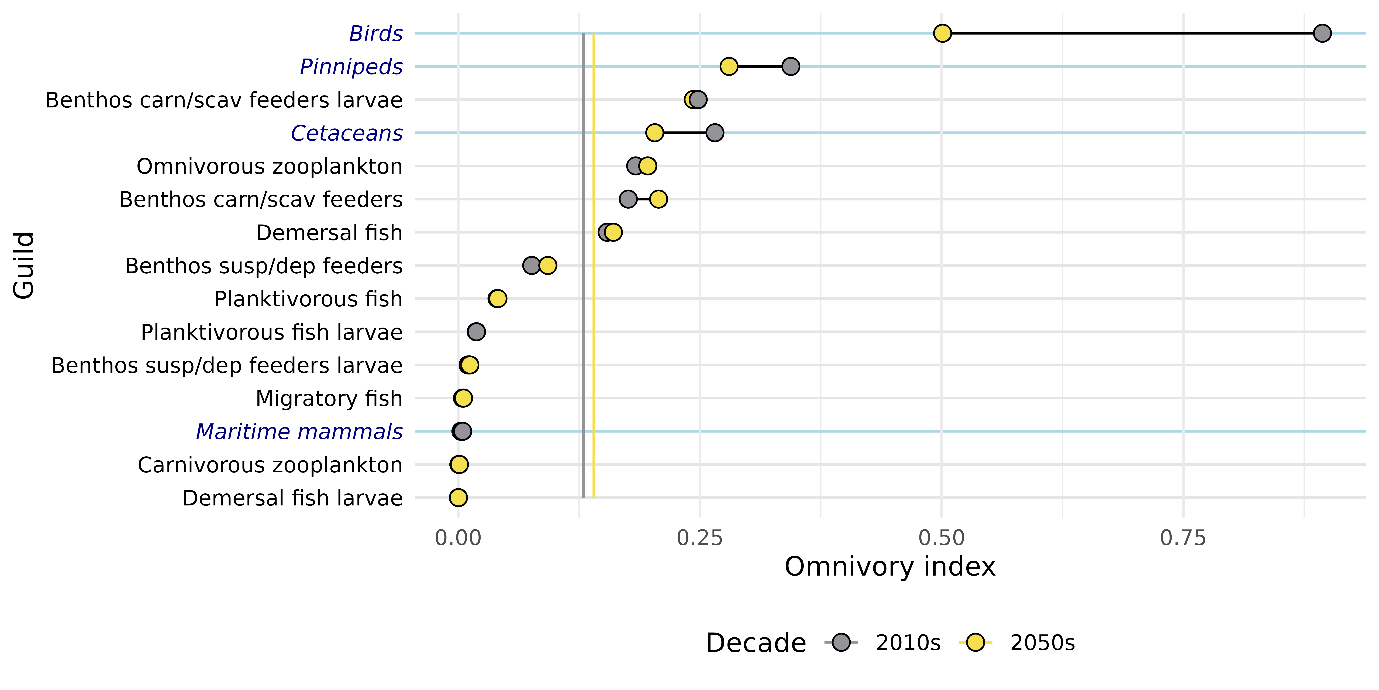


**SIFig 3: Change in Omnivory index under climate change for consumer guilds.** Top predators are marked with blue axis labels and grid lines. The Vertical grey and yellow lines mark the biomass-weighted mean omnivory index across all consumer guilds. A reduction in the omnivory index indicates a more specialised diet.


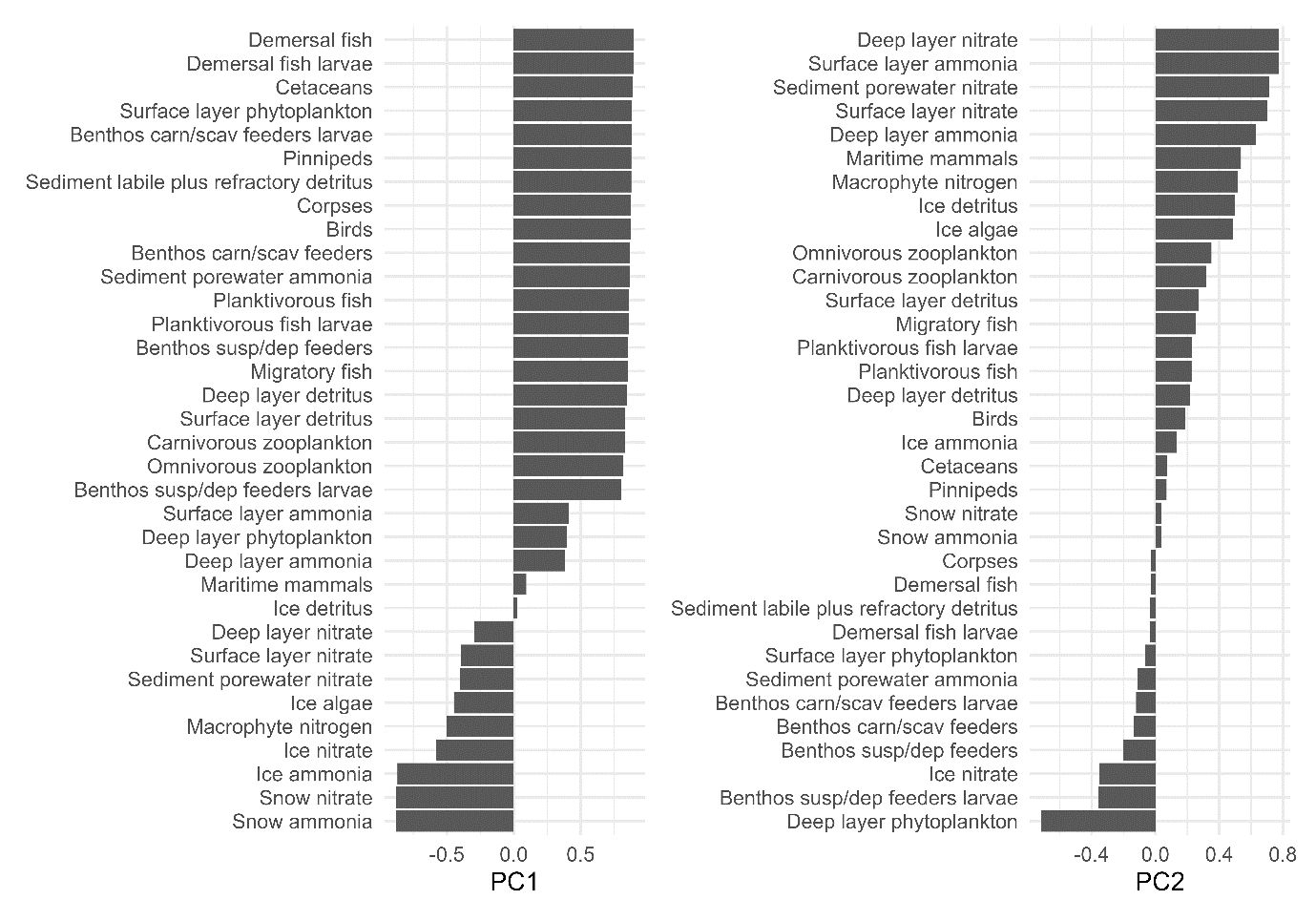


**SIFig 4: Loadings for each guild on the two principal components of Figure 4.** Loadings range from -1 to 1. Values near 0 mean there is little to no information encoded in a principal component for a particular guild.

**SIFig 5: Full sensitivity analysis results.** Parameter and ID indicate the value changed within StrathE2E. EE Mean is the mean elementary effect of changing a parameter under the “one at a time” method. EE sd is the standard deviation of the mean elementary effect. The effects are grouped by the metric assessed for sensitivity to changes in parameters (Phtyoplankton f-ratio or phytoplankton net primary production), and whether the parameter values were sourced from the Barent Sea of the East Greenland Shelf directly. Effects are listed in order of decreasing absolute magnitude within groups. Only effects statistically different from 0 are reported.

| Parameter | ID | EE Mean (3sf) | EE sd (3sf) | Description | Guild or feature |
| --- | --- | --- | --- | --- | --- |
| Phytoplankton fratio - Borrowed from Barents Sea | | | | | |
| uAMM_phytt | 335 | -4.60e-02 | 5.99e-04 | Maximum uptake rate | Ammonia by phytoplankton |
| hsAMM_phyt | 336 | 3.90e-02 | 2.86e-03 | Uptake half saturation coefficient | Ammonia by phytoplankton |
| Lmaxup_phyt | 316 | 2.75e-02 | 2.75e-03 | Saturation light intensity for uptake | Nutrient by phytoplankton |
| uNIT_phytt | 333 | 1.85e-02 | 1.94e-03 | Maximum uptake rate | Nitrate by phytoplankton |
| udet_omnit | 343 | -1.13e-02 | 1.04e-03 | Maximum uptake rate | Suspended detritus by omnivorous zooplankton |
| hsdet_omni | 344 | 1.03e-02 | 3.93e-04 | Uptake half saturation coefficient | Suspended detritus by omnivorous zooplankton |
| eHt | 518 | 8.94e-03 | 1.07e-03 | Background metabolic rate coefficient | Omnivorous zooplankton |
| hsNIT_phyt | 334 | -7.21e-03 | 1.15e-04 | Uptake half saturation coefficient | Nitrate by phytoplankton |
| qtena | 312 | 5.78e-03 | 3.38e-04 | Q10 | Autotrophic uptake |
| xxdt | 553 | 5.70e-03 | 4.62e-04 | Density dependent mortality coefficient | Phytoplankton lower layer |
| aH | 503 | -4.80e-03 | 2.32e-03 | Assimilation efficiency | Omnivorous zooplankton |
| used_benthst | 427 | -4.59e-03 | 2.56e-04 | Maximum uptake rate | Sediment detritus by suspension/deposit feeding benthos |
| xqs_p3 | 543 | -4.17e-03 | 1.97e-04 | Remobilisation parameter | Refractory to labile sediment detritus |
| uphyt_benthst | 423 | 3.91e-03 | 1.68e-04 | Maximum uptake rate | Phytoplankton by suspension/deposit feeding benthos |
| hsphyt_benths | 424 | -3.70e-03 | 2.90e-04 | Uptake half saturation coefficient | Phytoplankton by suspension/deposit feeding benthos |
| xxst | 552 | 3.66e-03 | 3.49e-04 | Density dependent mortality coefficient | Phytoplankton upper layer |
| udet_benthst | 425 | -3.23e-03 | 6.31e-05 | Maximum uptake rate | Suspended detritus by suspension/deposit feeding benthos |
| hsdet_benths | 426 | 2.96e-03 | 2.91e-04 | Uptake half saturation coefficient | Suspended detritus by suspension/deposit feeding benthos |
| xdsink_s | 572 | 2.95e-03 | 9.99e-05 | Sinking rate coefficient | Upper layer suspended detritus |
| qtenh | 313 | 2.60e-03 | 1.52e-04 | Q10 | Heterotrophic uptake |
| qtenm | 314 | -2.42e-03 | 2.15e-04 | Q10 | Metabolism and microbial rates |
| ubenths_benthct | 429 | 2.00e-03 | 1.25e-04 | Maximum uptake rate | Suspension/deposit feeding benthos by carnivore/scavenge feeding benthos |
| aBs | 507 | -1.64e-03 | 1.84e-04 | Assimilation efficiency | Suspension/deposit feeding benthos |
| eBst | 522 | 1.60e-03 | 8.98e-05 | Background metabolic rate coefficient | Suspension/deposit feeding benthos |
| xndt | 539 | 1.56e-03 | 3.34e-05 | Nitrification rate coefficient | Lower layer ammonia |
| hsphyt_omni | 342 | -1.53e-03 | 2.98e-04 | Uptake half saturation coefficient | Phytoplankton by omnivorous zooplankton |
| uomni_carnt | 349 | 1.28e-03 | 7.69e-05 | Maximum uptake rate | Omnivorous zooplankton by carnivorous zooplankton |
| hsomni_carn | 350 | -1.12e-03 | 1.33e-04 | Uptake half saturation coefficient | Omnivorous zooplankton by carnivorous zooplankton |
| aBc | 508 | 1.08e-03 | 2.88e-05 | Assimilation efficiency | Carnivore/scavenge feeding benthos |
| xqs_p1 | 541 | 1.07e-03 | 3.02e-05 | Conversion rate coefficient | Labile to refractory sediment detritus |
| xxbenths | 558 | 9.93e-04 | 1.38e-04 | Density dependent mortality coefficient | Suspension/deposit feeding benthos |
| xxbenthc | 559 | -9.64e-04 | 1.04e-04 | Density dependent mortality coefficient | Carnivore/scavenge feeding benthos |
| uphyt_omnit | 341 | 7.98e-04 | 2.48e-04 | Maximum uptake rate | Phytoplankton by omnivorous zooplankton |
| uomni_fishpt | 365 | 7.84e-04 | 2.32e-04 | Maximum uptake rate | Omnivorous zooplankton by planktivorous fish |
| aFp | 511 | 7.25e-04 | 1.88e-04 | Assimilation efficiency | Planktivorous fish |
| aC | 504 | 6.77e-04 | 2.38e-05 | Assimilation efficiency | Carnivorous zooplankton |
| hsomni_fishp | 366 | -6.63e-04 | 2.22e-04 | Uptake half saturation coefficient | Omnivorous zooplankton by planktivorous fish |
| eFpt | 526 | -6.57e-04 | 1.71e-04 | Background metabolic rate coefficient | Planktivorous fish |
| uNIT_icealgt | 329 | -6.08e-04 | 5.40e-05 | Maximum uptake rate | Nitrate by ice-algae |
| xxcarn | 555 | -5.97e-04 | 5.26e-05 | Density dependent mortality coefficient | Carnivorous zooplankton |
| udet_benthslart | 417 | -4.97e-04 | 5.24e-05 | Maximum uptake rate | Suspended detritus by suspension/deposit feeding benthos larvae |
| Lmaxup_icealg | 320 | 4.94e-04 | 6.90e-05 | Saturation light intensity for uptake | Ice-algae |
| hsbenths_benthc | 430 | -4.44e-04 | 2.64e-05 | Uptake half saturation coefficient | Suspension/deposit feeding benthos by carnivore/scavenge feeding benthos |
| qtenr | 315 | 4.07e-04 | 2.63e-05 | Q10 reference temperature | All temperature dependent processes |
| hsdet_benthslar | 418 | 3.01e-04 | 4.30e-05 | Uptake half saturation coefficient | Suspended detritus by suspension/deposit feeding benthos larvae |
| xxomni | 554 | 2.65e-04 | 2.05e-05 | Density dependent mortality coefficient | Omnivorous zooplankton |
| xxicealg | 551 | 2.23e-04 | 2.39e-05 | Density dependent mortality coefficient | Ice-algae |
| aBclar | 506 | 2.06e-04 | 1.13e-05 | Assimilation efficiency | Carnivore/scavenge feeding benthos larvae |
| hsNIT_icealg | 330 | 1.86e-04 | 1.35e-05 | Uptake half saturation coefficient | Nitrate by ice-algae |
| eBct | 523 | -1.85e-04 | 4.08e-06 | Background metabolic rate coefficient | Carnivore/scavenge feeding benthos |
| aBslar | 505 | -1.67e-04 | 1.12e-05 | Assimilation efficiency | Suspension/deposit feeding benthos larvae |
| xqs_p2 | 542 | -1.59e-04 | 3.03e-06 | Mineralistation rate scaling parameter | Refractory sediment detritus |
| ucorp_benthct | 435 | 1.47e-04 | 6.01e-06 | Maximum uptake rate | Corpses by carnivore/scavenge feeding benthos |
| xxcorp_det | 570 | -1.45e-04 | 1.16e-05 | Conversion rate coefficient | Corpses to labile sediment detritus |
| BS_fec | 614 | -1.20e-04 | 5.50e-06 | Annual fecundity | Suspension/deposit feeding benthos |
| fdriverbs_sp | 1048 | -1.20e-04 | 5.43e-06 | Spawning rate | Suspension/deposit feeding benthos |
| hscorp_benthc | 436 | -1.19e-04 | 9.66e-06 | Uptake half saturation coefficient | Corpses by carnivore/scavenge feeding benthos |
| uAMM_icealgt | 331 | 1.13e-04 | 8.16e-06 | Maximum uptake rate | Ammonia by ice-algae |
| fdriverbc_sp | 1050 | -9.59e-05 | 3.05e-06 | Spawning rate | Carnivore/scavenge feeding benthos |
| BC_fec | 615 | -9.59e-05 | 2.99e-06 | Annual fecundity | Carnivore/scavenge feeding benthos |
| eCt | 519 | -9.54e-05 | 4.84e-06 | Background metabolic rate coefficient | Carnivorous zooplankton |
| uicedet_omnit | 339 | -9.36e-05 | 6.60e-06 | Maximum uptake rate | Ice-detritus by omnivorous zooplankton |
| eBclart | 521 | -8.75e-05 | 4.33e-06 | Background metabolic rate coefficient | Carnivore/scavenge feeding benthos larvae |
| hsAMM_icealg | 332 | -7.93e-05 | 7.36e-06 | Uptake half saturation coefficient | Ammonia by ice-algae |
| hsphyt_benthclar | 420 | -6.56e-05 | 8.01e-06 | Uptake half saturation coefficient | Phytoplankton by carnivore/scavenge feeding benthos larvae |
| fdriverbs_rec | 1049 | 6.22e-05 | 6.95e-06 | Recruitment rate | Suspension/deposit feeding benthos |
| hsicedet_omni | 340 | 5.47e-05 | 5.12e-06 | Uptake half saturation coefficient | Ice-detritus by omnivorous zooplankton |
| eBslart | 520 | 5.46e-05 | 3.25e-06 | Background metabolic rate coefficient | Suspension/deposit feeding benthos larvae |
| xxpfish | 562 | -4.75e-05 | 1.61e-05 | Density dependent mortality coefficient | Planktivorous fish |
| uphyt_benthclart | 419 | 4.74e-05 | 3.36e-06 | Maximum uptake rate | Phytoplankton by carnivore/scavenge feeding benthos larvae |
| fdriverpfish_sp | 1044 | 4.44e-05 | 5.41e-06 | Spawning rate | Planktivorous fish |
| PF_fec | 612 | 4.43e-05 | 5.31e-06 | Annual fecundity | Planktivorous fish |
| ubenthclar_omnit | 347 | -2.71e-05 | 7.67e-07 | Maximum uptake rate | Carnivore/scavenge feeding benthos larvae by omnivorous zooplankton |
| hsbenthclar_omni | 348 | 2.70e-05 | 2.54e-06 | Uptake half saturation coefficient | Carnivore/scavenge feeding benthos larvae by omnivorous zooplankton |
| hsicealg_omni | 338 | -2.57e-05 | 2.73e-06 | Uptake half saturation coefficient | Ice-algae by omnivorous zooplankton |
| ubenths_fishdt | 397 | 2.45e-05 | 3.48e-06 | Maximum uptake rate | Suspension/deposit feeding benthos by demersal fish |
| xdsedt | 548 | 2.26e-05 | 2.32e-06 | Denitrification rate coefficient | Sediment porewater nitrate |
| aFd | 513 | 2.11e-05 | 2.85e-06 | Assimilation efficiency | Demersal fish |
| uicealg_omnit | 337 | 2.08e-05 | 1.11e-06 | Maximum uptake rate | Ice-algae by omnivorous zooplankton |
| eFdt | 528 | -2.05e-05 | 2.67e-06 | Background metabolic rate coefficient | Demersal fish |
| aFplar | 509 | 1.93e-05 | 2.81e-06 | Assimilation efficiency | Planktivorous fish larvae |
| udet_benthclart | 421 | 1.80e-05 | 2.45e-06 | Maximum uptake rate | Suspended detritus by carnivore/scavenge feeding benthos larvae |
| hscarn_fishp | 368 | -1.74e-05 | 1.83e-06 | Uptake half saturation coefficient | Carnivorous zooplankton by planktivorous fish |
| hsbenths_fishd | 398 | -1.70e-05 | 2.79e-06 | Uptake half saturation coefficient | Suspension/deposit feeding benthos by demersal fish |
| ucarn_fishpt | 367 | 1.62e-05 | 1.27e-06 | Maximum uptake rate | Carnivorous zooplankton by planktivorous fish |
| uomni_fishplart | 359 | 1.48e-05 | 2.45e-06 | Maximum uptake rate | Omnivorous zooplankton by planktivorous fish larvae |
| uomni_fishdlart | 389 | 1.44e-05 | 1.58e-06 | Maximum uptake rate | Omnivorous zooplankton by demersal fish larvae |
| hsomni_fishplar | 360 | -1.42e-05 | 2.63e-06 | Uptake half saturation coefficient | Omnivorous zooplankton by planktivorous fish larvae |
| hsomni_fishdlar | 390 | -1.39e-05 | 2.46e-06 | Uptake half saturation coefficient | Omnivorous zooplankton by demersal fish larvae |
| fdriverdfish_sp | 1046 | 1.33e-05 | 1.22e-06 | Spawning rate | Demersal fish |
| DF_fec | 613 | 1.33e-05 | 1.22e-06 | Annual fecundity | Demersal fish |
| fdriverbc_rec | 1051 | -1.02e-05 | 2.22e-06 | Recruitment rate | Carnivore/scavenge feeding benthos |
| hsbenthslar_omni | 346 | 9.90e-06 | 9.28e-07 | Uptake half saturation coefficient | Suspension/deposit feeding benthos larvae by omnivorous zooplankton |
| ubenthslar_omnit | 345 | -9.87e-06 | 4.38e-07 | Maximum uptake rate | Suspension/deposit feeding benthos larvae by omnivorous zooplankton |
| aFdlar | 510 | 7.73e-06 | 7.69e-07 | Assimilation efficiency | Demersal fish lavae |
| xpfish_migcoef | 576 | -7.66e-06 | 7.72e-07 | Active migration coefficient | Planktivorous fish |
| ubenthslar_carnt | 351 | 7.27e-06 | 3.94e-07 | Maximum uptake rate | Suspension/deposit feeding benthos larvae by carnivorous zooplankton |
| hsbenthslar_carn | 352 | -7.23e-06 | 7.56e-07 | Uptake half saturation coefficient | Suspension/deposit feeding benthos larvae by carnivorous zooplankton |
| ufishp_ceta | 481 | -5.24e-06 | 6.81e-07 | Maximum uptake rate | Planktivorous fish by cetaceans |
| xmt | 536 | -4.86e-06 | 2.87e-07 | Mineralisation rate coefficient | Suspended detritus |
| xxdfish | 564 | -4.59e-06 | 6.53e-07 | Density dependent mortality coefficient | Demersal fish |
| ubenthc_fishdt | 399 | -4.46e-06 | 7.11e-07 | Maximum uptake rate | Carnivore/scavenge feeding benthos by demersal fish |
| hsbenthslar_fishplar | 362 | -4.32e-06 | 7.07e-07 | Uptake half saturation coefficient | Suspension/deposit feeding benthos larvae by planktivorous fish larvae |
| ubenthslar_fishplart | 361 | 4.17e-06 | 6.18e-07 | Maximum uptake rate | Suspension/deposit feeding benthos larvae by planktivorous fish larvae |
| hsbenthc_fishd | 400 | 4.03e-06 | 8.47e-07 | Uptake half saturation coefficient | Carnivore/scavenge feeding benthos by demersal fish |
| hsdet_benthclar | 422 | 3.92e-06 | 2.57e-06 | Uptake half saturation coefficient | Suspended detritus by carnivore/scavenge feeding benthos larvae |
| bdapar_ceta | 493 | 3.83e-06 | 6.63e-07 | Bedding DeAngelis parameter | Cetaceans |
| ufishp_bird | 443 | -3.48e-06 | 6.14e-07 | Maximum uptake rate | Planktivorous fish by birds |
| xphytoseed | 575 | -2.70e-06 | 2.07e-07 | Proportion of ice-algae becoming phytoplankon on release | Linking ice-algae to phytoplankton |
| uphyt_benthslart | 415 | -2.48e-06 | 2.51e-07 | Maximum uptake rate | Phytoplankton by suspension/deposit feeding benthos larvae |
| CZ_inedible_biomass_i | 617 | -2.46e-06 | 2.56e-07 | Inedible biomass inshore | Carnivorous zooplankton |
| ufishplar_carnt | 355 | -2.30e-06 | 3.51e-07 | Maximum uptake rate | Planktivorous fish larvae by carnivorous zooplankton |
| hsfishplar_carn | 356 | 2.27e-06 | 4.11e-07 | Uptake half saturation coefficient | Planktivorous fish larvae by carnivorous zooplankton |
| hsfishp_bird | 444 | 2.18e-06 | 4.45e-07 | Uptake half saturation coefficient | Planktivorous fish by birds |
| useal_bear | 498 | 2.13e-06 | 4.32e-07 | Maximum uptake rate | Pinnipeds by maritime mammals |
| aseal | 515 | -2.08e-06 | 3.20e-07 | Assimilation efficiency | Pinnipeds |
| eFplart | 524 | -2.04e-06 | 2.92e-07 | Background metabolic rate coefficient | Planktivorous fish larvae |
| ufishp_seal | 460 | -1.94e-06 | 4.44e-07 | Maximum uptake rate | Planktivorous fish by pinnipeds |
| fdriverpfish_rec | 1045 | -1.85e-06 | 3.44e-07 | Recruitment rate | Planktivorous fish |
| ubenthslar_fishpt | 369 | 1.81e-06 | 3.17e-07 | Maximum uptake rate | Suspension/deposit feeding benthos larvae by planktivorous fish |
| hsbenthslar_fishp | 370 | -1.69e-06 | 3.07e-07 | Uptake half saturation coefficient | Suspension/deposit feeding benthos larvae by planktivorous fish |
| hscarn_fishd | 396 | 1.59e-06 | 2.83e-07 | Uptake half saturation coefficient | Carnivorous zooplankton by demersal fish |
| fdriverdfish_rec | 1047 | -1.59e-06 | 4.33e-07 | Recruitment rate | Demersal fish |
| ucarn_fishdt | 395 | -1.58e-06 | 1.87e-07 | Maximum uptake rate | Carnivorous zooplankton by demersal fish |
| hsfishp_ceta | 482 | 1.55e-06 | 3.02e-07 | Uptake half saturation coefficient | Planktivorous fish by cetaceans |
| ubenthclar_fishplart | 363 | 1.54e-06 | 3.03e-07 | Maximum uptake rate | Carnivore/scavenge feeding benthos larvae by planktivorous fish larvae |
| bda_par_bird | 453 | 1.53e-06 | 3.93e-07 | Bedding DeAngelis parameter | Birds |
| hsbenthclar_fishplar | 364 | -1.39e-06 | 2.74e-07 | Uptake half saturation coefficient | Carnivore/scavenge feeding benthos larvae by planktivorous fish larvae |
| ubenthclar_fishpt | 371 | 1.36e-06 | 3.29e-07 | Maximum uptake rate | Carnivore/scavenge feeding benthos larvae by planktivorous fish |
| hsfishp_seal | 461 | 1.34e-06 | 3.00e-07 | Uptake half saturation coefficient | Planktivorous fish by pinnipeds |
| hsbenthclar_fishp | 372 | -1.30e-06 | 2.79e-07 | Uptake half saturation coefficient | Carnivore/scavenge feeding benthos larvae by planktivorous fish |
| abird | 514 | -1.20e-06 | 2.56e-07 | Assimilation efficiency | Birds |
| CZ_inedible_biomass_o | 616 | -1.14e-06 | 2.33e-07 | Inedible biomass offshore | Carnivorous zooplankton |
| bdapar_bear | 502 | -1.14e-06 | 2.13e-07 | Bedding DeAngelis parameter | Maritime mammals |
| hsseal_bear | 499 | -1.11e-06 | 2.53e-07 | Uptake half saturation coefficient | Pinnipeds by maritime mammals |
| xxbird | 565 | 1.10e-06 | 3.02e-07 | Density dependent mortality coefficient | Birds |
| ucarn_seal | 454 | -1.10e-06 | 2.58e-07 | Maximum uptake rate | Carnivorous zooplankton by pinnipeds |
| hsphyt_benthslar | 416 | 1.09e-06 | 2.11e-07 | Uptake half saturation coefficient | Phytoplankton by suspension/deposit feeding benthos larvae |
| bdapar_seal | 472 | 9.90e-07 | 2.47e-07 | Bedding DeAngelis parameter | Pinnipeds |
| ufishp_fishdt | 405 | -8.56e-07 | 1.96e-07 | Maximum uptake rate | Planktivorous fish by demersal fish |
| hsfishp_fishd | 406 | 8.05e-07 | 2.37e-07 | Uptake half saturation coefficient | Planktivorous fish by demersal fish |
| fdriver_bird_exit | 1076 | 7.68e-07 | 2.66e-07 | Emigration rate | Birds |
| hscarn_seal | 455 | 7.51e-07 | 2.92e-07 | Uptake half saturation coefficient | Carnivorous zooplankton by pinnipeds |
| ubenths_seal | 456 | -6.00e-07 | 1.82e-07 | Maximum uptake rate | Suspension/deposit feeding benthos by pinnipeds |
| hssed_benths | 428 | 5.33e-07 | 1.72e-07 | Uptake half saturation coefficient | Sediment detritus by suspension/deposit feeding benthos |
| aceta | 516 | -4.87e-07 | 1.86e-07 | Assimilation efficiency | Cetaceans |
| icesensseal | 623 | 4.44e-07 | 1.98e-07 | Feeding half saturation sensitivity to ice cover | Pinnipeds |
| xxceta | 567 | 4.36e-07 | 2.06e-07 | Density dependent mortality coefficient | Cetaceans |
| hsbenths_seal | 457 | 4.26e-07 | 2.10e-07 | Uptake half saturation coefficient | Suspension/deposit feeding benthos by pinnipeds |
| hsomni_fishm | 378 | -3.95e-07 | 2.20e-07 | Uptake half saturation coefficient | Omnivorous zooplankton by migratory fish |
| icesensbird | 622 | 3.68e-07 | 2.00e-07 | Feeding half saturation sensitivity to ice cover | Birds |
| hsfishplar_fishd | 402 | 3.61e-07 | 3.08e-07 | Uptake half saturation coefficient | Planktivorous fish larvae by demersal fish |
| ufishplar_fishdt | 401 | -3.35e-07 | 1.74e-07 | Maximum uptake rate | Planktivorous fish larvae by demersal fish |
| ucorp_fishdt | 413 | 3.30e-07 | 2.27e-07 | Maximum uptake rate | Corpses by demersal fish |
| ucorp_bird | 451 | -3.24e-07 | 1.77e-07 | Maximum uptake rate | Corpses by birds |
| uomni_fishmt | 377 | 3.21e-07 | 1.67e-07 | Maximum uptake rate | Omnivorous zooplankton by migratory fish |
| icesensceta | 624 | 3.14e-07 | 2.19e-07 | Feeding half saturation sensitivity to ice cover | Cetaceans |
| ucarn_bird | 437 | -2.92e-07 | 1.67e-07 | Maximum uptake rate | Carnivorous zooplankton by birds |
| hscorp_fishd | 414 | -2.74e-07 | 1.93e-07 | Uptake half saturation coefficient | Corpses by demersal fish |
| hsbenthclar_carn | 354 | -2.69e-07 | 1.98e-07 | Uptake half saturation coefficient | Carnivore/scavenge feeding benthos larvae by carnivorous zooplankton |
| eFdlart | 525 | -2.41e-07 | 1.90e-07 | Background metabolic rate coefficient | Demersal fish larvae |
| uomni_ceta | 473 | -2.35e-07 | 1.54e-07 | Maximum uptake rate | Omnivorous zooplankton by cetaceans |
| ufishdlar_carnt | 357 | -2.23e-07 | 1.86e-07 | Maximum uptake rate | Demersal fish larvae by carnivorous zooplankton |
| hsfishplar_fishp | 376 | 2.23e-07 | 2.44e-07 | Uptake half saturation coefficient | Planktivorous fish larvae by planktivorous fish |
| xmicet | 533 | -2.19e-07 | 2.48e-07 | Mineralisation rate coefficient | Sea-ice |
| hsfishdlar_carn | 358 | 2.10e-07 | 3.10e-07 | Uptake half saturation coefficient | Demersal fish larvae by carnivorous zooplankton |
| ubenthclar_carnt | 353 | 2.10e-07 | 1.92e-07 | Maximum uptake rate | Carnivore/scavenge feeding benthos larvae by carnivorous zooplankton |
| icesensbear | 625 | -2.09e-07 | 2.54e-07 | Feeding half saturation sensitivity to ice cover | Maritime mammals |
| xxbear | 568 | -1.99e-07 | 1.75e-07 | Density dependent mortality coefficient | Maritime mammals |
| xdfish_migcoef | 578 | 1.93e-07 | 2.37e-07 | Active migration coefficient | Demersal fish |
| abear | 517 | 1.93e-07 | 2.06e-07 | Assimilation efficiency | Maritime mammals |
| ucarn_ceta | 475 | -1.92e-07 | 1.85e-07 | Maximum uptake rate | Carnivorous zooplankton by cetaceans |
| fdrivermfish_em | 1053 | -1.87e-07 | 2.29e-07 | Emigration rate | Migratory fish |
| ufishplar_fishpt | 375 | -1.82e-07 | 2.19e-07 | Maximum uptake rate | Planktivorous fish larvae by planktivorous fish |
| hscorp_bird | 452 | 1.73e-07 | 2.28e-07 | Uptake half saturation coefficient | Corpses by birds |
| fdriver_ceta_exit | 1080 | 1.71e-07 | 1.67e-07 | Emigration rate | Cetaceans |
| xxbenthslar | 556 | 1.50e-07 | 1.78e-07 | Density dependent mortality coefficient | Suspension/deposit feeding benthos larvae |
| fdrivermfish_im | 1052 | 1.40e-07 | 1.89e-07 | Immigration rate | Migratory fish |
| xnsedt | 546 | 1.40e-07 | 2.28e-07 | Nitrification rate coefficient | Sediment porewater ammonia |
| hscarn_bird | 438 | 1.38e-07 | 2.22e-07 | Uptake half saturation coefficient | Carnivorous zooplankton by birds&mammala |
| ubenthc_seal | 458 | -1.26e-07 | 2.17e-07 | Maximum uptake rate | Carnivore/scavenge feeding benthos by pinnipeds |
| ubird_bear | 496 | 1.23e-07 | 1.50e-07 | Maximum uptake rate | Birds by maritime mammals |
| hsbenthc_seal | 459 | 1.13e-07 | 2.20e-07 | Uptake half saturation coefficient | Carnivore/scavenge feeding benthos by pinnipeds |
| ufishd_ceta | 485 | -1.12e-07 | 1.55e-07 | Maximum uptake rate | Demersal fish by cetaceans |
| fdriver_bird_return | 1077 | 9.17e-08 | 1.83e-07 | Immigration rate | Birds |
| uceta_bear | 500 | 8.84e-08 | 1.70e-07 | Maximum uptake rate | Cetaceans by maritime mammals |
| ecetat | 531 | 8.51e-08 | 1.84e-07 | Background metabolic rate coefficient | Cetaceans |
| hsbird_bear | 497 | -6.73e-08 | 1.47e-07 | Uptake half saturation coefficient | Birds by maritime mammals |
| Phytoplankton fratio - Sourced for the East Greenland Shelf | | | | | |
| fdriversslight | 1001 | -2.80e-02 | 5.54e-04 | Sea surface irradiance | Inshore and offshore zones |
| fdriversi_icecov | 1067 | 2.30e-02 | 1.88e-03 | Ice cover proportion in ice affected area | Inshore zone |
| inshore_phyt_prop_depth | 55 | 1.84e-02 | 2.68e-03 | Proportion of depth range occupied | Phytoplankton inshore |
| lightSPM_slope | 54 | 1.75e-02 | 1.20e-03 | Coefficient | Light attenuation coefficient vs SPM |
| thik_si | 3 | 1.65e-02 | 1.03e-03 | Vertical thickness | Inshore zone |
| fdriverboundso_amm | 1021 | -1.61e-02 | 2.15e-04 | Boundary concentration | Upper layer offshore ammonia |
| fdriverboundso_phyt | 1027 | -1.33e-02 | 3.14e-04 | Boundary concentration | Upper layer offshore phytoplankton |
| fdriversi_logespm | 1003 | 1.33e-02 | 1.41e-03 | Suspended particulate matter | Inshore zone |
| fdriverso_inflow | 1008 | 1.03e-02 | 5.72e-04 | Boundary volume inflow rate | Inshore zone |
| lightSPM_intercept | 53 | 9.86e-03 | 4.86e-04 | Intercept | Light attenuation coefficient vs SPM |
| fdriverboundso_nit | 1024 | 7.42e-03 | 7.22e-04 | Boundary concentration | Upper layer offshore nitrate |
| thik_d | 2 | 6.04e-03 | 1.63e-04 | Vertical thickness | Offshore zone lower layer |
| fdriverboundso_det | 1018 | -5.53e-03 | 1.18e-04 | Boundary concentration | Upper layer offshore detritus |
| Reflect | 58 | 5.20e-03 | 2.09e-04 | Proportion of incoming light reflected by ice or snow | Ice and snow |
| thik_so | 1 | 4.35e-03 | 1.61e-04 | Vertical thickness | Offshore zone upper layer |
| fdriverso_logespm | 1002 | 4.30e-03 | 5.18e-04 | Suspended particulate matter | Offshore zone |
| fdriverso_icecov | 1056 | 2.89e-03 | 1.15e-03 | Ice cover proportion in ice affected area | Offshore zone |
| fdriverso_si_flow | 1014 | -2.08e-03 | 5.09e-05 | Volume exchange rate | Offshore to inshore zone |
| Kice | 60 | 1.91e-03 | 1.31e-04 | Light attenuation coefficient of ice | Sea-ice |
| fdriversi_icefree | 1066 | -1.90e-03 | 6.61e-05 | Ice-free area proportion | Inshore zone |
| fdriversi_outflow | 1013 | 1.44e-03 | 3.86e-05 | Volume outflow rate | Inshore zone |
| fdriversi_icethick | 1068 | 9.51e-04 | 5.89e-05 | Ice thickness | Inshore zone |
| fdriverso_icethick | 1057 | 9.33e-04 | 8.78e-05 | Ice thickness | Offshore zone |
| thik_b | 4 | 7.33e-04 | 1.57e-04 | Vertical thickness | Benthic boundary feeding layer |
| fdriverd_inflow | 1009 | 5.75e-04 | 3.19e-05 | Boundary volume inflow rate | Lower layer offshore |
| fdriverboundsi_phyt | 1029 | -5.44e-04 | 1.02e-05 | Boundary concentration | Inshore phytoplankton |
| fdriverso_temp | 1004 | 4.30e-04 | 1.77e-05 | Temperature | Offshore zone upper layer |
| fdriversi_inflow | 1010 | -3.85e-04 | 8.00e-06 | Boundary volume inflow rate | Inshore zone |
| Ksnow | 59 | 3.38e-04 | 2.55e-05 | Light attenuation coefficient of snow | Snow |
| fdriverv_dif | 1007 | 3.26e-04 | 1.97e-05 | Vertical diffusion rate | Offshore zone |
| fdriverso_icefree | 1055 | -3.08e-04 | 4.28e-05 | Ice-free area proportion | Offshore zone |
| fdriversi_temp | 1006 | 2.73e-04 | 3.06e-05 | Temperature | Inshore zone |
| fdriversi_snowthick | 1069 | 2.27e-04 | 1.64e-05 | Snow thickness | Inshore zone |
| fdriverboundd_nit | 1025 | 2.27e-04 | 1.31e-05 | Boundary concentration | Lower layer nitrate |
| fdriverboundsi_amm | 1023 | -2.06e-04 | 3.88e-06 | Boundary concentration | Inshore ammonia |
| fdriverboundd_phyt | 1028 | -2.03e-04 | 5.08e-06 | Boundary concentration | Lower layer phytoplankton |
| fdriverboundd_amm | 1022 | -1.78e-04 | 2.78e-06 | Boundary concentration | Lower layer ammonia |
| fdriverso_snowthick | 1058 | 1.10e-04 | 1.32e-05 | Snow thickness | Offshore zone |
| fdriverboundsi_det | 1020 | -1.05e-04 | 2.26e-06 | Boundary concentration | Inshore detritus |
| fdriverboundsi_nit | 1026 | -1.03e-04 | 1.02e-05 | Boundary concentration | Inshore nitrate |
| fdriverboundd_det | 1019 | -9.62e-05 | 1.04e-05 | Boundary concentration | Lower layer detritus |
| porosity_s1 | 28 | 6.07e-05 | 3.24e-06 | Sediment porosity | Inshore muddy sediments |
| fdriverd_temp | 1005 | -4.74e-05 | 4.90e-06 | Temperature | Lower layer offshore |
| fdriverso_atm_amm | 1033 | -2.85e-05 | 4.25e-07 | Atmospheric deposition rate | Offshore ammonia |
| sed_wat_dif_s1 | 34 | -2.62e-05 | 2.59e-06 | Hydraulic conductivity | Inshore muddy sediments |
| Poricez | 57 | -2.50e-05 | 2.10e-06 | Maximum thickness of the porous ice layer | Sea-ice |
| fdriversi_atm_amm | 1035 | -1.27e-05 | 2.99e-07 | Atmospheric deposition rate | Inshore ammonia |
| fdriverboundriv_amm | 1030 | -8.83e-06 | 3.36e-07 | Boundary concentration | River ammonia |
| porosity_s2 | 29 | 5.54e-06 | 3.77e-07 | Sediment porosity | Inshore sandy sediments |
| fdriverriver | 1017 | 2.83e-06 | 3.11e-07 | River volume inflow rate | Inshore zone |
| fdriverboundriv_nit | 1031 | -1.63e-06 | 2.30e-07 | Boundary concentration | River nitrate |
| porosity_s3 | 30 | 1.28e-06 | 2.21e-07 | Sediment porosity | Inshore coarse sediments |
| sed_wat_dif_s2 | 35 | -1.04e-06 | 2.02e-07 | Hydraulic conductivity | Inshore sandy sediments |
| sed_wat_dif_d1 | 37 | -9.03e-07 | 2.66e-07 | Hydraulic conductivity | Offshore muddy sediments |
| fdriverso_atm_nit | 1034 | 6.22e-07 | 2.58e-07 | Atmospheric deposition rate | Offshore nitrate |
| porosity_d1 | 31 | 5.89e-07 | 1.90e-07 | Sediment porosity | Offshore muddy sediments |
| fdriversi_atm_nit | 1036 | -3.27e-07 | 2.48e-07 | Atmospheric deposition rate | Inshore nitrate |
| porosity_d3 | 33 | -1.11e-07 | 2.46e-07 | Sediment porosity | Offshore coarse sediments |
| sed_wat_dif_s3 | 36 | -8.12e-08 | 1.54e-07 | Hydraulic conductivity | Inshore coarse sediments |
| fdriversi_airtemp | 1065 | -7.50e-08 | 1.66e-07 | Air temperature | Inshore zone |
| Phytoplankton net primary production - Borrowed from Barents Sea | | | | | |
| Lmaxup_phyt | 316 | -4.99e+02 | 4.71e+01 | Saturation light intensity for uptake | Nutrient by phytoplankton |
| uNIT_phytt | 333 | 4.62e+02 | 1.04e+01 | Maximum uptake rate | Nitrate by phytoplankton |
| xxst | 552 | -3.47e+02 | 2.01e+01 | Density dependent mortality coefficient | Phytoplankton upper layer |
| aH | 503 | 2.53e+02 | 2.14e+01 | Assimilation efficiency | Omnivorous zooplankton |
| eHt | 518 | -2.00e+02 | 6.11e+00 | Background metabolic rate coefficient | Omnivorous zooplankton |
| hsNIT_phyt | 334 | -1.86e+02 | 7.61e+00 | Uptake half saturation coefficient | Nitrate by phytoplankton |
| uphyt_omnit | 341 | 1.52e+02 | 3.55e+00 | Maximum uptake rate | Phytoplankton by omnivorous zooplankton |
| udet_omnit | 343 | 1.43e+02 | 1.27e+01 | Maximum uptake rate | Suspended detritus by omnivorous zooplankton |
| hsphyt_omni | 342 | -1.30e+02 | 6.77e+00 | Uptake half saturation coefficient | Phytoplankton by omnivorous zooplankton |
| hsdet_omni | 344 | -1.27e+02 | 2.55e+00 | Uptake half saturation coefficient | Suspended detritus by omnivorous zooplankton |
| qtena | 312 | -1.10e+02 | 5.77e+00 | Q10 | Autotrophic uptake |
| qtenh | 313 | -6.83e+01 | 2.48e+00 | Q10 | Heterotrophic uptake |
| xxdt | 553 | -5.78e+01 | 3.57e+00 | Density dependent mortality coefficient | Phytoplankton lower layer |
| qtenm | 314 | 4.51e+01 | 2.73e+00 | Q10 | Metabolism and microbial rates |
| aBs | 507 | 4.11e+01 | 1.74e+00 | Assimilation efficiency | Suspension/deposit feeding benthos |
| xdsink_s | 572 | -3.05e+01 | 7.08e-01 | Sinking rate coefficient | Upper layer suspended detritus |
| uAMM_phytt | 335 | 2.64e+01 | 6.71e-01 | Maximum uptake rate | Ammonia by phytoplankton |
| uomni_carnt | 349 | -2.49e+01 | 1.07e+00 | Maximum uptake rate | Omnivorous zooplankton by carnivorous zooplankton |
| udet_benthst | 425 | 2.38e+01 | 1.11e+00 | Maximum uptake rate | Suspended detritus by suspension/deposit feeding benthos |
| hsdet_benths | 426 | -2.29e+01 | 2.18e+00 | Uptake half saturation coefficient | Suspended detritus by suspension/deposit feeding benthos |
| hsAMM_phyt | 336 | -2.22e+01 | 1.41e+00 | Uptake half saturation coefficient | Ammonia by phytoplankton |
| hsomni_carn | 350 | 2.20e+01 | 2.14e+00 | Uptake half saturation coefficient | Omnivorous zooplankton by carnivorous zooplankton |
| used_benthst | 427 | 1.86e+01 | 1.56e+00 | Maximum uptake rate | Sediment detritus by suspension/deposit feeding benthos |
| xqs_p3 | 543 | 1.59e+01 | 1.15e+00 | Remobilisation parameter | Refractory to labile sediment detritus |
| uomni_fishpt | 365 | -1.57e+01 | 4.94e+00 | Maximum uptake rate | Omnivorous zooplankton by planktivorous fish |
| eBst | 522 | -1.55e+01 | 8.77e-01 | Background metabolic rate coefficient | Suspension/deposit feeding benthos |
| eFpt | 526 | 1.48e+01 | 2.47e+00 | Background metabolic rate coefficient | Planktivorous fish |
| aFp | 511 | -1.48e+01 | 4.15e+00 | Assimilation efficiency | Planktivorous fish |
| hsomni_fishp | 366 | 1.47e+01 | 3.48e+00 | Uptake half saturation coefficient | Omnivorous zooplankton by planktivorous fish |
| aC | 504 | -1.35e+01 | 3.51e-01 | Assimilation efficiency | Carnivorous zooplankton |
| xxcarn | 555 | 1.20e+01 | 9.02e-01 | Density dependent mortality coefficient | Carnivorous zooplankton |
| xxbenths | 558 | -9.74e+00 | 8.19e-01 | Density dependent mortality coefficient | Suspension/deposit feeding benthos |
| qtenr | 315 | -9.71e+00 | 5.44e-01 | Q10 reference temperature | All temperature dependent processes |
| uphyt_benthst | 423 | 9.37e+00 | 8.82e-01 | Maximum uptake rate | Phytoplankton by suspension/deposit feeding benthos |
| xxomni | 554 | -9.07e+00 | 2.18e-01 | Density dependent mortality coefficient | Omnivorous zooplankton |
| hsphyt_benths | 424 | -8.60e+00 | 4.54e-01 | Uptake half saturation coefficient | Phytoplankton by suspension/deposit feeding benthos |
| ubenths_benthct | 429 | -7.77e+00 | 5.16e-01 | Maximum uptake rate | Suspension/deposit feeding benthos by carnivore/scavenge feeding benthos |
| uphyt_benthclart | 419 | 5.44e+00 | 2.90e-01 | Maximum uptake rate | Phytoplankton by carnivore/scavenge feeding benthos larvae |
| hsphyt_benthclar | 420 | -4.78e+00 | 4.94e-01 | Uptake half saturation coefficient | Phytoplankton by carnivore/scavenge feeding benthos larvae |
| Lmaxup_icealg | 320 | -4.61e+00 | 6.26e-01 | Saturation light intensity for uptake | Ice-algae |
| uNIT_icealgt | 329 | 4.07e+00 | 2.25e-01 | Maximum uptake rate | Nitrate by ice-algae |
| fdriverbc_sp | 1050 | 3.46e+00 | 9.58e-02 | Spawning rate | Carnivore/scavenge feeding benthos |
| BC_fec | 615 | 3.46e+00 | 9.61e-02 | Annual fecundity | Carnivore/scavenge feeding benthos |
| aBc | 508 | -2.59e+00 | 2.11e-01 | Assimilation efficiency | Carnivore/scavenge feeding benthos |
| udet_benthslart | 417 | -2.23e+00 | 2.25e-01 | Maximum uptake rate | Suspended detritus by suspension/deposit feeding benthos larvae |
| xmsedt | 544 | -2.22e+00 | 2.52e-01 | Mineralisation rate coefficient | Labile sediment detritus |
| xxbenthc | 559 | 2.14e+00 | 2.07e-01 | Density dependent mortality coefficient | Carnivore/scavenge feeding benthos |
| xxicealg | 551 | -2.05e+00 | 2.16e-01 | Density dependent mortality coefficient | Ice-algae |
| eCt | 519 | 1.91e+00 | 3.76e-02 | Background metabolic rate coefficient | Carnivorous zooplankton |
| aBclar | 506 | 1.69e+00 | 1.25e-01 | Assimilation efficiency | Carnivore/scavenge feeding benthos larvae |
| hsbenths_benthc | 430 | 1.68e+00 | 9.29e-02 | Uptake half saturation coefficient | Suspension/deposit feeding benthos by carnivore/scavenge feeding benthos |
| BS_fec | 614 | -1.37e+00 | 6.55e-02 | Annual fecundity | Suspension/deposit feeding benthos |
| fdriverbs_sp | 1048 | -1.37e+00 | 6.57e-02 | Spawning rate | Suspension/deposit feeding benthos |
| eBclart | 521 | -1.37e+00 | 6.33e-02 | Background metabolic rate coefficient | Carnivore/scavenge feeding benthos larvae |
| hsNIT_icealg | 330 | -1.26e+00 | 9.04e-02 | Uptake half saturation coefficient | Nitrate by ice-algae |
| hsdet_benthslar | 418 | 1.24e+00 | 1.61e-01 | Uptake half saturation coefficient | Suspended detritus by suspension/deposit feeding benthos larvae |
| fdriverbc_rec | 1051 | -1.12e+00 | 8.07e-02 | Recruitment rate | Carnivore/scavenge feeding benthos |
| xqs_p1 | 541 | -1.06e+00 | 3.34e-02 | Conversion rate coefficient | Labile to refractory sediment detritus |
| xxpfish | 562 | 9.85e-01 | 2.78e-01 | Density dependent mortality coefficient | Planktivorous fish |
| fdriverpfish_sp | 1044 | -9.53e-01 | 1.17e-01 | Spawning rate | Planktivorous fish |
| PF_fec | 612 | -9.52e-01 | 1.16e-01 | Annual fecundity | Planktivorous fish |
| xdsedt | 548 | -8.25e-01 | 2.04e-02 | Denitrification rate coefficient | Sediment porewater nitrate |
| fdriverbs_rec | 1049 | 8.13e-01 | 7.12e-02 | Recruitment rate | Suspension/deposit feeding benthos |
| aBslar | 505 | -6.29e-01 | 8.10e-02 | Assimilation efficiency | Suspension/deposit feeding benthos larvae |
| eFdt | 528 | 6.18e-01 | 5.49e-02 | Background metabolic rate coefficient | Demersal fish |
| aFd | 513 | -6.05e-01 | 4.07e-02 | Assimilation efficiency | Demersal fish |
| uicedet_omnit | 339 | 5.63e-01 | 3.98e-02 | Maximum uptake rate | Ice-detritus by omnivorous zooplankton |
| ubenths_fishdt | 397 | -4.96e-01 | 3.16e-02 | Maximum uptake rate | Suspension/deposit feeding benthos by demersal fish |
| eBct | 523 | 4.55e-01 | 3.45e-02 | Background metabolic rate coefficient | Carnivore/scavenge feeding benthos |
| hscarn_fishp | 368 | 4.06e-01 | 3.28e-02 | Uptake half saturation coefficient | Carnivorous zooplankton by planktivorous fish |
| aFplar | 509 | -4.05e-01 | 5.80e-02 | Assimilation efficiency | Planktivorous fish larvae |
| uAMM_icealgt | 331 | 4.03e-01 | 2.73e-02 | Maximum uptake rate | Ammonia by ice-algae |
| uomni_fishdlart | 389 | -3.90e-01 | 2.65e-02 | Maximum uptake rate | Omnivorous zooplankton by demersal fish larvae |
| hsomni_fishdlar | 390 | 3.84e-01 | 6.36e-02 | Uptake half saturation coefficient | Omnivorous zooplankton by demersal fish larvae |
| ucarn_fishpt | 367 | -3.67e-01 | 3.01e-02 | Maximum uptake rate | Carnivorous zooplankton by planktivorous fish |
| hsAMM_icealg | 332 | -3.46e-01 | 3.48e-02 | Uptake half saturation coefficient | Ammonia by ice-algae |
| fdriverdfish_sp | 1046 | -3.43e-01 | 2.08e-02 | Spawning rate | Demersal fish |
| DF_fec | 613 | -3.43e-01 | 2.02e-02 | Annual fecundity | Demersal fish |
| xxcorp_det | 570 | 3.31e-01 | 3.21e-02 | Conversion rate coefficient | Corpses to labile sediment detritus |
| hsbenths_fishd | 398 | 3.26e-01 | 3.91e-02 | Uptake half saturation coefficient | Suspension/deposit feeding benthos by demersal fish |
| uomni_fishplart | 359 | -3.23e-01 | 5.19e-02 | Maximum uptake rate | Omnivorous zooplankton by planktivorous fish larvae |
| ucorp_benthct | 435 | -3.17e-01 | 3.73e-02 | Maximum uptake rate | Corpses by carnivore/scavenge feeding benthos |
| hsomni_fishplar | 360 | 3.16e-01 | 4.87e-02 | Uptake half saturation coefficient | Omnivorous zooplankton by planktivorous fish larvae |
| hscorp_benthc | 436 | 3.08e-01 | 3.19e-02 | Uptake half saturation coefficient | Corpses by carnivore/scavenge feeding benthos |
| hsicealg_omni | 338 | 2.60e-01 | 2.81e-02 | Uptake half saturation coefficient | Ice-algae by omnivorous zooplankton |
| eBslart | 520 | 2.34e-01 | 2.93e-02 | Background metabolic rate coefficient | Suspension/deposit feeding benthos larvae |
| hsdet_benthclar | 422 | -2.18e-01 | 3.32e-02 | Uptake half saturation coefficient | Suspended detritus by carnivore/scavenge feeding benthos larvae |
| uicealg_omnit | 337 | -1.99e-01 | 7.31e-03 | Maximum uptake rate | Ice-algae by omnivorous zooplankton |
| hsicedet_omni | 340 | -1.88e-01 | 3.79e-02 | Uptake half saturation coefficient | Ice-detritus by omnivorous zooplankton |
| aFdlar | 510 | -1.87e-01 | 1.21e-02 | Assimilation efficiency | Demersal fish lavae |
| xpfish_migcoef | 576 | 1.85e-01 | 1.58e-02 | Active migration coefficient | Planktivorous fish |
| xndt | 539 | 1.78e-01 | 2.29e-02 | Nitrification rate coefficient | Lower layer ammonia |
| hsbenthslar_omni | 346 | -1.75e-01 | 1.71e-02 | Uptake half saturation coefficient | Suspension/deposit feeding benthos larvae by omnivorous zooplankton |
| ubenthslar_omnit | 345 | 1.74e-01 | 9.49e-03 | Maximum uptake rate | Suspension/deposit feeding benthos larvae by omnivorous zooplankton |
| udet_benthclart | 421 | -1.59e-01 | 2.68e-02 | Maximum uptake rate | Suspended detritus by carnivore/scavenge feeding benthos larvae |
| xxdfish | 564 | 1.41e-01 | 1.80e-02 | Density dependent mortality coefficient | Demersal fish |
| ufishp_ceta | 481 | 1.12e-01 | 1.40e-02 | Maximum uptake rate | Planktivorous fish by cetaceans |
| xqs_p2 | 542 | 1.10e-01 | 3.80e-03 | Mineralistation rate scaling parameter | Refractory sediment detritus |
| uphyt_benthslart | 415 | 1.08e-01 | 4.61e-03 | Maximum uptake rate | Phytoplankton by suspension/deposit feeding benthos larvae |
| fdriverdfish_rec | 1047 | 1.02e-01 | 1.54e-02 | Recruitment rate | Demersal fish |
| hsbenthclar_omni | 348 | -1.02e-01 | 9.92e-03 | Uptake half saturation coefficient | Carnivore/scavenge feeding benthos larvae by omnivorous zooplankton |
| ubenthclar_omnit | 347 | 1.01e-01 | 6.53e-03 | Maximum uptake rate | Carnivore/scavenge feeding benthos larvae by omnivorous zooplankton |
| ubenthc_fishdt | 399 | -1.00e-01 | 6.23e-03 | Maximum uptake rate | Carnivore/scavenge feeding benthos by demersal fish |
| hsbenthslar_fishplar | 362 | 9.02e-02 | 1.28e-02 | Uptake half saturation coefficient | Suspension/deposit feeding benthos larvae by planktivorous fish larvae |
| hsbenthc_fishd | 400 | 8.97e-02 | 1.06e-02 | Uptake half saturation coefficient | Carnivore/scavenge feeding benthos by demersal fish |
| ubenthslar_fishplart | 361 | -8.63e-02 | 1.19e-02 | Maximum uptake rate | Suspension/deposit feeding benthos larvae by planktivorous fish larvae |
| bdapar_ceta | 493 | -8.25e-02 | 1.13e-02 | Bedding DeAngelis parameter | Cetaceans |
| hsphyt_benthslar | 416 | -7.94e-02 | 5.38e-03 | Uptake half saturation coefficient | Phytoplankton by suspension/deposit feeding benthos larvae |
| ufishp_bird | 443 | 7.37e-02 | 1.37e-02 | Maximum uptake rate | Planktivorous fish by birds |
| hsbenthslar_carn | 352 | 6.65e-02 | 6.72e-03 | Uptake half saturation coefficient | Suspension/deposit feeding benthos larvae by carnivorous zooplankton |
| ubenthslar_carnt | 351 | -6.48e-02 | 4.74e-03 | Maximum uptake rate | Suspension/deposit feeding benthos larvae by carnivorous zooplankton |
| CZ_inedible_biomass_i | 617 | 6.31e-02 | 5.63e-03 | Inedible biomass inshore | Carnivorous zooplankton |
| hsbenthclar_carn | 354 | 6.14e-02 | 5.93e-03 | Uptake half saturation coefficient | Carnivore/scavenge feeding benthos larvae by carnivorous zooplankton |
| ubenthclar_carnt | 353 | -5.89e-02 | 3.87e-03 | Maximum uptake rate | Carnivore/scavenge feeding benthos larvae by carnivorous zooplankton |
| hsfishplar_carn | 356 | -4.97e-02 | 6.87e-03 | Uptake half saturation coefficient | Planktivorous fish larvae by carnivorous zooplankton |
| ufishplar_carnt | 355 | 4.91e-02 | 6.14e-03 | Maximum uptake rate | Planktivorous fish larvae by carnivorous zooplankton |
| hsfishp_bird | 444 | -4.59e-02 | 8.27e-03 | Uptake half saturation coefficient | Planktivorous fish by birds |
| useal_bear | 498 | -4.29e-02 | 6.89e-03 | Maximum uptake rate | Pinnipeds by maritime mammals |
| aseal | 515 | 4.29e-02 | 8.64e-03 | Assimilation efficiency | Pinnipeds |
| eFplart | 524 | 4.28e-02 | 6.08e-03 | Background metabolic rate coefficient | Planktivorous fish larvae |
| ubenthclar_fishplart | 363 | -3.99e-02 | 6.63e-03 | Maximum uptake rate | Carnivore/scavenge feeding benthos larvae by planktivorous fish larvae |
| ufishp_seal | 460 | 3.91e-02 | 8.76e-03 | Maximum uptake rate | Planktivorous fish by pinnipeds |
| hsbenthclar_fishplar | 364 | 3.85e-02 | 6.47e-03 | Uptake half saturation coefficient | Carnivore/scavenge feeding benthos larvae by planktivorous fish larvae |
| hsbenthclar_fishp | 372 | 3.78e-02 | 6.21e-03 | Uptake half saturation coefficient | Carnivore/scavenge feeding benthos larvae by planktivorous fish |
| ubenthclar_fishpt | 371 | -3.78e-02 | 7.05e-03 | Maximum uptake rate | Carnivore/scavenge feeding benthos larvae by planktivorous fish |
| hsbenthslar_fishp | 370 | 3.37e-02 | 5.37e-03 | Uptake half saturation coefficient | Suspension/deposit feeding benthos larvae by planktivorous fish |
| ubenthslar_fishpt | 369 | -3.37e-02 | 6.06e-03 | Maximum uptake rate | Suspension/deposit feeding benthos larvae by planktivorous fish |
| hsfishp_ceta | 482 | -3.35e-02 | 5.72e-03 | Uptake half saturation coefficient | Planktivorous fish by cetaceans |
| bda_par_bird | 453 | -3.26e-02 | 7.11e-03 | Bedding DeAngelis parameter | Birds |
| fdriverpfish_rec | 1045 | 3.15e-02 | 4.62e-03 | Recruitment rate | Planktivorous fish |
| xphytoseed | 575 | -3.01e-02 | 3.55e-03 | Proportion of ice-algae becoming phytoplankon on release | Linking ice-algae to phytoplankton |
| ucarn_fishdt | 395 | 2.82e-02 | 3.12e-03 | Maximum uptake rate | Carnivorous zooplankton by demersal fish |
| hsfishp_seal | 461 | -2.77e-02 | 6.02e-03 | Uptake half saturation coefficient | Planktivorous fish by pinnipeds |
| hscarn_fishd | 396 | -2.66e-02 | 3.99e-03 | Uptake half saturation coefficient | Carnivorous zooplankton by demersal fish |
| abird | 514 | 2.63e-02 | 5.14e-03 | Assimilation efficiency | Birds |
| CZ_inedible_biomass_o | 616 | 2.47e-02 | 3.64e-03 | Inedible biomass offshore | Carnivorous zooplankton |
| bdapar_bear | 502 | 2.38e-02 | 4.25e-03 | Bedding DeAngelis parameter | Maritime mammals |
| ucarn_seal | 454 | 2.22e-02 | 4.97e-03 | Maximum uptake rate | Carnivorous zooplankton by pinnipeds |
| xxbird | 565 | -2.21e-02 | 5.29e-03 | Density dependent mortality coefficient | Birds |
| hsseal_bear | 499 | 2.20e-02 | 5.09e-03 | Uptake half saturation coefficient | Pinnipeds by maritime mammals |
| bdapar_seal | 472 | -2.07e-02 | 5.47e-03 | Bedding DeAngelis parameter | Pinnipeds |
| xmt | 536 | -1.70e-02 | 2.97e-03 | Mineralisation rate coefficient | Suspended detritus |
| ufishp_fishdt | 405 | 1.70e-02 | 4.00e-03 | Maximum uptake rate | Planktivorous fish by demersal fish |
| uomni_fishmt | 377 | -1.68e-02 | 3.40e-03 | Maximum uptake rate | Omnivorous zooplankton by migratory fish |
| hsfishp_fishd | 406 | -1.67e-02 | 5.47e-03 | Uptake half saturation coefficient | Planktivorous fish by demersal fish |
| hsomni_fishm | 378 | 1.64e-02 | 4.33e-03 | Uptake half saturation coefficient | Omnivorous zooplankton by migratory fish |
| fdrivermfish_im | 1052 | -1.61e-02 | 2.87e-03 | Immigration rate | Migratory fish |
| fdriver_bird_exit | 1076 | -1.59e-02 | 4.84e-03 | Emigration rate | Birds |
| hscarn_seal | 455 | -1.48e-02 | 4.11e-03 | Uptake half saturation coefficient | Carnivorous zooplankton by pinnipeds |
| ubenths_seal | 456 | 1.17e-02 | 4.27e-03 | Maximum uptake rate | Suspension/deposit feeding benthos by pinnipeds |
| ucorp_fishdt | 413 | -1.15e-02 | 3.14e-03 | Maximum uptake rate | Corpses by demersal fish |
| hscorp_fishd | 414 | 1.11e-02 | 4.00e-03 | Uptake half saturation coefficient | Corpses by demersal fish |
| aceta | 516 | 1.10e-02 | 3.60e-03 | Assimilation efficiency | Cetaceans |
| icesensseal | 623 | -8.84e-03 | 3.03e-03 | Feeding half saturation sensitivity to ice cover | Pinnipeds |
| icesensbird | 622 | -8.55e-03 | 3.18e-03 | Feeding half saturation sensitivity to ice cover | Birds |
| hsfishdlar_carn | 358 | -7.98e-03 | 3.20e-03 | Uptake half saturation coefficient | Demersal fish larvae by carnivorous zooplankton |
| hsbenths_seal | 457 | -7.96e-03 | 3.83e-03 | Uptake half saturation coefficient | Suspension/deposit feeding benthos by pinnipeds |
| ufishdlar_carnt | 357 | 7.34e-03 | 3.38e-03 | Maximum uptake rate | Demersal fish larvae by carnivorous zooplankton |
| ufishplar_fishdt | 401 | 7.26e-03 | 3.77e-03 | Maximum uptake rate | Planktivorous fish larvae by demersal fish |
| xxceta | 567 | -7.25e-03 | 4.08e-03 | Density dependent mortality coefficient | Cetaceans |
| ucorp_bird | 451 | 7.10e-03 | 3.71e-03 | Maximum uptake rate | Corpses by birds |
| hsfishplar_fishd | 402 | -6.77e-03 | 4.22e-03 | Uptake half saturation coefficient | Planktivorous fish larvae by demersal fish |
| eFdlart | 525 | 6.28e-03 | 3.78e-03 | Background metabolic rate coefficient | Demersal fish larvae |
| icesensceta | 624 | -5.70e-03 | 3.98e-03 | Feeding half saturation sensitivity to ice cover | Cetaceans |
| ucarn_bird | 437 | 5.08e-03 | 4.16e-03 | Maximum uptake rate | Carnivorous zooplankton by birds |
| fdriver_ceta_exit | 1080 | -4.80e-03 | 4.37e-03 | Emigration rate | Cetaceans |
| xxbear | 568 | 4.46e-03 | 2.31e-03 | Density dependent mortality coefficient | Maritime mammals |
| ufishplar_fishpt | 375 | 4.45e-03 | 4.05e-03 | Maximum uptake rate | Planktivorous fish larvae by planktivorous fish |
| abear | 517 | -4.17e-03 | 3.86e-03 | Assimilation efficiency | Maritime mammals |
| fdrivermfish_em | 1053 | 4.05e-03 | 3.77e-03 | Emigration rate | Migratory fish |
| hscorp_bird | 452 | -4.01e-03 | 3.69e-03 | Uptake half saturation coefficient | Corpses by birds |
| hsfishplar_fishp | 376 | -3.68e-03 | 4.37e-03 | Uptake half saturation coefficient | Planktivorous fish larvae by planktivorous fish |
| hscarn_bird | 438 | -3.57e-03 | 3.48e-03 | Uptake half saturation coefficient | Carnivorous zooplankton by birds&mammala |
| hssed_benths | 428 | -3.08e-03 | 3.57e-03 | Uptake half saturation coefficient | Sediment detritus by suspension/deposit feeding benthos |
| hsfishdlar_fishp | 374 | 2.55e-03 | 3.89e-03 | Uptake half saturation coefficient | Demersal fish larvae by planktivorous fish |
| ucarn_ceta | 475 | 2.43e-03 | 3.51e-03 | Maximum uptake rate | Carnivorous zooplankton by cetaceans |
| icesensbear | 625 | 2.40e-03 | 3.25e-03 | Feeding half saturation sensitivity to ice cover | Maritime mammals |
| ubenthc_seal | 458 | 2.38e-03 | 3.96e-03 | Maximum uptake rate | Carnivore/scavenge feeding benthos by pinnipeds |
| ufishd_ceta | 485 | 2.22e-03 | 2.98e-03 | Maximum uptake rate | Demersal fish by cetaceans |
| uomni_ceta | 473 | 2.13e-03 | 2.79e-03 | Maximum uptake rate | Omnivorous zooplankton by cetaceans |
| ubird_bear | 496 | -2.01e-03 | 3.47e-03 | Maximum uptake rate | Birds by maritime mammals |
| hsfishdlar_fishd | 404 | -1.94e-03 | 3.90e-03 | Uptake half saturation coefficient | demersal fish larvae by demersal fish |
| ufishdlar_fishdt | 403 | 1.88e-03 | 3.72e-03 | Maximum uptake rate | demersal fish larvae by demersal fish |
| ufishdlar_fishpt | 373 | -1.84e-03 | 3.99e-03 | Maximum uptake rate | Demersal fish larvae by planktivorous fish |
| hsbenthslar_fishdlar | 392 | 1.51e-03 | 3.36e-03 | Uptake half saturation coefficient | Suspension/deposit feeding benthos larvae by demersal fish larvae |
| Phytoplankton net primary production - Sourced for the East Greenland Shelf | | | | | |
| fdriversslight | 1001 | 4.93e+02 | 1.16e+01 | Sea surface irradiance | Inshore and offshore zones |
| thik_so | 1 | -4.84e+02 | 2.67e+01 | Vertical thickness | Offshore zone upper layer |
| fdriverso_icecov | 1056 | -4.34e+02 | 1.44e+01 | Ice cover proportion in ice affected area | Offshore zone |
| fdriverboundso_phyt | 1027 | -3.18e+02 | 5.71e+00 | Boundary concentration | Upper layer offshore phytoplankton |
| lightSPM_slope | 54 | -2.92e+02 | 1.73e+01 | Coefficient | Light attenuation coefficient vs SPM |
| thik_si | 3 | -2.48e+02 | 9.38e+00 | Vertical thickness | Inshore zone |
| fdriverso_logespm | 1002 | -2.15e+02 | 1.22e+01 | Suspended particulate matter | Offshore zone |
| fdriverso_inflow | 1008 | -2.09e+02 | 2.79e+00 | Boundary volume inflow rate | Inshore zone |
| lightSPM_intercept | 53 | -2.01e+02 | 8.76e+00 | Intercept | Light attenuation coefficient vs SPM |
| fdriverboundso_nit | 1024 | 2.01e+02 | 7.47e+00 | Boundary concentration | Upper layer offshore nitrate |
| fdriversi_icecov | 1067 | -1.58e+02 | 1.13e+01 | Ice cover proportion in ice affected area | Inshore zone |
| inshore_phyt_prop_depth | 55 | -1.08e+02 | 1.10e+01 | Proportion of depth range occupied | Phytoplankton inshore |
| fdriversi_logespm | 1003 | -7.70e+01 | 5.77e+00 | Suspended particulate matter | Inshore zone |
| Reflect | 58 | -7.50e+01 | 2.26e+00 | Proportion of incoming light reflected by ice or snow | Ice and snow |
| fdriverboundso_det | 1018 | 5.71e+01 | 1.34e+00 | Boundary concentration | Upper layer offshore detritus |
| fdriverso_icefree | 1055 | 5.09e+01 | 1.15e+00 | Ice-free area proportion | Offshore zone |
| thik_d | 2 | -4.63e+01 | 1.68e+00 | Vertical thickness | Offshore zone lower layer |
| thik_b | 4 | 3.10e+01 | 9.53e-01 | Vertical thickness | Benthic boundary feeding layer |
| Kice | 60 | -2.87e+01 | 1.85e+00 | Light attenuation coefficient of ice | Sea-ice |
| fdriverso_icethick | 1057 | -2.41e+01 | 1.71e+00 | Ice thickness | Offshore zone |
| fdriverso_temp | 1004 | -1.54e+01 | 6.38e-01 | Temperature | Offshore zone upper layer |
| fdriversi_icefree | 1066 | 1.35e+01 | 4.06e-01 | Ice-free area proportion | Inshore zone |
| fdriversi_outflow | 1013 | -1.21e+01 | 2.98e-01 | Volume outflow rate | Inshore zone |
| fdriverv_dif | 1007 | 1.18e+01 | 1.99e-01 | Vertical diffusion rate | Offshore zone |
| fdriverboundd_nit | 1025 | 9.92e+00 | 2.56e-01 | Boundary concentration | Lower layer nitrate |
| fdriverboundso_amm | 1021 | 8.64e+00 | 1.66e-01 | Boundary concentration | Upper layer offshore ammonia |
| fdriverso_si_flow | 1014 | -8.26e+00 | 8.93e-01 | Volume exchange rate | Offshore to inshore zone |
| Ksnow | 59 | -7.69e+00 | 5.25e-01 | Light attenuation coefficient of snow | Snow |
| fdriverso_snowthick | 1058 | -6.24e+00 | 4.27e-01 | Snow thickness | Offshore zone |
| fdriversi_icethick | 1068 | -4.85e+00 | 2.37e-01 | Ice thickness | Inshore zone |
| fdriverboundsi_nit | 1026 | 3.68e+00 | 1.07e-01 | Boundary concentration | Inshore nitrate |
| fdriverd_inflow | 1009 | -2.56e+00 | 9.36e-02 | Boundary volume inflow rate | Lower layer offshore |
| fdriverboundd_det | 1019 | -1.45e+00 | 1.08e-01 | Boundary concentration | Lower layer detritus |
| fdriversi_snowthick | 1069 | -1.44e+00 | 1.10e-01 | Snow thickness | Inshore zone |
| fdriversi_temp | 1006 | -1.30e+00 | 1.63e-01 | Temperature | Inshore zone |
| fdriversi_inflow | 1010 | 1.23e+00 | 6.72e-02 | Boundary volume inflow rate | Inshore zone |
| fdriverd_temp | 1005 | 1.10e+00 | 4.66e-02 | Temperature | Lower layer offshore |
| fdriverboundsi_phyt | 1029 | -1.06e+00 | 4.46e-02 | Boundary concentration | Inshore phytoplankton |
| porosity_s1 | 28 | -6.86e-01 | 1.84e-02 | Sediment porosity | Inshore muddy sediments |
| fdriverboundd_phyt | 1028 | 5.77e-01 | 5.75e-02 | Boundary concentration | Lower layer phytoplankton |
| fdriverboundsi_det | 1020 | 4.62e-01 | 9.95e-03 | Boundary concentration | Inshore detritus |
| Poricez | 57 | 2.59e-01 | 1.35e-02 | Maximum thickness of the porous ice layer | Sea-ice |
| sed_wat_dif_s1 | 34 | -2.05e-01 | 1.33e-02 | Hydraulic conductivity | Inshore muddy sediments |
| fdriverboundsi_amm | 1023 | 1.41e-01 | 5.80e-03 | Boundary concentration | Inshore ammonia |
| porosity_s2 | 29 | -1.08e-01 | 4.92e-03 | Sediment porosity | Inshore sandy sediments |
| fdriverboundd_amm | 1022 | 1.04e-01 | 5.08e-03 | Boundary concentration | Lower layer ammonia |
| fdriverboundriv_nit | 1031 | 4.62e-02 | 4.39e-03 | Boundary concentration | River nitrate |
| porosity_s3 | 30 | -3.00e-02 | 3.99e-03 | Sediment porosity | Inshore coarse sediments |
| fdriverso_atm_nit | 1034 | 1.57e-02 | 3.41e-03 | Atmospheric deposition rate | Offshore nitrate |
| fdriverso_atm_amm | 1033 | 1.52e-02 | 2.76e-03 | Atmospheric deposition rate | Offshore ammonia |
| fdriversi_atm_nit | 1036 | 1.24e-02 | 3.98e-03 | Atmospheric deposition rate | Inshore nitrate |
| sed_wat_dif_s2 | 35 | -1.08e-02 | 3.92e-03 | Hydraulic conductivity | Inshore sandy sediments |
| extrusion | 61 | -1.05e-02 | 3.89e-03 | Temperature coefficinet for nitrate extrusion from ice | Sea-ice |
| porosity_d1 | 31 | -9.49e-03 | 3.10e-03 | Sediment porosity | Offshore muddy sediments |
| fdriversi_atm_amm | 1035 | 8.72e-03 | 3.49e-03 | Atmospheric deposition rate | Inshore ammonia |
| fdriverboundriv_amm | 1030 | 7.33e-03 | 3.53e-03 | Boundary concentration | River ammonia |
| sed_wat_dif_d1 | 37 | -5.69e-03 | 3.52e-03 | Hydraulic conductivity | Offshore muddy sediments |
| porosity_d2 | 32 | -4.69e-03 | 4.20e-03 | Sediment porosity | Offshore sandy sediments |
| porosity_d3 | 33 | -2.87e-03 | 3.52e-03 | Sediment porosity | Offshore coarse sediments |
| fdriverso_airtemp | 1054 | 2.50e-03 | 2.93e-03 | Air temperature | Offshore zone |
| fdriversi_airtemp | 1065 | 2.09e-03 | 3.41e-03 | Air temperature | Inshore zone |


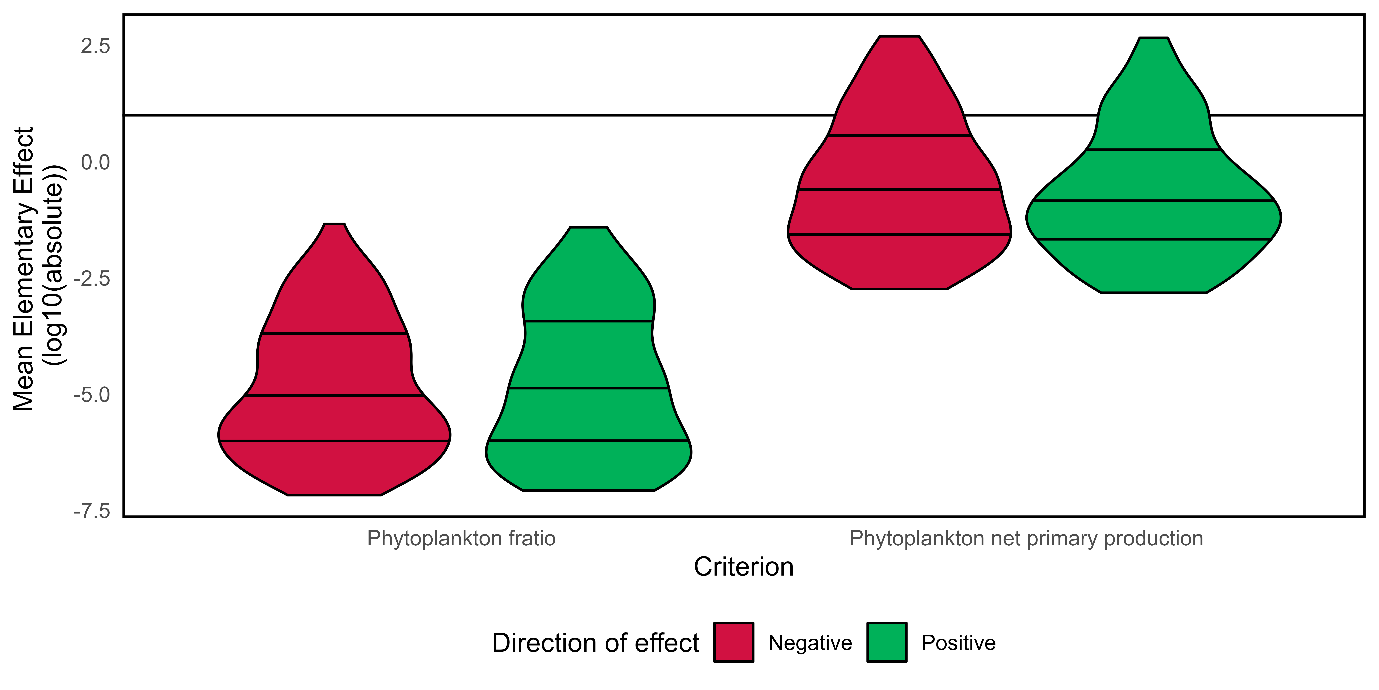


**SIFig 6: Distribution of mean elementary effects of statistically significant parameters borrowed from the Barents Sea implementation.** The horizontal black line marks an elementary effect of 1. Horizontal lines in violins indicate the 25^th^, 50^th^, and 75^th^ quantiles of either positive or negative effects. The y axis shows the absolute effect size to permit the log-scaling of negative effect sizes.
