# Supplementary material for: Sea‐Ice Retreat From the Northeast Greenland Continental Shelf Triggers a Marine Trophic Cascade
Source: Glob Chang Biol. 2025 Apr 24;31(4):e70189. doi: 10.1111/gcb.70189 (PMC12019585; doi:10.1111/gcb.70189)

STRATHE2EPOLAR

Northeast Greenland Shelf Implementation

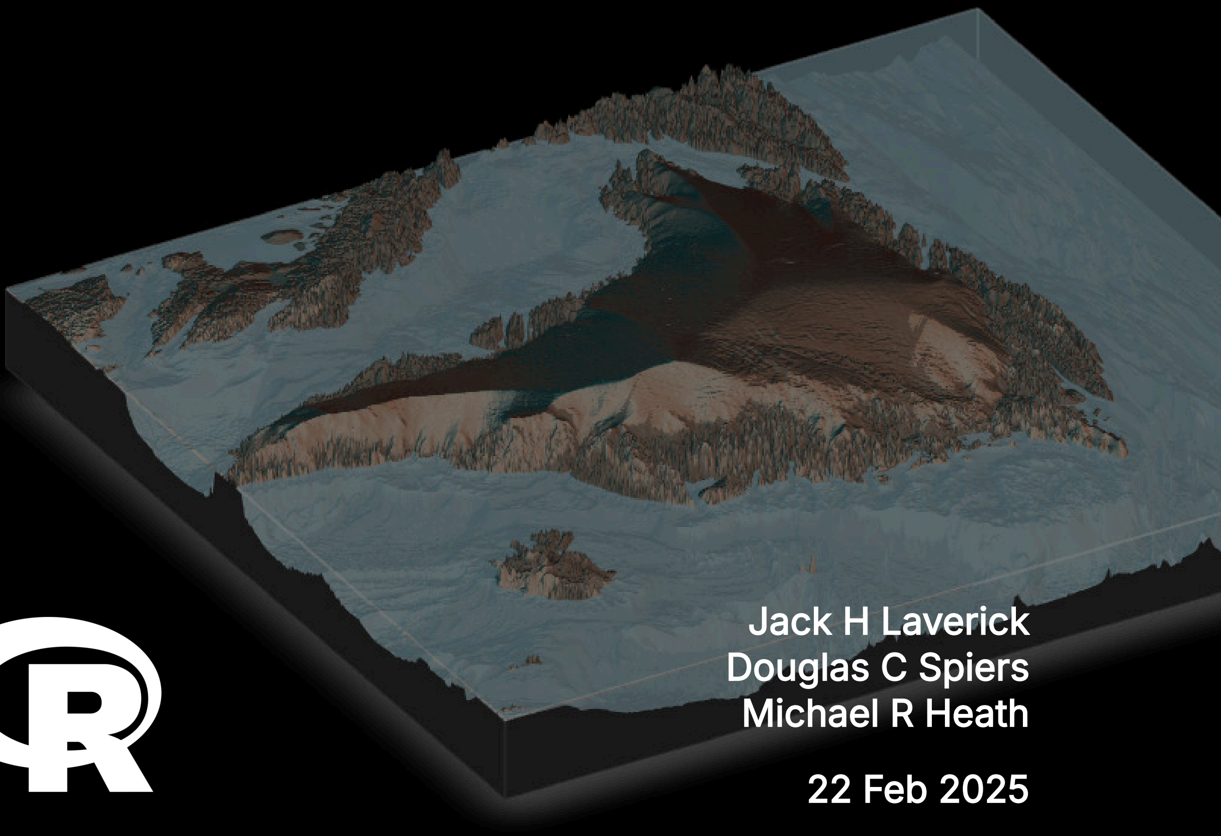

Jack H Laverick
Douglas C Spiers
Michael R Heath

22 Feb 2025

Introduction . . . 3
Model Domain . . . 4
Fixed Physical . . . 7
  Background . . . 7
  Model area proportions . . . 8
  Sediment porosity . . . 10
  Hydraulic conductivity . . . 11
  Sediment organic nitrogen content . . . 11
Fixed biological . . . 13
  Configuration parameters . . . 13
  Event timing parameters (not fitted) . . . 14
  Event timing parameters (fitted) . . . 15
Ecological drivers . . . 18
Fishing fleet . . . 24
  Background . . . 24
End . . . 25
  Acknowledgements . . . 25
References . . . 26

Introduction

This document describes the configuration of StrathE2E*polar* for the Northeast Greenland Shelf and its parameterisation to enable stationary state fitting for both the baseline period (2010-2019) and future projections (2020-2059). These represent contrasting periods of environmental conditions. Due to a lack of data on the Northeast Greenland Ecosystem, this implementation starts as an implementation for the Barents Sea. Parameters were updated as and when available for Northeast Greenland, described in this document.

Volumetric and seabed habitat data define the physical configuration of the system. We regard these as being fixed in time. Similarly, we regard the physiological parameters of the ecology model as being fixed in time. Some of these are set from external data. The remainder are fitted, as detailed here. Changes in the model performance between the different time periods therefore stem from the hydrodynamic, hydro-chemical and fishery driving data. These are detailed in the ecological drivers and fishing fleet sections.

Department of Mathematics and Statistics, University of Strathclyde, Glasgow, UK.

E-mail: m.heath@strath.ac.uk

The code written to support this parameterisation is available on github.

Model Domain

The model splits the domain into three zones, inshore/shallow, offshore/shallow, and offshore/deep (Figure 1). The inshore/shallow zone covers waters shallower than 60m or 20km from shore. The offshore zone covers the remaining area of the model domain (Figure 2). The offshore zone is divided further into a shallow and deep layer. The shallow layer represents water from the surface to 60m depth, and shares a boundary with the inshore shallow zone. The offshore/deep zone covers the same area as the offshore/shallow zone, but represents water between 60m and 400m deep. There is a second internal boundary between the two offshore zones.

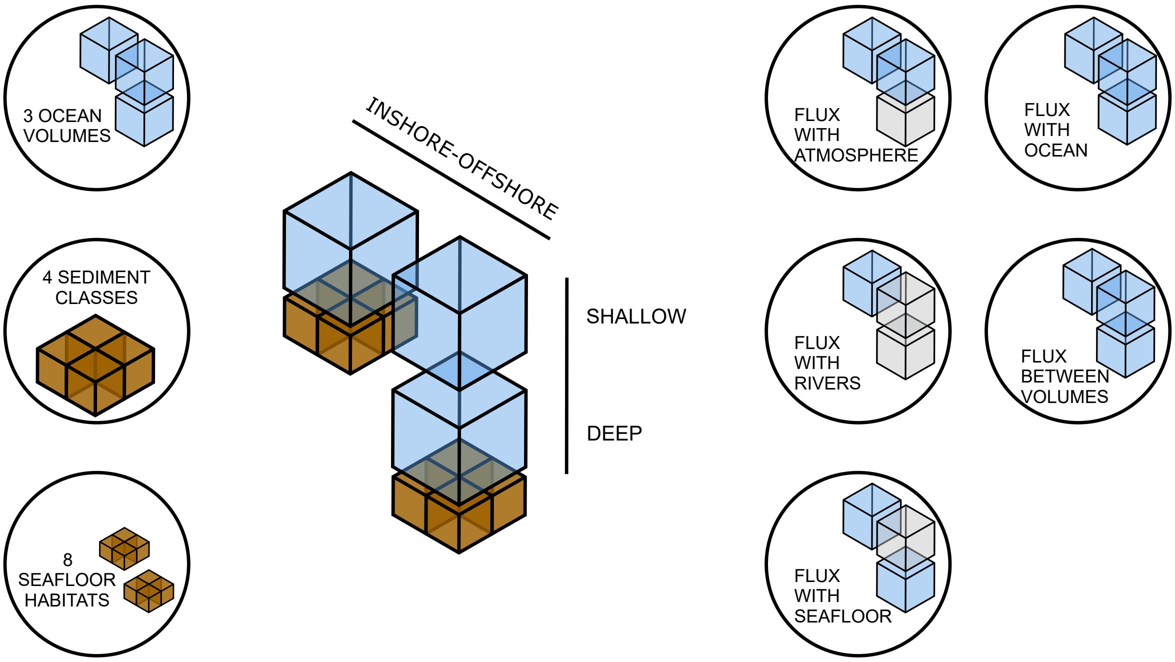

The spatial structure of StrathE2E; Ocean volumes and seafloor habitats. StrathE2E is built around a simplified spatial structure which represents shelf seas. These spatial units are connected to each other and to boundaries as shown to the right. The volumes connected to each spatial component are highlighted in blue.

The seafloor of the model domain is represented by 8 habitat types. There are three sediment classes – fine (muddy, 1), medium (sandy, 2) and coarse (gravel, 3). The fourth class (rock, 0) represents an absence of soft sediment. These sediment classes are defined in both the inshore/shallow and offshore/deep zones, yielding 8 habitats (Figure 2). The rock class has different geochemical properties.

The perimeter of the model domain is defined by a 400m depth contour and the coastline of Greenland. Open ocean boundaries occur wherever there is no coastline. We imposed additional boundaries to limit the northward extent of the model domain at 81.5N and Southward at 70N where the shelf constricts.

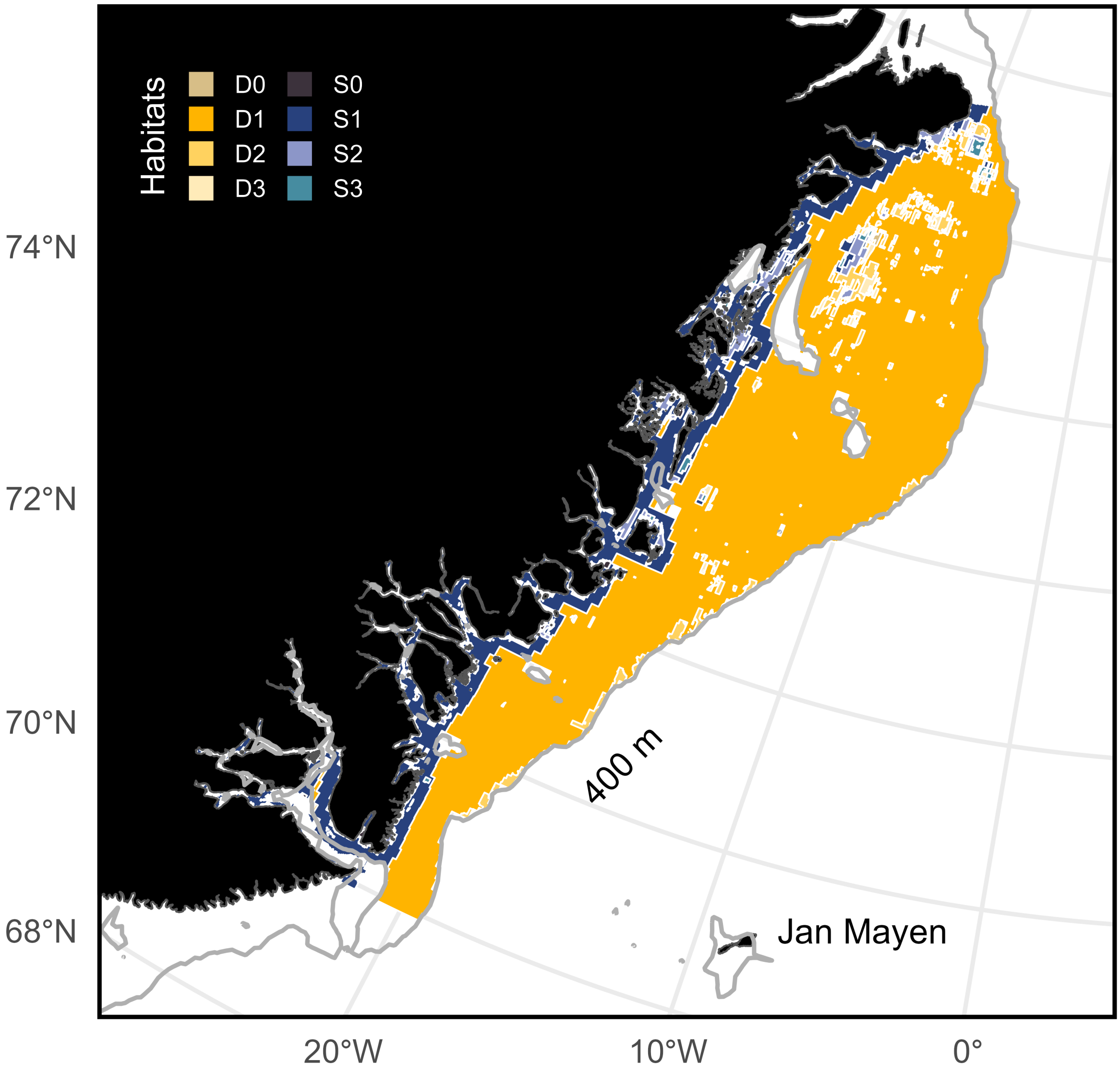

Map of the model domain. StrathE2E defines seabed sediment habitats as inshore (blues) or offshore (yellows). Within each zone, three sediment classes are represented – fine (muddy,

1), medium (sandy, 2), and coarse (gravel, 3). A fourth class (rock, 0) represents an absence of soft sediment. Sedimentary data are from Laverick et al. (2023).

Fixed Physical

Background

Water column inshore/shallow and offshore/deep zone area proportions and layer thicknesses; seabed habitat area proportions and sediment properties:

Area proportions of depth zones and seabed habitats derived from 1/100th degree resolution atlas of seabed sediment properties (Laverick, Speirs, and Heath 2023). The atlas provides gridded data sets of bathymetry, mean grain size, mud, sand and gravel content, porosity, permeability, organic nitrogen and carbon content, and natural disturbance by waves and bed shear stress.

Parameters for relationship between median grain size, sediment porosity and permeability. Permeability is used as the basis for estimating hydraulic conductivity which is a parameter in the representation of sediment processes in the model:

Porosity (proportion by volume of interstitial water) and permeability of each sediment habitat were derived from median grain sizes using empirically-based relationships.

$$log_{10}(porosity) = p_3 + p_4 \left(\frac{1}{1 + e^{\left(\frac{-log_{10}(D_{50}) - p_1}{p_2}\right)}} \right)$$

D_{50} = median grain size (mm); parameters p_1 = -1.227, p_2 = -0.270, p_3 = -0.436, p_4 = 0.366 (Heath et al. 2021)

$$permeability = 10^{p_5} \bullet D_{50}^{*p_6}$$

where D_{50}^* = 0.11 ≤ D_{50} ≤ 0.50 p_5 = -9.213, p_6 = 4.615 (Heath, Wilson, and Speirs 2015).

These relationships are coded into the StrathE2E2 R-package with the parameters in the csv setup file for the North Sea model. The

parameters are probably a reasonable starting point for any future model of a new region. Derivation of the parameters is described in the following text sub-sections.

Parameters for in-built relationship between sediment mud content, and slowly degrading (refractory) organic nitrogen content of seabed sediments (see description in this document):

Values for each sediment type derived from parameterised relationships between total organic nitrogen content of sediments (TON%, percent by weight), mud content (mud%, percent by weight) and median grain size (D_{50}, mm).

$$mud\% = 10^{p_7} \bullet D_{50}^{p_8}$$

p_7 = 0.657, p_8 = -0.800

$$TON\% = 10^{p_9} \bullet mud\%^{p_{10}}$$

p_9 = -1.965, p_{10} = 0.590

Proportion of TON estimated to be refractory = 0.9

These relationships, along with their parameters, are documented in the North Sea implementation of the StrathE2E2 package (Heath et al. 2021). While there may be regional variations, these relationships serve as a reasonable starting point for this implementation. The StrathE2E2 R-package encodes these relationships, with the parameters specified in the CSV setup file. Derivation of the parameters is described in the following sub-sections.

Model area proportions

Table 1: Area-proportions of the inshore and offshore zones and the thicknesses of the water column layers. The sea surface area of the model domain is an estimated 279041.398538012 km^2.

Property	Inshore/shallow	Offshore/deep
Sea-surface area proportion	0.2226	0.7774
Upper layer thickness (m)	171.0385	60.0000
Lower layer thickness (m)	NA	178.4672

We derived the area-proportions of seabed habitat in the inshore and offshore zones from the atlas of seabed sediment properties from Laverick et al. (2023). The atlas provides a range of seabed data for 1/100th degree cells over the Barents and Greenland Seas, including the presence of rock, the percentage of mud, sand and gravel fractions in the sediments, the whole–sediment mean grain size, and the natural disturbance rate by currents and waves. These values are derived from habitat classes used by the Norwegian Geological survey in partnership with the Russian Federal State Unitarian Research and Production Company for Geological Sea Survey (NGU-SEVMORGEO). We assigned the NGU-SEVMORGEO sediment classes as fine, medium, coarse, or absence of sediment habitats within each zone (Figure 2). The actual area of each habitat was then the sum of the areas of each set of assigned cells (Table 2, Figure 2).

Table 2: Area proportions and other characteristics of the 8 seabed habitat classes defined in the model by depth, rock or sediment type. The sea surface area of the model domain is an estimated 279041.398538012 km^2. Grain size is the median in mm, Permeability in units of m^2, nitrogen content in %dw.

Habitat	Sediment	Area Proportion	Grain size	Porosity	Permeability	Nitrogen content
Inshore - Shallow						
S0	None (Rock)	0.0055	0.0000	NA	NA	NA
S1	Fine	0.1885	0.0278	0.6794	0.0000	0.1250
S2	Medium	0.0233	0.2159	0.4582	0.0000	0.0406
S3	Coarse	0.0054	4.3806	0.3808	0.0000	0.0297
Offshore - Deep						
D0	None (Rock)	0.0010	0.0000	NA	NA	NA
D1	Fine	0.7350	0.0215	0.6847	0.0000	0.1358
D2	Medium	0.0322	0.2854	0.4460	0.0000	0.0399
D3	Coarse	0.0091	1.9996	0.3879	0.0000	0.0338

Sediment porosity

Log-transformed porosity has been shown to have a sigmoidal relationship with $\log_{10}$(median grain size) (D_{50}, mm) (Wilson et al. 2018):

c

We use this relationship to calculate porosity for sea bed sediments on the Greenland Shelf (Table 2), using an alternative parameterisation to Wilson (Pace et al. 2021). This alternative set of parameters extends the relationship to fine, muddy sediments (Table 3).

Table 3: The four parameters for the function relating sediment porosity to median grain size. From Pace et al. (in review)

P1	P2	P3	P4
-1.035	-0.314	-0.435	0.302

Hydraulic conductivity

Hydraulic conductivity (H, m.s^{-1}) represents the ease with which fluids flow through the particle grain matrix. The related term 'permeability' (m^{-2}) is a measure of the connectedness of the fluid filled void spaces between the particle grains. Permeability is a function only of the sediment matrix, whilst conductivity is a function of both the sediment and the permeating fluid, in particular the fluid viscosity and density. Hydraulic conductivity is related to permeability by:

$$H = Permeability \bullet fluid\ density \bullet \frac{g}{dynamic\ viscosity}$$

where: seawater density = 1027 kg.m^{-3} at salinity 35 and temperature 10°C; seawater dynamic viscosity = 1.48 x 10^{-3} kg.m^{-1}.s^{-1} at salinity 35 and temperature 10°C; g = acceleration due to gravity = 9.8 m.s^{-1}

Hence, $H = Permeability \cdot 6.8004 \cdot 10^6$ (m.s^{-1} at salinity 35 and temperature 10°C)

Whole sediment permeability can be related to the proportion of sediment classed as mud (D_{50} < 62 μm) (Pace et al. 2021). In this model implementation, this relationship is utilized for internal calculations.

Sediment organic nitrogen content

The magnitude of the static (refactory) organic nitrogen detritus pool in each sediment type is a required input to the model. The code includes an option to impute values from empirical relationships between total organic nitrogen (TON) and mud content, and between mud content and median grain size. This relationship has been documented in the North Sea implementation of the temperate

StrathE2E2 package (Heath et al. 2021), and is based on sediment data off northeast Scotland.

To supplement the relationship within the package, predictions of sediment organic nitrogen content for the Barents Sea have been sourced from the sediment atlas (Laverick, Speirs, and Heath 2023) to parameterise StrathE2E directly (Table 2).

Fixed biological

Configuration parameters

Assimilation efficiencies for each living guild in the model.

Fixed parameters defining the proportion of ingested mass of food that contributes to new body tissue, after subtracting defecation and the metabolic costs of digestion and synthesis (Heath 2012).

Biomass loss rates due to temperature-dependent metabolism for each living resource guild.

Proportion of biomass lost to ammonia per day due to non-feeding related metabolism at a given reference temperature. Rates for individual guilds broadly related to typical body mass of representative species. Temperature dependency following a Q_{10} function.

Q_{10} values for temperature dependent processes, and the Q_{10} reference temperature.

Separate Q10 values for autotrophic uptake of nutrient, heterotrophic feeding, and heterotrophic metabolism based on literature data.

Light intensity required to saturate autotrophic nutrient uptake.

Light saturation intensity for nutrient uptake cannot be treated as a fitted value since it is confounded with other uptake parameters. Value estimated from survey of laboratory experiments.

Annual weight specific fecundities of planktivorous and demersal fish guilds and the two benthos guilds in the model (suspension/deposit feeders and carnivore/scavenge feeders).

Guild-level values derived by surveying the literature.

Harvestable biomass density threshold for each resource guild.

The living resource guilds in the model represent a mixture of harvestable and non-harvestable species, especially the invertebrate guilds. The density threshold parameter sets a limit for the guild biomass below which the harvestable species are assumed to be exhausted. Values set from analysis of trawl, plankton and benthos survey species biomass compositions.

Minimum inedible biomass of carnivorous zooplankton.

The carnivorous zooplankton guild is a key component of the food web, predated on by all the fish and top-predators. However it represents an extremely diverse range of fauna many of which are not edible in significant quantities by the guild predators, e.g. scyphomedusae. A minimum edible threshold is set to ensure that the guild as a whole cannot be extirpated by predation. The value is a rough estimate of scyphomedusae biomass.

Event timing parameters (not fitted)

In the absence of detailed information these parameters are borrowed from the Barents Sea implementation.

Spawning start and end dates for fish and benthos

For the fish guilds the dates were obtained from literature survey (Heath 2012), while others came from ecological surveys in Hornsund fjord on southern Spitsbergen (WĘsławski et al. 1988). The annual weight-specific fecundity is assumed to be shed uniformly between the start and end dates of spawning.

Recruitment start and end dates for fish and benthos

Obtained from literature survey (Heath 2012). The annual cohort of larvae/juveniles of each fish and benthos guild is assumed to recruit to the settled stage at a uniform daily rate between the start and end dates.

Extra-domain stock biomass of migratory, and the proportion invading the domain each year. Start and end dates for the annual invasion, and start and end dates for the emigration. (see description below).

The main migratory fish species undertaking a seasonal transit of the North Sea is the Atlantic mackerel. Data on the North East Atlantic stock biomass, the proportion entering the Barents Sea and the timing of the migration, were derived from stock assessment literature (ICES 2013).

Event timing parameters (fitted)

Migratory fish in the Barents Sea model are assumed to be Atlantic mackerel. The fishery for Atlantic mackerel is one of the most valuable in the northeast Atlantic. Spawning takes place off southwest Ireland in April. After spawning, fish rapidly migrate to summer feeding zones thousands of kilometres northwards along the continental shelf edge to the Norwegian and Barents Seas. More recently some fish feed off Iceland (Holst, Jansen, and Slotte 2016).

For the purposes of the model, we assume that there is no feedback between fishing and environmental conditions in the Barents Sea and the biomass and migration patterns of the whole northeast Atlantic mackerel stock. In this version of StrathE2E*polar* the timing of immigration and emigration, and the mass influx across the ocean boundary during the annual immigration phase are treated as period-specific external driving data.

Data on the ‘global’ stock of northeast Atlantic mackerel (wet biomass) are available from stock assessments (ICES 2013), and converted to molar nitrogen mass using appropriate conversion ratios (Greenstreet 1996). The proportion of the migrating stock entering the Barents Sea, and the timing of the inward and outward migrations are estimated from monthly resolved data on the spatial distribution of fishery catches. A residual proportion of the peak

abundance in the North Sea remaining as residents (if any) is estimated from summer trawl survey data. The model setup code calculates the parameters which are needed in the ecology model.These are the only fixed (i.e. non-fitted) ecology model parameters which are period-specific.

In addition to migratory fish, birds and cetaceans also migrate to the Barents Sea to feed during the summer, and leave during the winter. Polar bears similarly leave the model area during winter to hibernate. A constant rate of loss is applied to the hibernating guild while outside the model, in contrast to migratory guilds which continue to feed elsewhere. The values used for the timings of these events represent a synthesis of anecdotal reports.

Table 4: Biological event timing parameters, constant across the time periods. The data are processed in the model setup to calculate the immigration flux parameters needed in the ecology model.

Parameter	Value
Planktivorous fish spawning start day	60
Planktivorous fish spawning duration (days)	90
Planktivorous fish recruitment start day	200
Planktivorous fish recruitment duration (days)	150
Demersal fish spawning start day	60
Demersal fish spawning duration (days)	90
Demersal fish recruitment start day	200
Demersal fish recruitment duration (days)	150
Susp/dep benthos spawning start day	60
Susp/dep benthos spawning duration (days)	150
Susp/dep benthos recruitment start day	180
Susp/dep benthos recruitment duration (days)	120
Carn/scav benthos spawning start day	60

Table 4 Continued.

Parameter	Value
Carn/scav benthos spawning duration (days)	150.000
Carn/scav benthos recruitment start day	180.000
Carn/scav benthos recruitment duration (days)	120.000
Migratory fish switch (0=off 1=on)	1.000
Migratory fish ocean biomass (Tonnes wet weight)	3800000.000
Migratory fish carbon to wet weight (g/g)	0.184
Model domain sea surface area (km2)	1608975.700
Propn of ocean population entering model domain each year	0.050
Migratory fish immigration start day	150.000
Migratory fish immigration end day (must be later than start day even if migration disabled)	195.000
Migratory fish propn of peak popn in model domain which remains and does not emigrate	0.010
Migratory fish emigration start day	240.000
Migratory fish emigration end day (must be later than start day even if migration disabled)	285.000

Ecological drivers

Monthly resolution time-varying physical and chemical driving parameters for the model were derived from a variety of sources:

- Temperature, cryosphere variables, vertical mixing coefficients, volume fluxes, and boundary nutrient, detritus and phytoplankton concentrations from outputs of a NEMO-MEDUSA coupled hydro-geochemical model run at RCP85 with a 2005/2006 historical/future split (Yool, Popova, and Anderson 2013).
- Surface shortwave radiation, surface air temperature, and freshwater volume outflows from HadGEM2-ES model output (Jones et al. 2011) used to force the NEMO-MEDUSA coupled hydro-geochemical model mentioned above (Yool, Popova, and Anderson 2013).
- River nitrate and ammonia concentrations derived from meltwater concentrations reported by Wadham et al (2016).
- Atmospheric deposition of nitrate and ammonia from EMEP MSC-W (European Monitoring and Evaluation Programme; (Simpson et al. 2003))
- Oceanic Nitrate and ammonia data from Changing Arctic Ocean programme (CAO) cruises in the Barents Sea, published by BODC (Brand, Norman, Mahaffey, et al. 2020; Brand, Henley, Mahaffey, et al. 2020a, 2020b; Brand, Norman, Henley, et al. 2020).
- Remote sensing data products on Suspended Particulate Matter (Globcolour L3b; ftp://ftp.hermes.acri.fr/GLOB/merged/month/).
- Habitat disturbance due to tidal currents and waves from the Barents Sea sediment atlas (Laverick, Speirs, and Heath 2023).
- Wave height, period, and direction from the CERA-20C 'Ocean Wave Synoptic Monthly Means' product accessed through ECMWF for 2000-2010.

Details of how these data were processed are given below, supported by the nemomedusR and MiMeMo.tools packages.

Vertical mixing coefficients between the upper and lower layers of the deep zone:

Vertical diffusivity from the NEMO-MEDUSA coupled hydro-geochemical model output (Yool, Popova, and Anderson 2013) was interpolated for each grid cell at the 60 m boundary depth between the shallow and deep layers of the offshore zone. These values were summarised as monthly averages into period-specific climatological annual cycles of data for the 2011-2019 and 2040-2049 simulation periods.

Monthly averaged temperatures and cryosphere variables for each water column layer:

Derived by monthly averaging values at grid points within the inshore and vertical layers of the offshore zones from the NEMO-MEDUSA coupled hydro-geochemical model output (Yool, Popova, and Anderson 2013), weighted by grid point volumes. Values were summarised into period-specific climatological annual cycles of data for the 2011-2019 and 2040-2049 periods.

Monthly averaged suspended particulate matter (SPM) concentrations (mg.m^{-3}) in the shallow zone and the deep zone upper layer:

Monthly averaged values of inorganic suspended particulate matter in sea water are available from the Globcolour project, starting from September 1997. These data are derived from satellite observations using the algorithm of Gohin (2011). Data were downloaded from the ftp server (ftp://ftp.hermes.acri.fr/GLOB/merged/month/). We summarised these values as zonal statistics for the model domain to acquire a climatological annual cycle of data for the 2011-2019 simulation period only.

Monthly average light attenuation coefficients for the inshore and offshore surface layers:

Light attenuation in open water was parameterised from a linear relationship between the light attenuation coefficient and suspended particulate matter concentration (SPM) (Devlin et al., 2008). Light attenuation and albedo for snow and ice were sourced from (Castellani et al. 2017).

Monthly averaged daily integrated irradiance at the sea surface ($E.m^{-2}.d^{-1}$):

Derived from HadGEM2-ES model output (Jones et al. 2011) which forces the NEMO-MEDUSA model run used throughout our implementation. Monthly mean values were summarised into a climatological annual cycle of data for both the 2011-2019 and 2040-2049 periods.

Monthly averaged daily atmospheric deposition rates of wet and dry, oxidised and reduced nitrogen onto the sea surface in the shallow and deep zones ($mMN.m^{-2}.d^{-1}$):

Sourced from 50 x 50 km^2 gridded data for 2000 - 2017 as monthly averages (Simpson et al. 2003), available from EMEP (https://thredds.met.no/thredds/fileServer/data/EMEP/2018_Reporting/). Monthly values were summarised into climatological annual cycles of monthly oxidised and reduced nitrogen deposition rates extracted for 2011-2017.

Monthly averaged, freshwater river inflow rates (expressed as a daily proportion of the receiving layer volume), and concentrations of oxidised and reduced dissolved inorganic nitrogen in the inflowing river waters ($mMN.m^{-3}$):

Freshwater inflow derived from HadGEM2-ES model output (Jones et al. 2011) which forces the NEMO-MEDUSA model run used throughout our implementation. Monthly values were summaries into a climatological annual cycle of data for both the 2011-2019 and 2040-2049 periods.

The closest estimates of the concentrations of oxidised and reduced dissolved inorganic nitrogen in river water to the model domain were from the Watson river (Wadham et al. 2016). We derived a climatological annual cycle of data for the 2011-2019 simulation period only using the following process.

We relied on data describing the course of a melt cycle in 2012 into the Watson river. After unsuccessful attempts to contact the authors, the data from figure 2 and supporting figure 5 were extracted using webplot digitizer. The resulting data were imported into R and summarised as the average daily flow rates and N concentrations to

smooth through any possible error. The digitisation process resulted in a small number of days with unpaired nutrient loads and freshwater volumes. These were filled by linear interpolation.

The data were used to fit a model for exponential decay of each nitrogen source against freshwater discharge.

$$NO_3|NH_4 = a * (1 - b)^{Discharge}$$

For NO_3 a = 3.46 (3sf, se = 0.142) and b = 0.00402 (3sf, se = 0.000444) and a residual standard error of 0.678. For NH_4 a = 0.578 (3sf, se = 0.0644) and b = 0.00163 (3sf, se = 0.000615) and a residual standard error of 0.3349.

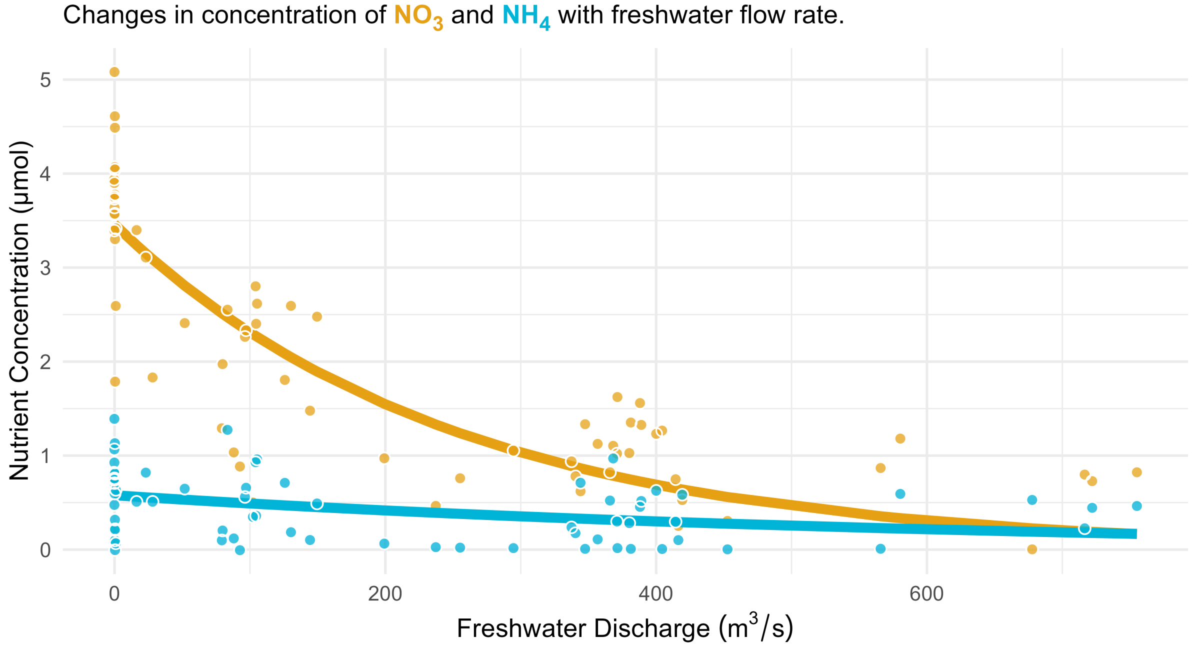

The fit of two exponential decay models to data on the relationship between freshwater discharge rates and concentrations of dissolved nitrogen species in the Watson river (Wadham et al. 2016).

To predict the N concentrations in the model domain using the model above and freshwater discharge forcing NEMO-MEDUSA, it is necessary to control for the catchment area.

Rignot and Mouginot (2012) report on the catchment area for glaciers larger than 1.5 km across in their supporting materials. The total catchment area for NE glaciers in their list is 358,395 km2. Wadham et al. describe their runoff station as being downstream of the Leverett glacier, however looking at their map (Figure 1) it is apparent the location of the discharge data is after two confluences connected to the catchment of the Russsell Glacier. Rignot and Mouginot list two glaciers with the correct coordinates that have

terminii upstream of the sampling station "Russell Gletscher" and "Unnamed south Russell". The relationship from above is therefore for a catchment area of 7,208 + 9,930 km2. This is 21 times smaller than the catchment area of the NE shelf (our model domain).

Dividing the NEMO-MEDUSA freshwater input by 21 scales the units for the NE shelf to match those used in the relationship derived from the Watson river. After this correction the flow rates from the model domain appear comparable to those from the field observations. This also allows us to use the freshwater projections to predict changes to future ammonia and nitrate concentrations. If we didn't control for this the amount of nitrogen added to the system would follow increasing snow melt without any chance for dilution.

Volume fluxes into the model domain across open sea boundaries, and from the upper layer of the offshore/deep zone into the inshore/shallow zone, expressed as proportions of the receiving layer volume per day:

Monthly averaged daily inflow and outflow volume fluxes derived by integrating daily mean velocities directed perpendicular to transects along the model domain boundary at grid points in each depth layer along transects through outputs from the NEMO-MEDUSA coupled hydro-geochemical model output (Yool, Popova, and Anderson 2013). Monthly averaged daily inflow volume fluxes then divided by the volume of the receiving layer in the model domain to estimate a daily flushing rate. Period-specific climatological annual cycles of data used for 2011-2019 and 2040-2049 simulation periods.

Mean concentrations of nitrate, ammonia, phytoplankton and suspended detritus ($mMN.m^{-3}$), in adjacent ocean waters inflowing to the offshore/deep zone upper layer, adjacent ocean waters inflowing to the offshore/deep zone lower layer, and adjacent shelf waters inflowing to the inshore/shallow zone:

NEMO-MEDUSA outputs included phytoplankton and suspended detritus, as well as Dissolved Inorganic Nitrogen (DIN). We calculated the depth-averaged concentrations for pixels within the shallow and deep layers of StrathE2E. We then sampled the pixels using the same transects around the model domain as for sampling volume fluxes. Only transects where water flowed into the model domain were sampled, and the average concentration of inflowing waters for target variables was calculated weighting by the flow rate across a

transect and the cross-sectional area represented by a transect (average depth and length). Concentrations were then averaged into climatological annual cycles for both the 2011-2019 and 2040-2049 periods.

DIN was decomposed into nitrate and ammonia concentrations using a ratio of ammonia:DIN derived from field observations collected during NERC Changing Arctic Ocean Cruises (Brand, Norman, Mahaffey, et al. 2020; Brand, Henley, Mahaffey, et al. 2020a, 2020b; Brand, Norman, Henley, et al. 2020). Concentrations were averaged by depth layer into two correction factors across all samples located in the model domain and across all time steps.

Fishing fleet

Background

Due to the high concentrations of sea ice there is little fishing reported within the present day model domain for the Northeast Greenland Shelf. We have therefore set fishing activity rates to 0, effectively switching off the fisheries sub-model, and leave all other parameters as defined for the Barents Sea. This allows for scenario experiments concerning how the ecosystem would respond to fisheries operating in a way described for the Barents Sea.

The key configuration data for the fishing fleet model are the definitions of the gears in terms of their power with respect to each of the harvestable resource guilds, discarding rates, processing-at-sea rates, and their seabed abrasion rates. These can be regarded as static parameters for each gear. Species were categorized into the commercial guilds of the model based on their ecological properties.

End

Acknowledgements

Financial support for the development of the Brazilian Shelf implementation of StrathE2E2 came from the European Union's Horizon 2020 research and innovation programme (Mission Atlantic - No. 862428). We are grateful to:

- Andy Yool and the National Oceanography Centre for making outputs from the NEMO-MEDUSA model available to us, from which we extracted driving data for StrathE2E2.

References

Brand, T., S. F. Henley, C. Mahaffey, K. C. Crocket, L. Norman, and R. Tuerena. 2020a. "Dissolved Nutrient Samples Collected in the Barents Sea as Part of the Changing Arctic Ocean Programme During Cruise JR17006, June-July 2018." British Oceanographic Data Centre. https://doi.org/doi:10/fncd.

———. 2020b. "Dissolved Nutrient Samples Collected in the Barents Sea as Part of the Changing Arctic Ocean Programme During Cruise JR17007, July-August 2018." British Oceanographic Data Centre. https://doi.org/doi:10/fndh.

Brand, T., L. Norman, S. F. Henley, C. Mahaffey, K. C. Crocket, and R. Tuerena. 2020. "Dissolved Nutrient Samples Collected in the Barents Sea as Part of the Changing Arctic Ocean Programme During Cruise JR18006, July-August 2019." British Oceanographic Data Centre. https://doi.org/doi:10/fndf.

Brand, T., L. Norman, C. Mahaffey, R. Tuerena, K. C. Crocket, and Henley S. F. 2020. "Dissolved Nutrient Samples Collected in the Fram Strait as Part of the Changing Arctic Ocean Programme During Cruise JR17005, May-June 2018." British Oceanographic Data Centre. https://doi.org/doi:10/fnb6.

Castellani, Giulia, Martin Losch, Benjamin A. Lange, and Hauke Flores. 2017. "Modeling Arctic Sea-Ice Algae: Physical Drivers of Spatial Distribution and Algae Phenology." *Journal of Geophysical Research: Oceans* 122 (9): 7466–87. https://doi.org/https://doi.org/10.1002/2017JC012828.

Gohin, F. 2011. "Annual Cycles of Chlorophyll-a, Non-Algal Suspended Particulate Matter, and Turbidity Observed from Space and in-Situ in Coastal Waters." *Ocean Science* 7: 705–32.

Greenstreet, S. P. R. 1996. "Scottish Fisheries Research Report: Estimation of the Daily Consumption of Food by Fish in the North Sea in Each Quarter of the Year." Vol. 55. The Scottish Office Agriculture, Environment; Fisheries Department.

Heath, M. R. 2012. “Ecosystem Limits to Food Web Fluxes and Fisheries Yields in the North Sea Simulated with an End-to-End Food Web Model.” *Progress in Oceanography* Special issue: End-to-end modelling: Towards Comparative Analysis of Marine Ecosystem Organisation (102): 42–66.

Heath, M. R., D. C. Speirs, I. Thurlbeck, and R. J. Wilson. 2021. “StrathE2E2: An r Package for Modelling the Dynamics of Marine Food Webs and Fisheries.” *Methods in Ecology and Evolution* 12 (2): 280–87. https://doi.org/https://doi.org/10.1111/2041-210X.13510.

Heath, M. R., R. Wilson, and D. C. Speirs. 2015. “Modelling the Whole-Ecosystem Impacts of Trawling. A Study Commissioned by Fisheries Innovation Scotland (FIS).” FIS.

Holst, J. C., T. Jansen, and A. Slotte. 2016. “Quantifying Changes in Abundance, Biomass, and Spatial Distribution of Northeast Atlantic Mackerel (Scomber Scombrus) in the Nordic Seas from 2007 to 2014.” *ICES Journal of Marine Science* 73: 359–73.

ICES. 2013. “Mackerel in the Northeast Atlantic (Combined Southern, Western, and North Sea Spawning Components).” In *International Council for the Exploration of the Sea Advice Book*, 17. 9. ICES.

Jones, C. D., J. K. Hughes, N. Bellouin, S. C. Hardiman, G. S. Jones, J. Knight, S. Liddicoat, et al. 2011. “The HadGEM2-ES Implementation of CMIP5 Centennial Simulations.” *Geoscientific Model Development* 4 (3): 543–70. https://doi.org/10.5194/gmd-4-543-2011.

Laverick, Jack H., Douglas C. Speirs, and Michael R. Heath. 2023. “Synthetic Shelf Sediment Maps for the Greenland Sea and Barents Sea.” *Geoscience Data Journal* 10 (2): 220–30. https://doi.org/https://doi.org/10.1002/gdj3.154.

Pace, M. C., D. M. Bailey, D. W. Donnan, B. E. Narayanaswamy, H. J. Smith, D. C. Speirs, W. R. Turrell, and M. R. Heath. 2021. “Modelling Seabed Sediment Physical Properties and Organic Matter Content in the Firth of Clyde.” *Earth System Science Data Discussions* 2021: 1–35. https://doi.org/10.5194/essd-2021-23.

Rignot, E., and J. Mouginot. 2012. “Ice Flow in Greenland for the International Polar Year 2008–2009.” *Geophysical Research Letters* 39 (11). https://doi.org/https://doi.org/10.1029/2012GL051634.

Simpson, D., H. J. Fagerli, J. E. Jonson, S. Tsyro, P. Wind, and J. P. Tuovinen. 2003. “EMEP Status Report: Unified EMEP Model

Description, Transboundary Acidification, Eutrophication and Ground Level Ozone in Europe." 1. Vol. 1/2003. Norwegian Meteorological Institute.

Wadham, J. L., J. Hawkings, J. Telling, D. Chandler, J. Alcock, E. O'Donnell, P. Kaur, et al. 2016. "Sources, Cycling and Export of Nitrogen on the Greenland Ice Sheet." *Biogeosciences* 13 (22): 6339–52. https://doi.org/10.5194/bg-13-6339-2016.

WĘsławski, J. M., M. Zajaczkowski, S. Kwaśniewski, J. Jezierski, and W. Moskal. 1988. "Seasonality in an Arctic Fjord Ecosystem: Hornsund, Spitsbergen." *Polar Research* 6 (2): 185–89. https://doi.org/10.3402/polar.v6i2.6861.

Wilson, R. J., D. C. Speirs, A. Sabatino, and M. R. Heath. 2018. "A Synthetic Map of the North-West European Shelf Sedimentary Environment for Applications in Marine Science." *Earth System Science Data* 10 (1): 109–30. https://doi.org/10.5194/essd-10-109-2018.

Yool, A., E. E. Popova, and T. R. Anderson. 2013. "MEDUSA-2.0: An Intermediate Complexity Biogeochemical Model of the Marine Carbon Cycle for Climate Change and Ocean Acidification Studies." *Geoscientific Model Development* 6 (5): 1767–1811. https://doi.org/10.5194/gmd-6-1767-2013.

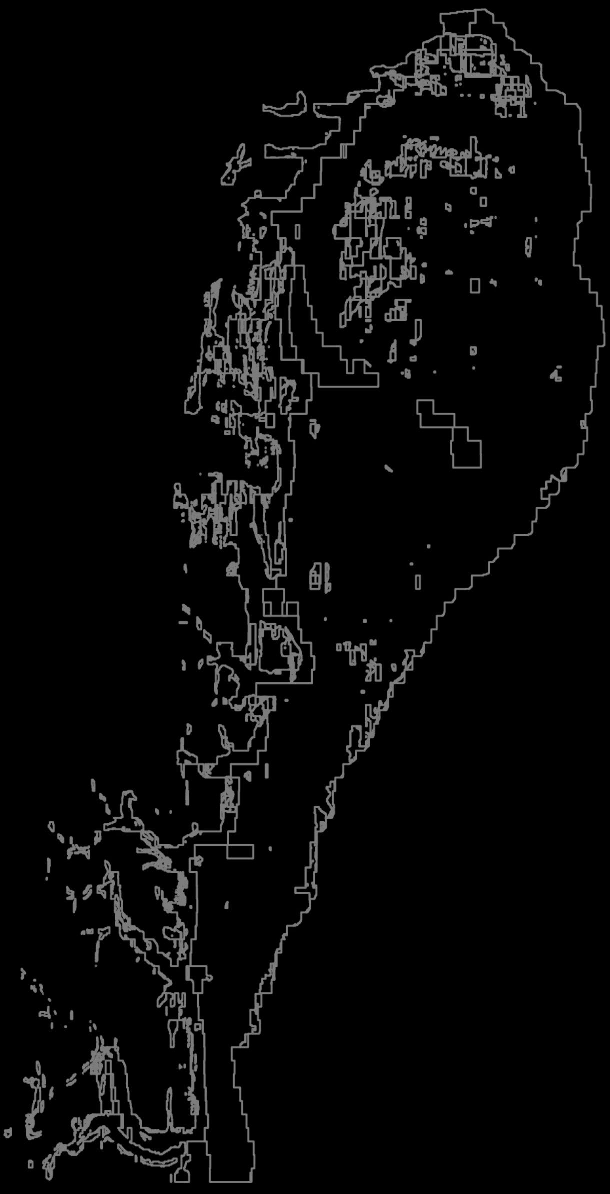

Supplement: Supplementary file 2 — Data S1. [file GCB-31-e70189-s002.pdf]
